# Facultative heterochromatin mediated by core and accessory chromosome-encoded H3K27-specific methyltransferases controls virulence in a fungal phytopathogen

**DOI:** 10.1093/nar/gkaf1441

**Published:** 2026-01-06

**Authors:** Slavica Janevska, Lucía Gómez-Gil, Tongta Sae-Ong, Umer Farooq, Lena Studt-Reinhold, Manuel Sánchez López-Berges, Gianni Panagiotou, Martijn Rep, Antonio Di Pietro

**Affiliations:** (Epi-)Genetic Regulation of Fungal Virulence, Leibniz Institute for Natural Product Research and Infection Biology – Hans Knöll Institute (Leibniz-HKI), Jena 07745, Germany; Molecular Plant Pathology, University of Amsterdam, 1098 XH Amsterdam, the Netherlands; Department of Genetics, University of Córdoba, Córdoba 14014, Spain; Microbiome Dynamics, Leibniz Institute for Natural Product Research and Infection Biology – Hans Knöll Institute (Leibniz-HKI), Jena 07745, Germany; (Epi-)Genetic Regulation of Fungal Virulence, Leibniz Institute for Natural Product Research and Infection Biology – Hans Knöll Institute (Leibniz-HKI), Jena 07745, Germany; Applied Genetics and Cell Biology, University of Natural Resources and Life Sciences, Vienna (BOKU), Campus Tulln, Tulln an der Donau 3430, Austria; Department of Genetics, University of Córdoba, Córdoba 14014, Spain; Microbiome Dynamics, Leibniz Institute for Natural Product Research and Infection Biology – Hans Knöll Institute (Leibniz-HKI), Jena 07745, Germany; Friedrich Schiller University Jena, Faculty of Biological Sciences, Jena 07743, Germany; Molecular Plant Pathology, University of Amsterdam, 1098 XH Amsterdam, the Netherlands; Department of Genetics, University of Córdoba, Córdoba 14014, Spain

## Abstract

In many fungal phytopathogens, infection is regulated by accessory genomic regions enriched in facultative heterochromatin, but the precise role of this chromatin state in virulence is poorly understood. Here we examined the two facultative heterochromatic marks H3K27me and H3K36me in the tomato-pathogenic *Fusarium oxysporum* isolate *Fol* 4287, by genetically analyzing the corresponding histone methyltransferases Kmt6 and Ash1. *Fol* has three *KMT6* homologs, two of which, *KMTB/C*, are encoded on an accessory chromosome. Loss of the core gene *KMT6A* resulted in strongly reduced but not abolished H3K27me, revealing that methylation of subtelomeres can be taken over by the accessory copy Kmt6b. To the best of our knowledge, this demonstrates for the first time that accessory fungal histone modifiers can contribute to the overall epigenetic profile. Deletion of *KMT6A* or *ASH1* resulted in derepression of *in planta*-induced effector genes under axenic conditions. *KMT6A* deletion mutants were blocked at early stages of root infection and were non-pathogenic on tomato plants, but were still able to kill the animal host *Galleria mellonella*. Constitutive overexpression of the pathogenicity-related transcription factor *FTF1* in the ∆*kmt6a* background restored effector gene activation *in planta* but not pathogenicity, suggesting that additional facultative heterochromatin-regulated virulence factors are essential for plant infection.

## Graphical Abstract

**Graphical Abstract:**
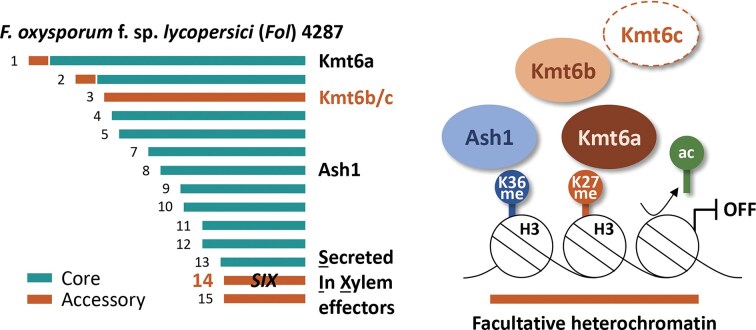

## Introduction

Members of the *Fusarium oxysporum* species complex (FOSC) are devastating vascular wilt pathogens, which infect a wide variety of agronomically important crops, including tomato, banana, cucurbits, cotton, date, and oil palms [1]. Additionally, some isolates cause opportunistic infections in humans [2, 3]. Concerning plant infections, pathogenicity of individual isolates is limited to one or a few plant species, and depends on a specific set of small secreted effectors called SIX (Secreted In Xylem), which determine the ability to colonize the vascular system and cause wilt disease [4]. The reference genome sequence of the tomato-infecting *F. oxysporum forma specialis* (f. sp.) *lycopersici* (*Fol*) isolate 4287 revealed the presence of accessory chromosomes that are lineage-specific, in contrast to the broadly conserved core chromosomes [5]. For *Fol* 4287, it was demonstrated that the small accessory chromosome 14, which encodes all SIX effectors, is necessary and sufficient for tomato infection [5]. Furthermore, chromosome 14 also encodes a Zn(II)_2_Cys_6_-type transcription factor named Ftf1, which acts as a positive regulator of the adjacent effector genes [6].

Histone modifications play a crucial role in the spatio-temporal control of pathogenicity-related gene expression. It is well-established that virulence-associated genes, such as effectors, are subject to tight transcriptional control, being repressed under axenic growth conditions and specifically upregulated *in planta* during infection [7, 8]. Although the mechanism of *in planta* gene induction remains enigmatic, part of the transcriptional control in accessory chromosomes and subtelomeric regions of core chromosomes in different fungi appears to be mediated by facultative heterochromatin, characterized by histone 3 lysine 27 methylation (H3K27me) [9–12]. In contrast to constitutive heterochromatin (H3K9me), which is found primarily at centromeres and transposable elements and destined for long-term gene silencing [13], facultative heterochromatin can become transcriptionally active under specific developmental or environmental conditions [7–10]. On the other hand, core genes are mostly found within euchromatic regions enriched in the H3K4me mark [12, 14]. Besides gene expression, epigenetic modifications control other processes such as nuclear localization and DNA repair [11]. Thus, a multi-speed genome model has been proposed, which distinguishes conserved euchromatic core regions from faster-evolving heterochromatic accessory regions [12, 15].

In filamentous fungi, including different *Fusarium* species, a single H3K27-specific methyltransferase named lysine methyltransferase 6 (Kmt6) has been described [9–11, 16]. In addition, two H3K36-specific methyltransferases with differential targets have been characterized: Set2 deposits the H3K36 modification in actively transcribed genes of euchromatin and inhibits internal transcriptional start sites *via* interaction with the elongating form of RNA polymerase II, while Ash1 targets facultative heterochromatic regions and has been implicated in gene silencing [17, 18]. How exactly the genes within facultative heterochromatin are repressed, and how derepression is initiated under activating conditions, is currently under investigation in different filamentous fungal models. Insights into these processes are important both for understanding the biology of fungal infections and for transferring this knowledge to more complex eukaryotic systems.

Here, we characterized H3K27- and H3K36-specific histone methyltransferases targeting facultative heterochromatin in *Fol* 4287. We report the presence of multiple functional H3K27-specific methyltransferases in a fungal species: Kmt6a encoded on the core and Kmt6b/c encoded on the accessory genome. Kmt6b can substitute for loss of Kmt6a and primarily methylates the subtelomeres of each chromosome ensuring more robust growth. Using a combination of RNA sequencing (RNA-seq) and chromatin immunoprecipitation coupled with sequencing (ChIP-seq), we establish that Kmt6a, as well as the H3K36-specific methyltransferase Ash1, contribute to silencing of genes located in facultative heterochromatic regions under non-inducing axenic conditions. In the case of Ash1, H3K36-mediated H4 deacetylation is likely involved in gene silencing. We further show that loss of *KMT6A* abolishes pathogenicity of *Fol* 4287 on tomato plants. Overexpression of the virulence-related accessory transcription factor (TF) *FTF1* in the ∆*kmt6a* background restores *in planta* expression of *SIX* effector genes but not virulence, suggesting that Kmt6a is required for the regulation of additional, essential virulence factors.

## Materials and methods

### Fungal strains, media, and growth conditions

Tomato-infecting *Fol* 4287 (NRRL_34936) [5] was used as wild type (WT) for the generation of gene deletion and overexpression mutants. Additionally, the endophytic biocontrol strain *Fo* 47 [19] harboring a single *KMT6* homolog was used for comparison. For *Fusarium graminearum* cross-complementation approaches, PH-1 (FGSC_9075, NRRL_31084) was used as a parental strain. Plate assays were done by inoculating mycelial plugs of standardized size and performed in biological triplicates. *Fusarium oxysporum* assays were conducted on solid potato dextrose agar (PDA; BD Difco, Le Pont de Claix, France), with 0-50 µg/ml doxycycline hyclate (Sigma–Aldrich, Taufkirchen, Germany), or on Czapek Dox agar (CDA; Oxoid, Fisher Scientific, Schwerte, Germany), incubating plates for 4 to 6 days at 25°C in the dark. *F. graminearum* assays were performed on solid Complete Medium CM [20], V8 (30 mM CaCO_3_, 20% v/v vegetable juice; Campbell Food, Puurs, Belgium), and *Fusarium* Minimal Medium (FMM) [21], incubating plates for 5 days at 20°C in the dark. Prior to gDNA isolation, or to protein extraction from doxycycline-containing plates, the strains were grown on PDA covered with a layer of cellophane for 2 or 4 days, respectively, under the same conditions. Cultivation in liquid cultures for protein extraction, RNA extraction, or ChIP was performed in biological replicates and started with a 100-ml Darken pre-culture [22] in 300-ml Erlenmeyer flasks, which were inoculated with 2 mycelial plugs and shaken for 3 days at 180 rpm and 25°C in the dark. For *Fol* 4287, 500 µl of the pre-culture were transferred to 100 ml NO_3_ main culture, consisting of 1% (w/v) KNO_3_, 3% (w/v) sucrose, and 0.17% (w/v) yeast nitrogen base without amino acids and ammonium sulfate, and shaken for another 2 days under the same conditions. For *F. graminearum*, ICI (Imperial Chemical Industries Ltd., London, UK) [23] with 60 mM glutamine was inoculated as main culture and shaken for 3 days. For the collection of microconidia, 5-day-old NO_3_ main cultures were filtered over miracloth, conidia were washed with 30 ml of sterile H_2_O and taken up in 10 ml H_2_O before counting. For protoplast transformation of *Fol* 4287, 500 µl of the pre-culture were transferred to 100 ml ICI medium with 6 mM glutamine as nitrogen source and grown for no longer than 16 h.

### Plasmid constructs and generation of fungal transformants

Yeast recombinational cloning [24, 25] was employed for the construction of deletion, (cross-)complementation, inducible *TET^OFF^*, and constitutive overexpression vectors. For assembling gene deletion vectors, ca. 1 kb 5′-upstream and 3′-downstream sequences of the genes of interest were amplified from gDNA with primer pairs 5F/5R and 3F/3R, introducing the required overhangs (Supplementary Table S1). The two employed resistance cassettes, *hphR* (hygromycin B phosphotransferase gene) and *natR* (nourseothricin resistance gene), both under control of the *Aspergillus nidulans trpC* promoter, were amplified with hph_F/hph_R (Supplementary Table S1) from the templates pCSN44 [26] or pZPnat1 (GenBank AY631958), respectively. The yeast strain *Saccharomyces cerevisiae* BY4741 (Euroscarf, Oberursel, Germany) was transformed with the obtained PCR fragments as well as with the XbaI/HindIII-digested shuttle vector pYES2 (Life Technologies, Darmstadt, Germany), yielding the deletion constructs ∆*ash1::hphR* (Supplementary Fig. S1A), ∆*kmt6a::hphR* (Supplementary Fig. S2A), ∆*kmt6b::hphR* (Supplementary Fig. S3A), and ∆*kmt6c::natR* (Supplementary Fig. S3C).

For the cloning of the *in locus* complementation constructs *ASH1^C^* (Supplementary Fig. S1C) and *KMT6A^C^* (Supplementary Fig. S2C), the full-length genes, including 1 kb of the 5′-flanking promoter sequences, were amplified with Ash1_5F/Ash1_compl_R and Kmt6a_5F/Kmt6a_compl_R (Supplementary Table S1), respectively. 3′ sequences were obtained as described earlier using 3F/3R primers, while the *natR::TtrpC* cassette was amplified with hphR/TtrpC_R from pZPnat1. These fragments were cloned into double-digested pYES2 as described above. For cross-complementation of *KMT6B* or *KMT6C* at the *KMT6A* locus (Supplementary Fig. S2E), the genes were amplified with Pkmt6a_Kmt6b_F/R or Pkmt6a_Kmt6c_F/R and fused to the *KMT6A* promoter amplified with Kmt6a_5F/Kmt6a_5R_short. *KMT6A* 3′ flank and *natR* resistance cassette were gained as above.

Constructs for *FgKMT6* (*FGSG_15795*) complementation and cross-complementation (Supplementary Fig. S4A) were generated as follows: For complementation of ∆*fgkmt6* [27], the native gene was amplified together with 1 kb upstream region with primer pairs FgKMT6_Cil_XbaI_F1/KMT6_Cil_1R and KMT6_Cil_2F/KMT6_Cil_2R (Supplementary Table S1) from *Fg* gDNA, while for cross-complementation the native promoter of *FgKMT6* was fused to either *FolKMT6A, FolKMT6B*, or *FolKMT6C* using primer pairs FgKMT6_Cil_XbaI_F1/FolKMT6A-C_CCil_1R and either FolKMT6A_CCil_2F/2R, FolKMT6B_CCil_2F/2R, or FolKMT6C_2F/2R, respectively, from either *Fg* gDNA or *Fol* 4287 gDNA. The individual genes were followed by *BcTgluc* and the geneticin resistance cassette (*genR*) amplified with Tgluc_F2/Gen_gpd_F from pΔ*fmkmt1*/*FmKMT1^Ces^* [28], and 1 kb of *FgKMT6* downstream region amplified with FgKMT6_Cil_3F/FgKMT6_Cil_XbaI_3R from *Fg* gDNA. The correct assembly into EcoRI/XhoI-double digested pRS426 [29] was verified by sequencing using primers listed in Supplementary Table S1.

For the cloning of the inducible knockdown constructs *TET^OFF^::KMT6A* (Supplementary Fig. S5A) and *TET^OFF^::SUZ12* (Supplementary Fig. S5E), the first 1 kb of the genes of interest were amplified with Kmt6a_TET_3F/Kmt6a_TET_3R and Suz12_TET_3F/Suz12_TET_3R (Supplementary Table S1) from *Fol* 4287 gDNA, respectively. 5′ sequences were amplified using 5F/TET_5R primers, while the *hphR::TtrpC* cassette was amplified with hphR/TtrpC_R from pCSN44. The *TET^OFF^* construct, including the tetracycline-controlled transactivator (*tTA*) gene under control of *A. nidulans PgpdA* and the tTA-responsive operator sequence (*TetO7*), was amplified from pFW9 [30] using primers hphR_PgpdA_F/TET_off_R. Moreover, a *TET^OFF^::KMT6A* construct with the geneticin cassette was cloned for generating the triple *KMT6A-C* mutant (Supplementary Fig. S5C), for which the geneticin resistance gene (pKS-Gen [31]) was fused to *PtrpC* and *TtrpC* with following PCRs: hph_F/PtrpC_R, gen_PtrpC_F/gen_TtrpC_R, and TrpC_F/TtrpC_R. Again, the indicated fragments were cloned into pYES2 as described earlier.

For the cloning of the constitutive overexpression vectors, the base vector pDDN-OGG [32] was employed, which directs the overexpression construct to the constitutively expressed locus *DDR48*/*FOXG_02247* [32, 33]. pDDN-OGG with the *natR* resistance cassette was used to clone the base vector pDDH-OGG with the *hphR* cassette. To this end, the backbone was amplified with Ddr_3F/Ddr_5R from pDDN-OGG, while the *hphR*-containing insert was amplified with hphR_PmeI_R/Tgluc_R from pNDH-OGG [25], prior to yeast transformation. For pOE::*ASH1::FLAG* (Supplementary Fig. S6A), the gene of interest was amplified with Ash1_OE_F/Ash1_FLAG_R (Supplementary Table S1), and FLAG was inserted by annealing primers FLAG_Tgluc_F/FLAG_Tgluc_R (5 min at 95°C, annealing at room temperature), both of which were transformed into yeast together with NcoI/NotI-digested pDDH-OGG. For pOE::*FTF1::FLAG* (Supplementary Fig. S6C), the gene of interest was amplified with Ftf1_OE_F/Ftf1_FLAG_R, while double-stranded FLAG was prepared as above, both of which were transformed into yeast together with NcoI/NotI-digested pDDN-OGG. The correct assembly of all vectors was verified by sequencing with primers listed in Supplementary Table S1.

Protoplast transformation of *Fol* 4287 and *Fo* 47 was performed as previously described [34]. In the case of *F. graminearum*, a slightly adjusted protocol was used as described elsewhere [35]. Constructs for transformation were polymerase chain reaction (PCR)-amplified from the earlier described, cloned plasmids using 5F/3R primer combinations (Supplementary Table S1). Transformants were selected on 100 µg/ml hygromycin B (InvivoGen Europe, Toulouse, France), 150 µg/ml nourseothricin (Jena Bioscience, Jena, Germany), or 100 µg/ml geneticin. The homologous integration of the transformed constructs was verified by diagnostic PCR, showing the correct recombination of the 5′ and 3′ flanks, and the absence of untransformed nuclei for 2–7 independent transformants (primers listed in Supplementary Table S1 and in Supplementary Fig. S1B and D, Supplementary Fig. S2B, D, and F, Supplementary Fig. S3B, D, and F, Supplementary Fig. S5B, D, and F, and Supplementary Fig. S6B and D). Additionally, it was made sure that the complemented transformants (Supplementary Figs S1D and S2D and F) were unable to grow on hygromycin B (deletion phenotype). In the case of the cross-complemented *F. graminearum* transformants, both absence of growth on hygromycin B and expression of the respective gene were confirmed (Supplementary Fig. S4B).

### Phylogenetic analysis

*Fusarium* Kmt6 homologs were obtained by BLASTp search [36] with *Fol* 4287 Kmt6a as bait, using *Neonectria* as sister taxon, and *Neurospora* and *Beauveria* as outgroups. Amino acid alignments were done with the MUSCLE algorithm, after which the maximum-likelihood method was applied for tree building, with bootstrap values based on 1 000 pseudoreplicates, as implemented in the DNASTAR Lasergene program MegAlign Pro (Madison, WI, USA).

### Expression analysis under axenic growth conditions

Mycelium was freeze-dried and ground prior to RNA extraction using the TRI Reagent (Sigma–Aldrich, Taufkirchen, Germany). For expression analysis *via* reverse transcription quantitative PCR (RT-qPCR), 1.5 µg of RNA was DNase I-treated and transcribed into complementary DNA (cDNA) using the ProtoScript II First Strand cDNA Synthesis Kit (New England Biolabs, Frankfurt/Main, Germany) and the included oligo(dT) primers according to the standard protocol. For RT-qPCR, MyTaq HS Mix (Biocat, Heidelberg, Germany) was used together with 5% (v/v) EvaGreen Dye (Biotium, Fremont, CA, USA) on an Agilent Mx3000P qPCR System with the respective Agilent plastics (Santa Clara, CA, USA). Expression of the genes of interest as well as of the constitutively expressed reference gene (*FOXG_01569*/*FoACT* and *FGSG_07335*/*FgACT*, encoding actin) was determined in triplicate with 3′ primers listed in Supplementary Table S1. The annealing temperature was set to 60°C, and primer efficiencies were between 90% and 110%. Relative expression (relative to actin) and gene copy number (relative to *EF1α*) were calculated with the ∆Ct method [37].

For the RNA-seq analysis, *Fol* 4287, ∆*ash1* T20, and ∆*kmt6a* T11 were grown in biological quadruplicates in NO_3_ for 2 days, prior to RNA extraction as above. Quality control, library preparation with 3′ polyA enrichment, and single-end Illumina sequencing 1 × 75 bp (NextSeq High Output v2.5), giving ca. 400 million reads for all 12 samples, were performed by the Sequencing Facility at the University of Amsterdam. Moreover, four biological replicates of *Fol* 4287 grown under the same conditions were subjected to paired-end sequencing (2 × 150 bp, NovaSeq6000 sequencing platform at Novogene, UK) to be compared with the *in planta* paired-end samples.

### ChIP and western blot analysis

For the ChIP-seq analysis, *Fol* 4287, ∆*ash1* T20, and ∆*kmt6a* T11 were grown in biological duplicates in NO_3_ for 2 days, and *F. graminearum* strains were grown in ICI + 60 mM glutamine for 3 days, prior to crosslinking with formaldehyde (1%, v/v, final concentration) for 15 min at 25°C and 90 rpm, and subsequent quenching with glycine (125 mM final concentration) for 5 min at 37°C. The mycelium was harvested, shock-frozen, and ground to fine powder under the use of liquid nitrogen. Further sample preparation was essentially performed as previously described [17, 38]. Briefly, the chromatin was sheared using the Bioruptor Pico (Diagenode, Seraing, Belgium), yielding DNA fragments with an average size of 150–250 bp. Antibodies (Active Motif, La Hulpe, Belgium) against H3K36me3 (#61101) and H3K27me3 (#39155), in combination with Dynabeads Protein A (Fisher Scientific, Schwerte, Germany), were applied for precipitation of the chromatin-antibody-conjugate. Input samples were not treated with antibody, and one input sample per strain, together with the *Fol* ChIP duplicates, were subjected to quality control, library preparation, and Illumina sequencing (1 × 75 bp, NextSeq High Output v2.5, ca. 400 million reads for 19 samples) at CRG Barcelona (Centre de Regulació Genómica, Spain). The *F. graminearum* ChIP-seq (2 × 150 bp, NovaSeq6000 sequencing platform) was done at Novogene, UK.

ChIP-qPCR was performed in triplicate, with primers that bind at the 5′ gene ends listed in Supplementary Table S1. Next to the antibodies indicated above, the following antibodies were also used: anti-H3ac (#39040), anti-H4ac (#39026), anti-H4K5ac (#39699), anti-H4K8ac (#61103), anti-H4K12ac (#39066), anti-H4K16ac (#39068; all from Active Motif, La Hulpe, Belgium), and anti-FLAG (#F7425; Sigma–Aldrich, Taufkirchen, Germany).

Western blot analysis was performed as previously described [10, 17, 39]. Ten micrograms (H3K36me3) and forty micrograms (H3K36me2, H3K27me3, H3 C-terminal, H3ac, H4, and H4ac) of total protein extracts were separated *via* sodium dodecyl sulfate–polyacrylamide gel electrophoresis, and after semi-dry blotting were incubated with the following primary antibodies (Active Motif, La Hulpe, Belgium) at indicated dilutions: anti-H3K36me3 (1:10 000), anti-H3K36me2 (#39891 at 1:5000), anti-H3K27me3 (1:5000), and anti-H3 C-terminal (#39163 at 1:10 000). The above-mentioned anti-H4ac antibodies were used at 1:5000, together with the control anti-H4 (#39270 at 1:5000). Goat anti-rabbit IgG-HRP (#31460, Fisher Scientific, Schwerte, Germany, or Jackson ImmunoResearch Europe Ltd., Cambridgeshire, UK) served as secondary antibody at a 1:5000 dilution. Relative band intensities of the representative blots shown were quantified using photo editing software (Adobe Photoshop, San Jose, CA).

### Data analysis

ChIP-seq and RNA-seq data were pre-processed using the following steps. Low-quality reads and adapters were removed from sequencing data using Trim Galore v.0.6.7 [40] with default parameters. The high-quality reads were mapped against the *Fol* 4287 assembled genome (GCA_001703175.2) with mismatch count calibration performed (-k) and were sorted using Novoalign v3.09.00 (http://novocraft.com/). *In planta* RNA-seq reads quality across all replicates was checked with FastQC v.0.12.1 (www.bioinformatics.babraham.ac.uk/projects/fastqc/), and low-quality reads and adapters were removed from sequencing data using FastP v.0.23.4 [41]. High-quality reads were mapped simultaneously against the *Fol* 4287 (GCA_001703175.2) and tomato SL3.0 (ENSEMBL Plants) genome assemblies using bbsplit.sh (from BBmap v.39.13 [https://sourceforge.net/projects/bbmap/]) [42] to extract reference-specific reads, which were then mapped against the respective genome using Hisat2 v.2.2.1 [43]. In each case, Samtools v.1.17 [40] was used to convert SAM files to BAM files. *F. graminearum* ChIP-seq reads were aligned to its genome assembly (GCF_000240135.3) using bowtie2 v.2.5.4 [44]. PCR duplicates were removed from *F. graminearum* BAM files using picard v.3.4.0 (http://broadinstitute.github.io/picard).

RNA-seq read abundance was calculated using featureCount from Rsubread version 2.0.8 [45]. Read abundance was imported to RStudio (R version 4.4.2) [46] for statistical analyses. The “Median Ratio Normalization” (MRN) method [47] was employed to normalize read counts across all samples. Differentially expressed genes (DEGs) were examined as pairwise differences between ∆*ash1* versus WT and ∆*kmt6a* versus WT under axenic conditions and ∆*kmt6a* versus WT *in planta*, and *in planta* WT versus axenic WT, using DESeq2 package version 1.46.0 [48]. Significant DEGs were chosen based on their log_2_-fold change (log_2_FC) and false discovery rate-corrected *P* value (FDR-corrected *P*). Significant DEGs with |log_2_FC| ≥ 4 and FDR-corrected *P* ≤ .01 were further used for the evaluation of the RNA-seq and its functional annotation, while significant DEGs with |log_2_FC| ≥ 2 and FDR-corrected *P* ≤ .01 were used for comparing significant genes between RNA-seq and ChIP-seq analyses. For the *in planta* RNA-seq, significant DEGs with |log_2_FC| ≥ 1 and FDR-corrected *P* ≤ .01 were used for evaluation and functional annotation.

ChIP-seq read coverage and enrichment were visualized after converting BAM files to TDF files using igvtools v.2.3.93 [49]. The mapped BAM files were converted to BigWig format *via* bamCoverage from deeptools v.3.5.6 [50] using “Read Per Genome Coverage (RPGC; 1× depth)” normalization with parameters “--effectiveGenomeSize 56213053 (36458046 for *F. graminearum*) --binSize 10 --extendReads 150 --smoothLength 150 --centerReads”. The means of normalized coverages of all replicates were calculated with Wiggletools v.1.2.11 [51]. Wig files were converted to BigWig format with UCSC Genome Browser tools. Peaks were called using findPeaks from HOMER v.4.11 [52] with parameters “--style histone --region --size 500 --minDist 1000 --C 0″. Peak lengths were split into 500 bp windows using bedtools v2.31.1 [53]. Heatmaps for all peaks were generated using computeMatrix scale-regions mode from deeptools with “--regionBodyLength 500″ [54]. Peaks were differentially analyzed and annotated to genomic features using “getDifferentialPeaks” and “annotatePeaks.pl” from HOMER. Fold enrichment over background ≥4 and Poisson enrichment *P* value ≤.0001 were applied to select significantly differential peaks.

Functional annotation was performed using InterProScan v5.73 [55] and KOFamScan v.1.3.0 [56] with e-value = 10^−5^. ClusterProfiler v4.14.0 package [57] from R was employed for enrichment analysis.

### Fungal growth under *in vitro* stress conditions

For phenotypic analysis of colony growth, droplets from serial dilutions containing 10^5^, 10^4^, and 10^3^ microconidia were spot-inoculated on Yeast extract Peptone Dextrose Agar plates [YPDA; 0.3% (w/v) yeast extract, 1% (w/v) peptone, 2% (w/v) glucose, 1.5% (w/v) agar]. For cell wall and membrane stress assays, calcofluor white (CFW; 50 µg/ml; Sigma–Aldrich, St. Louis, Missouri, USA), Congo red (CR; 100 µg/ml; Sigma–Aldrich, St. Louis, Missouri, USA), or sodium dodecyl sulfate [SDS; 0.0125% (w/v); Merck KGaA, Darmstadt, Germany] were added to the YPDA medium. For hyperosmotic stress, YPDA plates were supplemented with sodium chloride (NaCl; 1.2 M; Merck KGaA, Darmstadt, Germany) or sorbitol (1.25 M; Thermo Fisher Scientific, Waltham, Massachusetts, USA). Plates were incubated for 3 days at 28°C and scanned. Experiments were performed three times with similar results.

### Cellophane penetration assay

Invasive hyphal growth on cellophane membranes (colorless; Manipulados Margok S.A.L., Gipuzkoa, Spain) was determined as previously described [58] with some modifications. 5 × 10^4^ freshly obtained microconidia were spot-inoculated on a cellophane membrane placed on Puhalla’s Minimal Medium plates (PMM) [59] buffered with 2-(*N*-morpholino)ethanesulfonic acid (MES; 0.1 M; Sigma–Aldrich, St. Louis, Missouri, USA) to pH 5 or pH 7 with sodium hydroxide (NaOH; 10 M; Merck KGaA, Darmstadt, Germany). After 4 days of incubation at 28°C, the cellophane membrane with the fungal colony was removed, and plates were incubated for an additional day to visualize mycelial growth indicative of cellophane penetration. Experiments were performed in triplicate.

### Tomato pathogenicity assays

Two types of tomato pathogenicity assays were performed. In the first assay (Fig. 8), the tomato (*Solanum lycopersicum*) cultivar “C32,” susceptible to race 2, was used [60]. The roots of 10-day-old tomato seedlings (10 per treatment, independent biological duplicates) were trimmed (1 cm remaining) before being dipped into a fungal spore suspension of 10^7^ conidia/ml (in water) for 2 min, using water as mock control, and planted in pots with soil [61]. Plants were grown in a climate-controlled greenhouse at 25°C, 65% relative humidity, and a 16 h light/8 h dark regime for 3 weeks. Then, fresh weight and disease index were scored as follows [62]: 0, no visible symptoms; 1, one brown vessel below the cotyledons; 2, one or two brown vascular bundles at cotyledons; 3, three brown vascular bundles and growth distortion; 4, all vascular bundles are brown, with the plant either dead or very small and wilted. Kruskal–Wallis test was performed for statistical analysis, as implemented in GraphPad Prism (Boston, MA, USA), comparing the other treatments to the WT *Fol* 4287.

For the second type of tomato infection assay (Fig. 9), tomato seeds (*S. lycopersicum* , cultivar Moneymaker F1 hybrid from EELM-CSIC, Malaga, Spain; susceptible to race 2) were surface-sterilized by immersing them in 1% (v/v) sodium hypochlorite for 30 min, sown in moist vermiculite (Turbas y Coco Mar Menor S.L., Sucina, Murcia, Spain), and incubated for 14 days in a growth chamber under the following conditions: 28°C, 40%–70% relative humidity, and a photoperiod of 14 h of 36 W white fluorescent light and 10 h of darkness. Tomato plant infection was performed as previously described [63], by dipping roots of 14-day-old tomato seedlings into a fungal suspension of 5 × 10^6^ microconidia/ml of the different fungal strains. Plant survival was recorded daily up to 35 days post inoculation (DPI). Complete stem collapse, absence of green parts, and proliferation of fungal mycelium in the dead tissue indicated plant death. Mortality curves were plotted by the Kaplan–Meier method and plant survival was compared among groups using the log-rank statistical test with the software GraphPad Prism v.8.0.1 (Boston, MA, USA) [64]. Plant infection assays were performed in triplicate, including 10 plants per treatment.

### *In planta* fungal burden and gene expression

Gene expression and fungal burden in tomato plants were measured by qPCR as previously described [65]. For quantification of fungal burden, DNA was extracted from infected tomato roots of 3 plants at 4 and 10 DPI using a modified chloroform:octanol extraction method [66]. For gene expression analysis, RNA was isolated from roots of 5 tomato plants at 6 DPI using the TriPure RNA isolation reagent (Roche Diagnostics GmbH, Mannheim, Germany) with DNase I treatment (Roche Diagnostics GmbH, Mannheim, Germany) and transcribed into cDNA using the iScript cDNA Synthesis Kit (Bio-Rad, California, USA). Relative fungal burden was calculated using the ∆Ct method [37] with the *Fol* 4287-specific gene *SIX1* (*FOXG_16418*) and normalized to the reference tomato *GAPDH* gene (*U93208*). Relative transcript levels of tomato defense genes *PR1* (*K4CBE2*) and *CHI3* (*Z15141*) were measured by RT-qPCR and normalized to the constitutive reference tomato *SlACT* gene (*BT012695*) [67]. Transcript levels of the fungal virulence-associated *SIX1 (FOXG_16418), SIX3* (*FOXG_16398*), and *FTF*-type genes (here characterized as the homolog *FTF1*/*FOXG_17458*), *ERC1* effector gene (*FOXG_11583*) [68], and *FOXG_00543* and *FOXG_11153* were normalized to the *Fol* 4287-specific constitutive gene *FoACT* (*FOXG_01569*). Statistical significance was calculated according to the unpaired *t*-test with the software GraphPad Prism v.8.0.1 (Boston, MA, USA). For *in planta* RNA-seq analysis, RNA was extracted as above and treated with DNase I using the Turbo DNA-Free Kit (Invitrogen). Quality control, library preparation with 3′ polyA enrichment, and paired-end Illumina sequencing 2 × 150 bp (NovaSeq6000 sequencing platform), were performed by Novogene, UK.

### *Galleria mellonella* pathogenicity assays

*Galleria mellonella* infection assays were performed as described [69]. *G. mellonella* larvae between 0.2 and 0.3 g in weight (CASA REINA S.A., Bilbao, Spain) were inoculated with a 1 ml syringe to inject 8 μl of a 1.6 × 10^4^ microconidial suspension (in sterile phosphate-buffered saline, PBS 1×) into the haemocoel of each larva, using a Burkard auto microapplicator (Burkard Manufacturing Co. Limited, Hertfordshire, UK). Non-injected and PBS-injected larvae were used as control treatments. Larvae were incubated in glass containers at 30°C, and the number of dead larvae was scored daily for 12 DPI. Larvae were considered dead when they were melanized and displayed no movement. Mortality curves were plotted by the Kaplan–Meier method and percentage survival was compared among groups using the log-rank statistical test with the software GraphPad Prism v.8.0.1 (Boston, MA, USA) [64]. *G. mellonella* pathogenicity assays were performed two times, including 15 larvae per treatment.

## Results

### *Fol* 4287 encodes multiple H3K36- and H3K27-specific methyltransferases

**Figure 1.**
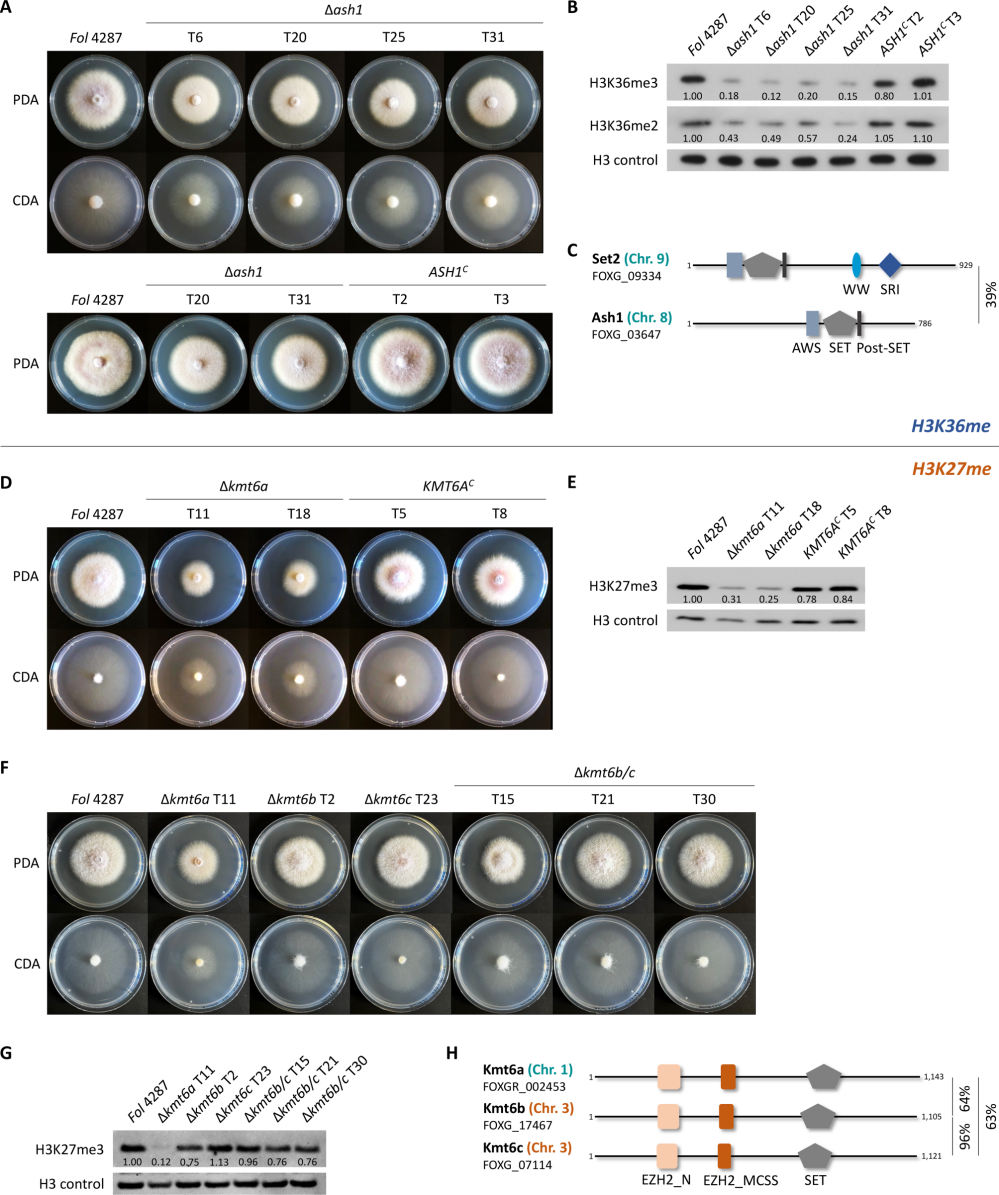
Analysis of multiple H3K36- and H3K27-specific methyltransferases in *Fol* 4287. Deletion of *ASH1*, encoding a H3K36-specific methyltransferase, results in slightly reduced vegetative growth on complex (PDA) and minimal (CDA) media (*n* = 3) (**A**), and in a genome-wide reduction of H3K36 methylation levels as detected by western blot analysis with specific antibodies (**B**). Bands were quantified by densitometry and normalized to the H3 control. (**C**) Domain structure of the two H3K36-specific methyltransferases Set2 and Ash1. Deletion of *KMT6A*, encoding an H3K27-specific methyltransferase, results in reduced colony growth on PDA and CDA (*n* = 3) (**D**), and in a genome-wide reduction of H3K27me3 levels (**E**). Double deletion of *KMT6B/C* does not affect vegetative growth (*n* = 3) in two out of three independent transformants (**F**), nor genome-wide H3K27me3 levels (**G**). (**H**) Domain structure of the three H3K27-specific methyltransferases Kmt6a-c. Quantification of colony diameters can be found in Supplementary Fig. S7A–C.

To examine the role of facultative heterochromatin in *F. oxysporum*, we performed combined BLASTp and BLASTn searches with the sequences of the previously characterized H3K36-specific methyltransferase Ash1 (FFUJ_05655) [17] or the H3K27-specific methyltransferase Kmt6 (FFUJ_00719) [10] from *F. fujikuroi*. The first search identified a single Ash1 homolog in *Fol* 4287, FOXG_03647, whose deletion mutants showed a slight growth defect on complex and minimal media, which was restored by *in locus* re-introduction of the full-length WT allele in the complemented *ASH1^C^* strains (Fig. 1A). Western blot analysis revealed reduced genome-wide H3K36me3 and me2 in the ∆*ash1* mutants (Fig. 1B). In the Δ*ash1* background, the remaining H3K36-specific methyltransferase is Set2 (FOXG_09334) (Fig. 1C), homologous to *F. fujikuroi* FFUJ_08690 [17]. Set2 and Ash1 share the catalytic SET [Su(var)3–9, Enhancer-of-zeste, Trithorax] domain, as well as Post-SET and AWS (Associated with SET) domains, while Set2 harbors additional domains, i.e. the WW/Rsp5/WWP domain with two conserved tryptophan residues and the RNA polymerase II interaction domain SRI (Set2 Rbp1 interacting) (Fig. 1C).

Unexpectedly, the search for H3K27-specific methyltransferases in *Fol* 4287 revealed the presence of three Kmt6 homologs, which is in stark contrast to the single homologs reported in previously characterized fungi, including several *Fusarium* species such as *F. fujikuroi* [10], *F. proliferatum* [16], and *F. graminearum* [9]. The conserved core Kmt6 homolog, designated Kmt6a, is encoded on chromosome 1 (Fig. 1H). Due to an assembly error in the original annotation, the *KMT6A* gene lacks the original FOXG identifier, but is listed under *FOXGR_002453* in a more recent assembly and annotation [70, 71]. In addition to Kmt6a, we identified two additional Kmt6 homologs designated Kmt6b (FOXG_17467) and Kmt6c (FOXG_07114), both encoded on the accessory chromosome 3. Kmt6b and Kmt6c share 98% nucleotide homology in the coding sequence and >99% identity in sequences directly upstream (260 bp) and downstream (790 bp) of the genes (Supplementary Fig. S3).

**Figure 2.**
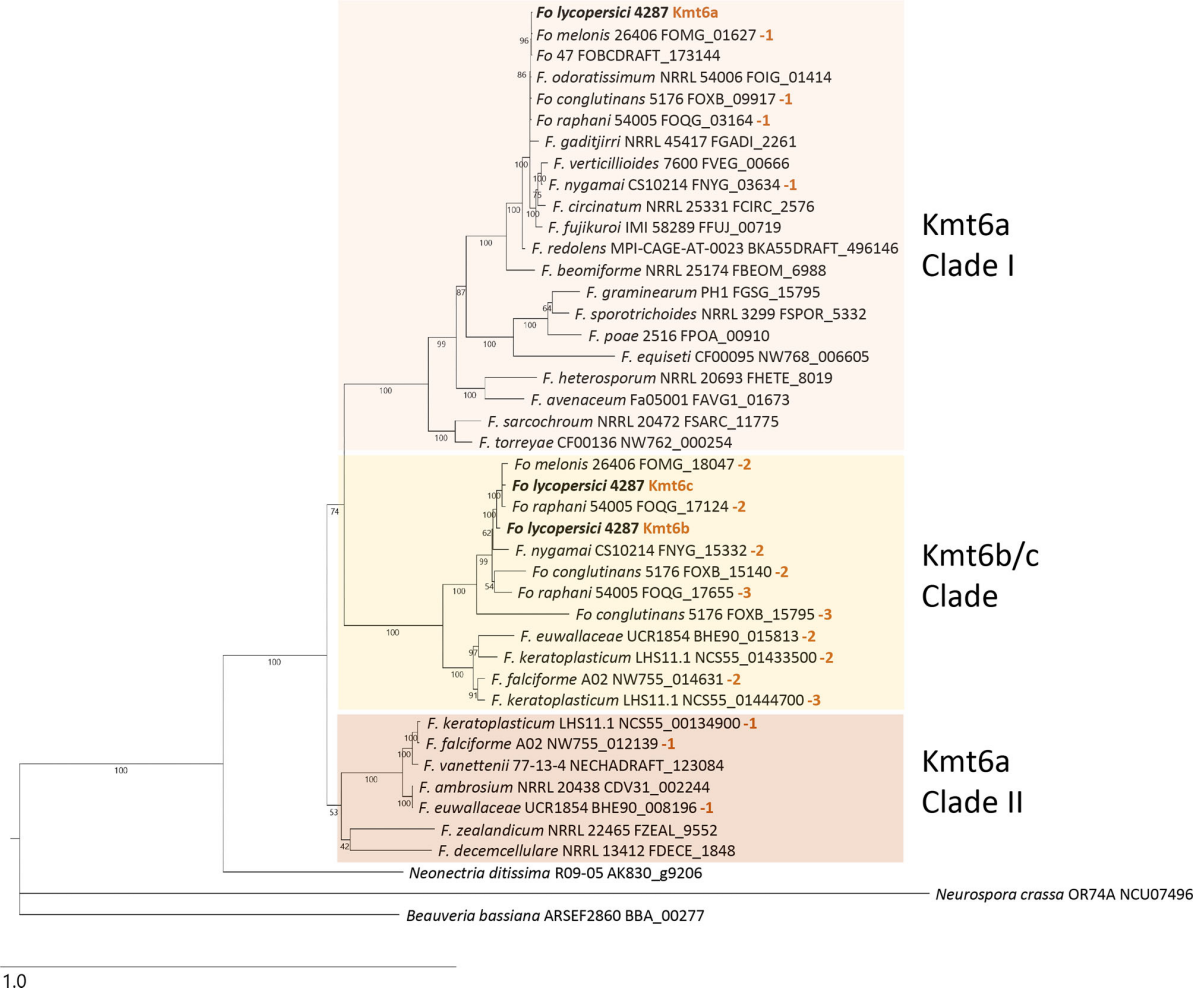
Phylogenetic tree of Kmt6 homologs in *Fusarium* spp. The phylogeny shows two major Kmt6a clades and one clade of Kmt6b/c homologs. In the cases where multiple Kmt6b/c homologs were found (indicated by −2, −3 in orange), the strain’s core homolog is marked with −1 (in orange). Absence of an orange number indicates the presence of a single Kmt6a homolog. The tree was inferred by maximum-likelihood analysis of alignments of predicted amino acid sequences of selected *Fusarium* species, using *Neonectria* as sister taxon, and *Neurospora* and *Beauveria* as outgroups. Accession numbers for protein sequences are indicated after species names. Numbers near branches are bootstrap values based on 1000 pseudoreplicates.

To understand the evolutionary origin of the multiple Kmt6 homologs in *Fol* 4287, we performed phylogenetic analysis in representative *Fusarium* species. Besides *Fol* 4287, multiple Kmt6 homologs were also detected in several members of the FOSC and the *F. solani* species complex (FSSC), as well as in *F. nygamai*, which belongs to the *F. fujikuroi* species complex (FFSC) (Fig. 2). Within the FOSC, multiple homologs were identified in *F. oxysporum* (*Fo*) f. sp. *melonis* (two homologs), *Fo* f. sp. *conglutinans*, and *Fo* f. sp. *raphani* (three homologs each), while only a single homolog was detected in the endophyte *Fo* 47 and the banana pathogen *Fo* f. sp. *cubense* TR4 (i.e. *Fusarium odoratissimum*). The conserved core homologs of Kmt6 clustered in two separate clades (named Kmt6a Clades I and II, Fig. 2), which were overall in line with the phylogenetic relationship between the *Fusarium* species complexes [72]. By contrast, the accessory homologs of the FOSC, FSSC, and FFSC all clustered in a single clade (named Kmt6b/c Clade), suggesting a more recent common evolutionary origin from an ancestor closer to Kmt6a Clade I (Fig. 2).

### Accessory homologs *KMT6B/C* are functional in the Δ*kmt6a* background

Deletion mutants in *KMT6A* (Δ*kmt6a*) were generated and subsequently complemented by re-introducing the WT allele (*KMT6A^C^*). Deletion of *KMT6A* resulted in significantly reduced vegetative growth (Fig. 1D and Supplementary Fig. S7B) and a distinct orange pigmentation of the colonies, which is most likely due to the upregulation of the 4 clustered genes *carX, carRA, carB*, and *carO* responsible for carotenoid biosynthesis (Supplementary Fig. S8A) [73]. Western blot analysis of Δ*kmt6a* mutants with anti-H3K27me3 antibody revealed strongly reduced but still detectable levels of H3K27me3 compared to the WT (Fig. 1E). We speculated that the residual H3K27me3 signal is due to the activity of the *KMT6B* and *KMT6C* genes. In line with this idea, we detected increased *KMT6B/C* expression in the Δ*kmt6a* mutant, although the transcript levels were still lower than those of *KMT6A* in the WT (Supplementary Fig. S9A). Individual deletion of *KMT6B* or *KMT6C* had no detectable effect on colony growth (Fig. 1F and Supplementary Fig. S7C) or genome-wide H3K27me3 levels (Fig. 1G). Both gene copies were found to contribute to the combined *KMT6B/C* transcript (Supplementary Fig. S9A). Furthermore, two out of three independent ∆*kmt6b/c* double deletion mutants failed to exhibit significant alterations in vegetative growth (Fig. 1F and Supplementary Fig. S7C) or in genome-wide H3K27me3 levels (Fig. 1G). This is in line with the previous observation that Kmt6a is responsible for the main fraction of H3K27me3 in *Fol* 4287 (Fig. 1E and G).

**Figure 3.**
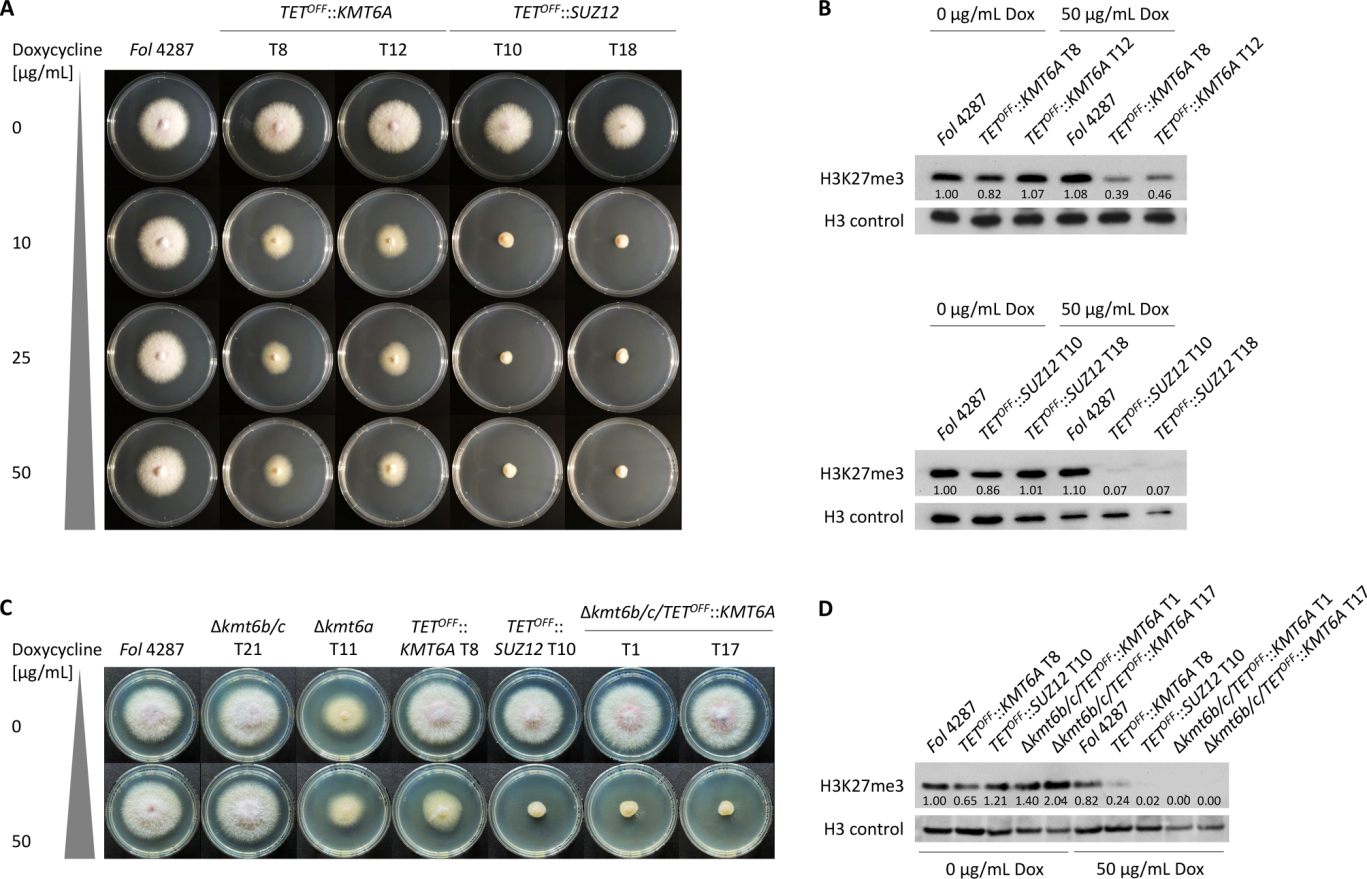
Suz12 is a unique PRC2 complex partner, while Kmt6a, Kmt6b, and Kmt6c have partially redundant H3K27me3 methyltransferase activity. (**A**) *Fol* 4287 WT and *TET^OFF^::KMT6A* or *TET^OFF^::SUZ12* mutants were grown on PDA with increasing concentrations of doxycycline (Dox) to downregulate *KMT6A* or *SUZ12* expression (*n* = 3). (**B**) Genome-wide H3K27me3 levels are decreased in *TET^OFF^::KMT6A* and largely abolished in *TET^OFF^::SUZ12*, as shown by western blot analysis with the specific antibody. Bands were quantified by densitometry and normalized to the H3 control. Downregulation of *KMT6A* in the Δ*kmt6b/c* background mimics the colony growth (*n* = 3) (**C**) and genome-wide H3K27me3 phenotype (**D**) of the *TET^OFF^::SUZ12* strain.

Next, we attempted to generate loss-of-function mutants of Suz12, which is part of the *Fusarium* Polycomb Repressive Complex 2 (PRC2) [74]. The single Suz12 homolog of *Fol* 4287 is expected to interact with Kmt6a, and possibly also with Kmt6b and Kmt6c. Repeated attempts to delete *SUZ12* (*FOXG_13918*) in *Fol* 4287 were unsuccessful, suggesting that this gene is essential in *F. oxysporum*. To generate loss-of-function strains, we replaced the native *SUZ12* promoter with the repressible *TET^OFF^* promoter [30]. Application of increasing concentrations of doxycycline to the *TET^OFF^::SUZ12* strains resulted in severely crippled growth (Fig. 3A), as well as in a complete loss of H3K27 methylation (Fig. 3B), thereby confirming the specificity of the anti-H3K27me3 antibody. The fact that repeated transfer of the mutant on doxycycline plates did not result in complete growth arrest (Supplementary Fig. S7E) could be due to a slight leakiness of the *TET^OFF^* promoter. We also generated *TET^OFF^::KMT6A* strains, whose phenotype was identical to that of the Δ*kmt6a* mutant, both in terms of vegetative growth and residual H3K27me3 levels (Figs 1D and E and 3A and B). By contrast, a mutant of the endophytic biocontrol strain *Fo* 47 carrying a *TET^OFF^::KMT6A* allele of the single *KMT6* homolog (*FOBCDRAFT_173144*) exhibited severely crippled growth on doxycycline media, similar to that of the *TET^OFF^::SUZ12* mutant of *Fol* 4287 (Supplementary Fig. S7D and E). This result, together with the clear differences between the loss-of-function phenotypes of *SUZ12* and *KMT6A* in *Fol* 4287, strongly suggests that the accessory genes *KMT6B/C* encode functional H3K27-specific methyltransferases that serve as PRC2 complex partners, similar to the core methyltransferase Kmt6a.

To conclusively demonstrate that *KMT6B/C* exhibit methyltransferase activity in the absence of *KMT6A*, we generated Δ*kmt6b/c/TET^OFF^::KMT6A* triple mutants. These mutants showed the same defects in colony growth and H3K27me3 levels in the presence of doxycycline as the *TET^OFF^::SUZ12* strain (Fig. 3C and D). Therefore, the H3K27-specific methyltransferase activity of Kmt6b/c in the Δ*kmt6a* mutant allows for more robust growth compared to the *SUZ12* loss-of-function strain.

Taken together, these findings establish that Ash1 and Kmt6a-c in *Fol* 4287 mediate H3K36 and H3K27 methylation, respectively, providing the first evidence, to the best of our knowledge, for the presence of functional histone modifiers encoded on accessory chromosomes.

### Facultative heterochromatin is activated in ∆*ash1* and ∆*kmt6a* mutants

**Figure 4.**
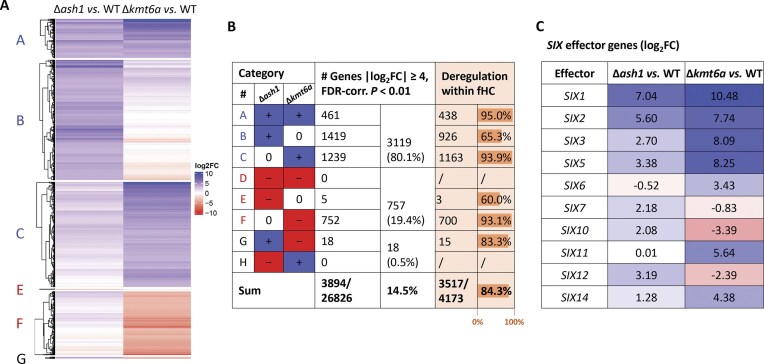
RNA-seq analysis in the Δ*ash1* and Δ*kmt6a* mutants reveals derepression of virulence-related effector genes under axenic conditions. The *Fol* 4287 WT and the Δ*ash1* and Δ*kmt6a* mutants were grown in biological quadruplicates for 2 days in liquid NO_3_ medium. (**A**) Genes significantly up- (log_2_-fold change ≥ 4, FDR-corrected *P* ≤ .01) or downregulated (log_2_-fold change ≤ −4, FDR-corrected *P* ≤ .01) in at least one of the deletion mutants compared to the WT are indicated in blue or red, respectively. Genes that are not DEGs (log_2_-fold change between −4 and 4) in one of the mutants are in white. Initially, eight categories (A–H) were extracted, and subsequently the genes were clustered for each category (no genes could be assigned to categories D and H). Genes missing in one of the mutants due to large-scale deletions (as determined by ChIP-seq; see Fig. 5A and B) were removed from the analysis of the respective strain (indicated in gray in the heatmap). (**B**) Numbers and percentages of deregulated genes within each category (white columns), or of deregulated genes associated with facultative heterochromatin (fHC; identified as marked with H3K27me3 in the ChIP-seq analysis; orange columns). 14.5% of all genes in *Fol* 4287 were included in this analysis. (**C**) Extracted log_2_-fold change (log_2_FC) differential gene expression of annotated *SIX* effector genes.

Because *Fol* 4287 Ash1 and Kmt6a contribute significantly to genome-wide H3K36 and H3K27 trimethylation levels, respectively (Fig. 1B and E), we hypothesized that they could repress genes located in facultative heterochromatin regions, including *in planta*-induced effector genes under axenic growth conditions. To test this idea, we performed genome-wide RNA- and ChIP-seq in the WT, ∆*ash1*, or ∆*kmt6a* deletion mutants grown in liquid minimal medium. RNA-seq was performed using four biological replicates per strain, and transcript levels of the two mutants were compared to those of the WT using stringent cutoff values (|log_2_FC| ≥ 4; FDR-corrected *P* ≤ .01), given the large number of deregulated genes (3894/26826 = 14.5%; annotation corrected for effector genes using *in planta* RNA-seq by [71]). Genes were grouped in categories based on significant deregulation in at least one strain in order to identify commonly or antagonistically deregulated genes. We found that 80.1% of the DEGs were upregulated in either one or both mutants (3119/3894; categories A–C) (Fig. 4A and B). Among the Gene Ontology (GO) molecular functions, *O*-methyltransferase activity (GO:0008171 in category B), monooxygenase activity (GO:0004497 in categories B and C), oxidoreductase activity (GO:0016705 in categories B and C), heme binding (GO:0020037 in categories B and C), iron ion binding (GO:0005506 in categories B and C), as well as transporter activities (GO:0042910 and GO:0015297 in category C) were enriched among the upregulated genes compared to the genomic background (Supplementary Table S2). In Δ*kmt6a*, a subset of genes was downregulated (770/3894 = 19.8%; categories F and G; Fig. 4A and B), whereas in Δ*ash1*, only 5 genes (0.1%) were downregulated (category E). Only 18 of the DEGs (0.5%) in the two mutants were regulated in opposite directions (category G). Importantly, 95.0% of the DEGs upregulated in both mutants (category A) are located in regions of facultative heterochromatin, as determined by their association with the H3K27me3 mark in the WT background (see ChIP-seq results below), while only 65.3% of the genes specifically upregulated in ∆*ash1* (category B) are associated with facultative heterochromatin, indicating a higher proportion of upregulated euchromatic genes (Fig. 4B). The 10 single-copy *SIX* effector genes located on pathogenicity chromosome 14 were all upregulated in at least one of the two mutants (log_2_FC ≥ 1), and 5 of them, including the two important virulence-related genes *SIX1* and *SIX3*, were upregulated both in ∆*ash1* and ∆*kmt6a* (Fig. 4C). Furthermore, around a third of the 532 predicted effector genes encoding small secreted proteins (161 genes; 30.3%) [71] were significantly upregulated (log_2_FC ≥ 4) in ∆*ash1* and/or ∆*kmt6a* (Supplementary Fig. S8B). Overall, these results establish a key role of Ash1 and Kmt6a in silencing *Fol* 4287 genes located in regions of facultative heterochromatin.

**Figure 5.**
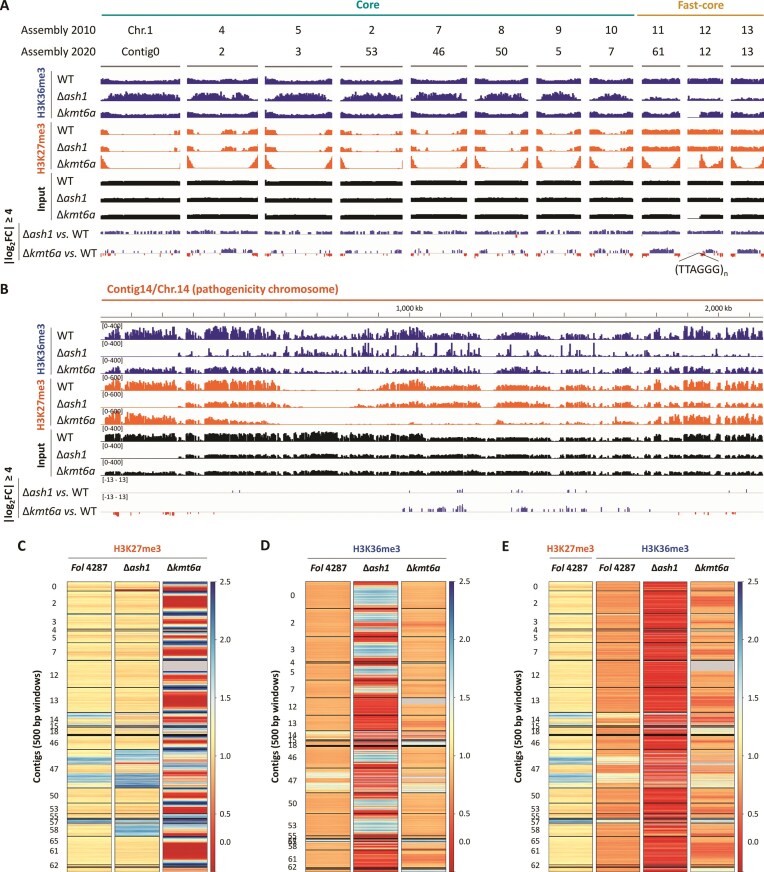
H3K36me3 and H3K27me3 distribution in Δ*ash1* and Δ*kmt6a* mutants reveals targeting of facultative heterochromatin by these two histone methyltransferases. The WT *Fol* 4287 and the Δ*ash1* and Δ*kmt6a* mutants were grown in biological duplicates for 2 days in liquid NO_3_ medium prior to ChIP-seq analysis using the indicated antibodies. H3K36me3 and H3K27me3 levels in the indicated strains on the eight core chromosomes (2010 genome assembly matching the contigs of the 2020 genome assembly) and the three fast-core chromosomes (**A**), or on the accessory chromosome 14 (**B**). Input samples were not treated with antibody. Note that scales in panels (A) and (B) are different, while track heights are the same. Tracks named |log_2_FC| ≥ 4 indicate the relative positions of DEGs in Δ*ash1* and Δ*kmt6a* versus WT as determined by RNA-seq (see Fig. 4). Levels of H3K27me3 within regions marked with H3K27me3 in the WT (**C**) or of H3K36me3 within regions marked with H3K36me3 in the WT (**D**) in the indicated strains. (**E**) Levels of H3K36me3 in the WT and the indicated mutants within regions marked with H3K27me3 in the WT. Gray areas indicate regions absent from the genome of a given strain due to deletion.

To investigate the possible link between gene expression and facultative heterochromatin, genome-wide ChIP-seq experiments with H3K36me3- and H3K27me3-specific antibodies were performed under the same conditions used for RNA-seq, and the WT, Δ*ash1*, and Δ*kmt6a* were grown in two biological replicates per strain (input samples were not treated with antibody). Reads were mapped against the 2020 PacBio genome assembly of *Fol* 4287 [70], where the large contigs mostly match the chromosomes reported in the 2010 Sanger assembly [5]. In Δ*ash1*, H3K36me3 was depleted in regions of facultative heterochromatin, including the accessory chromosome 14, the so-called “fast-core” chromosomes (core chromosomes 11–13 that are predominantly heterochromatic) [12], as well as the subtelomeric regions of the core chromosomes (Fig. 5A and B, blue track). FLAG-tagging of Ash1 protein followed by ChIP-qPCR confirmed that Ash1 is enriched at the promoter regions of the *SIX* effector genes located within facultative heterochromatin, but not at the euchromatic *EF1α* promoter (Supplementary Fig. S10A). In the ∆*kmt6a* mutant, the H3K27me3 mark was largely absent from the central chromosome regions, but still present in the subtelomeric regions, as evidenced in the pathogenicity chromosome 14 (Fig. 5B, orange track). This suggests that Kmt6a is mainly responsible for H3K27me3 in the central regions of fast-core and accessory chromosomes, whereas the subtelomeric regions can be methylated by Kmt6b/c.

Plotting the H3K27me3 levels in 500 bp windows for each contig [54] further confirmed the decrease of this epigenetic mark in the central chromosome regions of the ∆k*mt6a* mutant (Fig. 5C, in red) concomitant with an increase at the contig ends (in blue). Since no heterologous spike-in was applied to allow for absolute quantification and normalization of H3K27me3 levels, it cannot be ruled out that the observed increase at the chromosome ends in the ∆k*mt6a* mutant could be artefactual. Importantly, H3K27me3 was not affected in the ∆*ash1* mutant. The apparent increase of H3K27me3 in the accessory contigs 47 and 58 of ∆*ash1* (Fig. 5C) is an artifact caused by a duplication event, as evidenced by the two-fold read coverage of this region in the ∆*ash1* input sample compared to the WT (Supplementary Fig. S11A). Plotting the enrichment of H3K36me3 in the WT extracted both euchromatic core and facultative heterochromatic accessory regions (Fig. 5D), with the latter showing a specific loss of H3K36me3 in ∆*ash1* (in red). This effect was even more evident when facultative heterochromatin in the WT was extracted first by association with H3K27me3, thus confirming that Ash1 specifically targets these regions (Fig. 5E). Again, we observed only a limited impact on the other mark, and only a few genomic regions lost H3K36me3 in the ∆*kmt6a* mutant (e.g. contigs 2 and 13, which correspond to chromosomes 4 and 13, respectively; Fig. 5A and D). The apparent increase of H3K36me3 in contig 65 of ∆*kmt6a* is again due to a duplication event in this region (Supplementary Fig. S11B). Interestingly, this duplicated region encompasses *KMT6B* and *KMT6C*, resulting in the presence of two copies of each gene. Inspection of previous glycerol stocks of this mutant (∆*kmt6a* T11) and of an independently obtained ∆*kmt6a* mutant (T8) revealed that this duplication is not generally associated with *KMT6A* deletion (Supplementary Fig. S12A), but likely was selected over time in the ∆*kmt6a* T11 strain. Interestingly, comparative phenotyping of the ∆*kmt6a* T11 variant carrying the *KMT6B/C* duplication versus the original ∆*kmt6a* T11 mutant (∆*kmt6a* T11^original^) carrying only one copy of *KMT6B/C* revealed a trend for more robust growth, increased global and local H3K27me3 levels, and a lesser extent of *in vitro* upregulation of *SIX* effector genes (Supplementary Fig. S12B–G). This finding suggests an additive effect of the *KMT6B/C* gene copy number.

To evaluate how the observed loss of the H3K27me3 or H3K36me3 epigenetic marks in the different strains correlates with gene expression, peaks were called for the ChIP-seq samples using a fold change of 4, and associated with genes when the peak was detected within or up to 1 kb upstream of the gene body. Out of 1927 total affected genes, the two most represented categories were genes that had lost (1) H3K36me3 in ∆*ash1* (1522 genes) or (2) H3K27me3 in ∆*kmt6a* (347 genes, Supplementary Fig. S13A). Noteworthy, 67 genes had lost both H3K36me3 in ∆*ash1* and H3K27me3 in ∆*kmt6a*, and >90% of these are located in facultative heterochromatic regions, i.e. associated with H3K27me3 in the WT (Supplementary Fig. S13A). Comparing ChIP-seq and RNA-seq (|log_2_FC| ≥ 2), we detected a significant association of the following ChIP-seq groups and RNA-seq profiles (Supplementary Fig. S13B and C): (1) Genes that had lost H3K36me3 in ∆*ash1* were upregulated in this mutant (categories A and B in Fig. 4A and B); (2) genes that had lost H3K27me3 in ∆*kmt6a* were upregulated in this mutant (categories A and C in Fig. 4A and B). This result aligns with the previous observation that loss of H3K36me3 or H3K27me3 in facultative heterochromatic regions of ∆*ash1* or ∆*kmt6a*, respectively, results in gene activation. Regarding the genes downregulated in ∆*kmt6a*, their distribution across the genome reveals a trend for subtelomeric localization, in line with the putative increase of H3K27me3 in these regions (Fig. 5A and B).

Mapping of the ChIP-seq reads in the ∆*kmt6a* or ∆*ash1* mutants revealed the presence of deletions affecting the subtelomeric regions of chromosomes 12 or 14, respectively (Fig. 5A and B). While the breakpoint of the subtelomeric region of chromosome 14 lost in ∆*ash1* is in a repetitive region and could not be exactly located in the ChIP-seq analysis, the Illumina reads mapping to the deletion at the end of chromosome 12 in ∆*kmt6a* revealed up to 7 repeats of the telomeric sequence “TTAGGG” at the breakpoint (Fig. 5A), suggesting a *de novo* telomere formation and providing a possible explanation of how this shortened chromosome can be stably propagated. Analysis of the stock collection confirmed that the two originally obtained independent ∆*ash1* transformants T20 and T31 still contain an intact version of chromosome 14, indicating that the subtelomeric deletion occurred during later propagation of these transformants, but prior to preparation of the ChIP-seq samples. By contrast, in the ∆*kmt6a* strain T11 the used glycerol stock already lacked the subtelomere of chromosome 12, suggesting that this deletion had occurred early during generation and storage of this mutant (Supplementary Fig. S14). To ask whether loss of *KMT6A* increases the propensity for subtelomeric deletions in chromosome 12, an independent ∆*kmt6a* transformant (T8) with an intact chromosome 12 was subjected to 10 serial passages on complete medium (3 days of growth per passage). However, PCR analysis of the passaged lines failed to detect a loss of any subtelomeric region (Supplementary Fig. S14). Thus, the origin of the subtelomeric deletions in the mutants mentioned above remains elusive. Noteworthy, missing genes were omitted in the RNA-seq and ChIP-seq analyses, while duplicated regions were handled as normal in the RNA-seq due to the unknown effect of gene copy number and expression level. Because there were very few called peaks with increased methylation levels in the ChIP-seq, we considered these to be negligible (Supplementary Fig. S13A<?brk ?> and B).

In summary, a combination of expression and epigenetic profiling in *Fol* 4287 revealed that both Ash1-mediated H3K36me3 and Kmt6a-mediated H3K27me3 contribute to gene silencing within regions of facultative heterochromatin, with limited mutual impact between the two types of epigenetic mark.

### *KMT6B* functionally complements Δ*kmt6a* in *Fol* 4287 but not in *F. graminearum*

**Figure 6.**
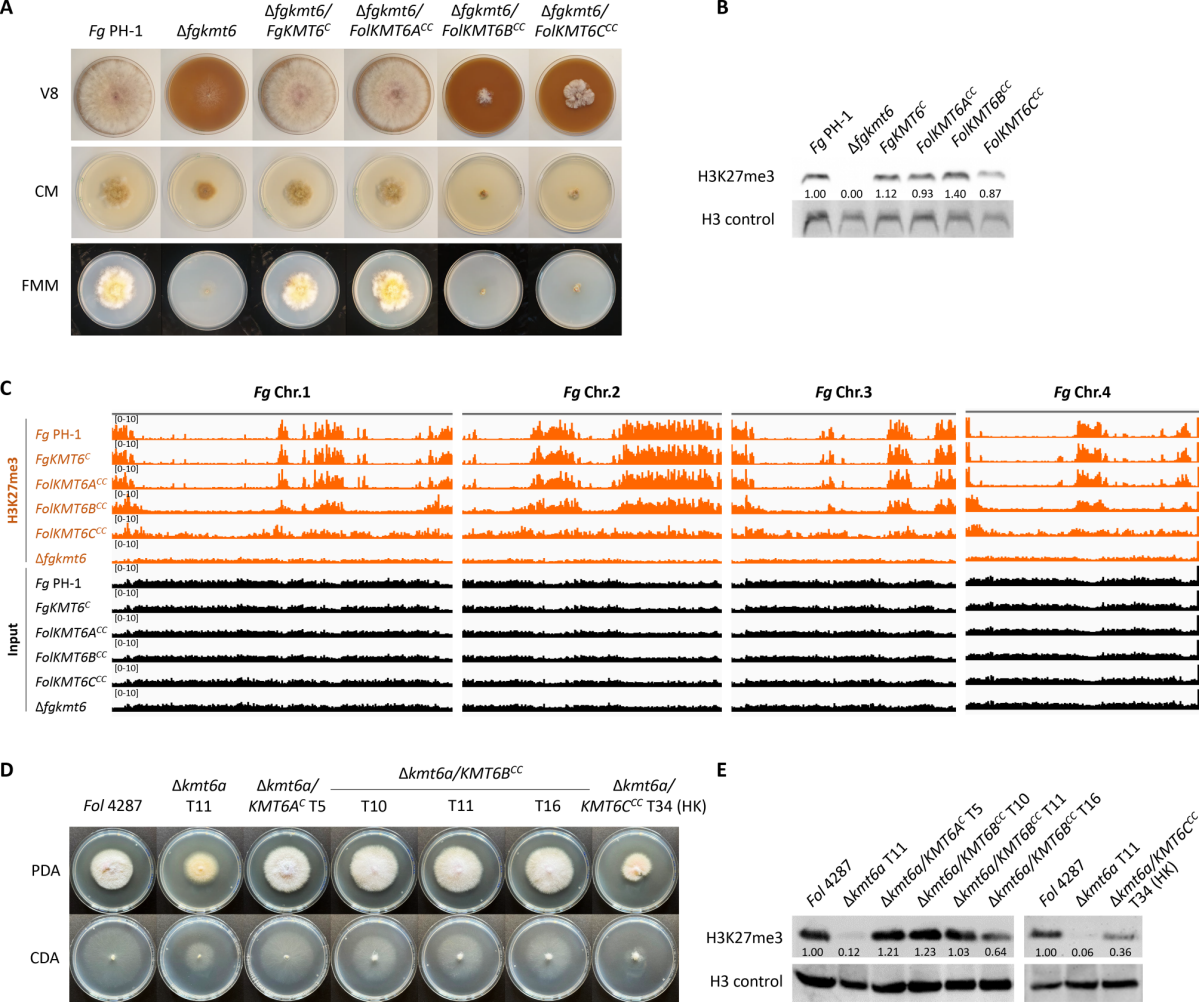
*KMT6B* functionally complements *KMT6A* in *Fol* 4287 but not in *F. graminarum*.(**A**) Colony phenotypes on complex (V8, CM) or minimal (FMM) media of the *F. graminearum* Δ*fgkmt6* mutant and transformants thereof complemented with the single native homolog *FgKMT6^C^* or with either of the three *Fol* 4287 homologs (*FolKMT6A^CC^, FolKMT6B^CC^, FolKMT6C^CC^*) (*n* = 3). (**B**) H3K27me3 levels in the Δ*fgkmt6* mutant were restored in all complemented transformants, as shown by western blot analysis with the specific antibody. Bands were quantified by densitometry and normalized to the H3 control. (**C**) The WT PH-1 and indicated mutants were grown for 3 days in liquid ICI supplemented with 60 mM glutamine prior to ChIP-seq analysis using the H3K27me3 antibody. Input samples were not treated with antibody. (**D**) *In locus* complementation of the *Fol* 4287 Δ*kmt6a* mutant with *KMT6B or KMT6C*. Quantification of colony diameters can be found in Supplementary Fig. S16A. Note that it was not possible to obtain a genetically stable strain expressing *KMT6C* from the *KMT6A* locus (HK: heterokaryon). (**E**) Genome-wide H3K27me3 was quantified by western blot for indicated complementation strains of *Fol* 4287, either grown in liquid culture (*KMT6B^CC^*) or on solid PDA (supplemented with the selection marker for *KMT6C^CC^*).

To further study the functions of Kmt6b and Kmt6c, we first performed complementation experiments with the different *Fol* 4287 *KMT6* genes in an *F. graminearum* mutant lacking the single *KMT6A* homolog *FGSG_15795* [9, 27]. *In locus* complementation of the mutant either with the native *FgKMT6* or the *Fol* 4287 *KMT6A* gene restored colony growth and overall H3K27 methylation levels (Fig. 6A and B). By contrast, complementation with *FolKMT6B* or *FolKMT6C* failed to restore colony growth but did restore overall H3K27 methylation levels. Unexpectedly, the strains complemented with *FolKMT6B* or *FolKMT6C* showed a more severe growth defect than the original Δ*fgkmt6* mutant, both on solid medium (Fig. 6A and B) and in liquid cultures (Supplementary Fig. S15A). ChIP-seq revealed a nearly WT-like H3K27me3 pattern in mutants expressing *FolKMT6B*, but altered genome-wide methylation for those expressing *FolKMT6C* (Fig. 6C). Furthermore, although overall H3K27 methylation levels were restored in *FolKMT6B* cross-complemented strains, transcripts of selected secondary metabolite genes (fusarin-biosynthetic gene *FUS1*, zearalenone-biosynthetic gene *ZEA2*, and fugralin-biosynthetic gene *PKS2* in Supplementary Fig. S15B and C [9, 75]) did not go down to WT levels, suggesting that H3K27me3 itself is not sufficient for gene repression at these loci.

Based on the finding that *KMT6B*/*C* restore H3K27 methylation levels at internal genomic regions of *F. graminearum* when expressed from the *FgKMT6* locus (Fig. 6C), but not from their native loci in *Fol* 4287 (Fig. 5A and B), we hypothesized that this discrepancy could be explained by the low expression level of *KMT6B/C* in *Fol* 4287 (Supplementary Fig. S9A). To test this, we performed *in locus* complementation of the Δ*kmt6a* mutant with *KMT6B* or *KMT6C*. While *KMT6B* expressed from the *KMT6A* locus restored colony growth, overall H3K27 methylation levels, and effector gene expression profiles, *KMT6C* brought back methylation but failed to restore WT growth (Fig. 6D and E and Supplementary Fig. S16). Furthermore, multiple attempts to obtain genetically stable *in locus KMT6C* complementation strains of the Δ*kmt6a* mutant were unsuccessful, with transformant colonies reverting back to the orange colony phenotype of the Δ*kmt6a* mutant after transfer to non-selective medium (Supplementary Fig. S16A), indicating that high expression levels of *KMT6C* could be deleterious in *Fol* 4287, as is the case in *F. graminearum*.

The observed difference of *KMT6B* and *KMT6C* in complementing the Δ*kmt6a* mutant phenotypes is intriguing considering that Kmt6b and Kmt6c are 96% identical at the amino acid level. Kmt6c carries a duplicated repetitive sequence at the C-terminus, which is lacking in Kmt6b, making it 15 amino acids longer. Modeling of the PRC2 complex in AlphaFold 3 [76] and subsequent alignment in Pymol (v3.0 Schrödinger, LLC), with Kmt6a, Kmt6b, or Kmt6c, revealed slight differences in their alignment with Suz12, while the alignment of Eed was conserved, both in the partial (Kmt6a/b/c-Suz12, Kmt6a/b/c-Eed) and complete complexes (Kmt6a/b/c-Suz12-Eed, Supplementary Fig. S17). Taken together, our results establish that the accessory homolog Kmt6b can functionally complement loss of the core methyltransferase Kmt6a in *Fol* 4287.

### Upregulation of *SIX* effector genes involves both heterochromatin removal and TF-mediated activation

**Figure 7.**
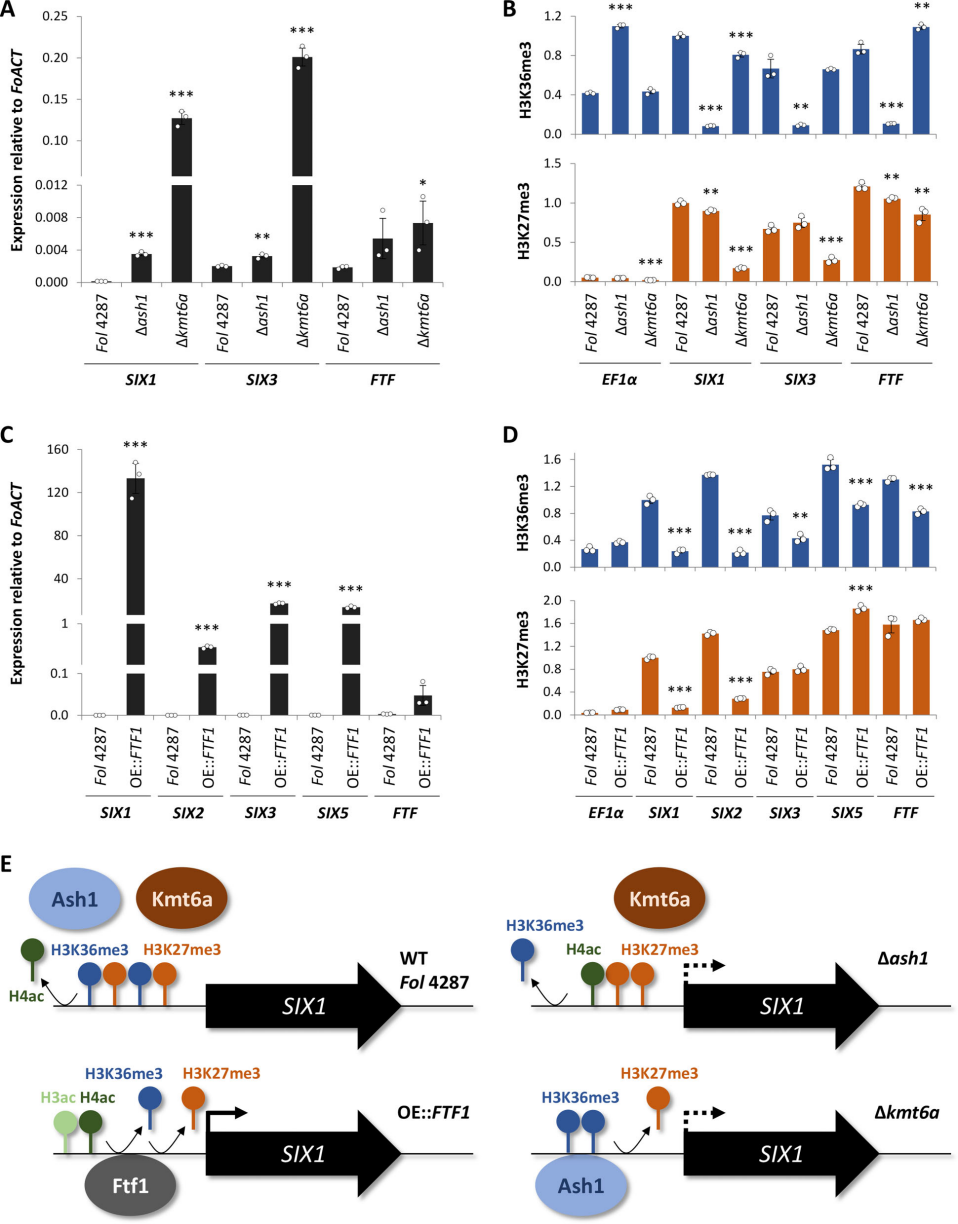
Transcriptional and epigenetic profiling of effector genes in the Δ*ash1*, Δ*kmt6a*, and OE::*FTF1* strains reveal full derepression upon *FTF1* overexpression. RT-qPCR expression analysis (**A**, **C**), and 5′ChIP analysis using antibodies against H3K36me3 (blue) or H3K27me3 (orange) (**B**, **D**) of the indicated strains grown for 2 days in liquid NO_3_ media (means ± SD, *n* = 3). The euchromatic housekeeping gene *EF1α* was used as a control. Transcript and methylation levels of each gene were compared between mutants and WT using *t*-test; *P* < .05 (*), *P* < .01 (**), *P* < .001 (***). (**E**) Schematic overview of the epigenetic and transcriptional regulation of *SIX1* by Ash1, Kmt6a, and Ftf1. Note that *FTF1* overexpression under axenic conditions results in full H3K36me3 and H3K27me3 demethylation and effector gene activation.

While epigenetic marks are known to be involved in the spatio-temporal regulation of fungal virulence genes, pathogenicity-related TFs also play a crucial role in this process. In *Fol* 4287, overexpression of the Zn(II)_2_Cys_6_-type TF *FTF1* (*FOXG_17458*), which is encoded on the accessory pathogenicity chromosome 14, results in a marked upregulation of the adjacent *SIX* effector genes under non-inducing axenic conditions [6]. Here we used ChIP coupled with qPCR to understand the interplay between TF- and chromatin-level control of *SIX* gene expression. In line with the RNA-seq data (see Fig. 4C), transcript levels of the virulence-related *SIX1* and *SIX3* genes during growth under non-inducing axenic conditions were significantly increased in ∆*ash1*, and even more so in ∆*kmt6a*, compared to the WT strain (Fig. 7A). Furthermore, in a transformant constitutively overexpressing the *FTF1* gene (OE::*FTF1*), transcript levels of *SIX1* and *SIX3* were increased by an additional three orders of magnitude compared to the ∆*kmt6a* mutant, reaching levels up to 120 times higher than those of the housekeeping gene actin (Fig. 7C). We noted that expression of the *FTF*-type TF genes (whose multiple homologs in *Fol* 4287 cannot be distinguished by RT-qPCR) was only slightly upregulated in the ∆*ash1* and ∆*kmt6a* mutants (Fig. 7A), likely because H3K27me3 was still present at the *FTF* gene promoters (Fig. 7B; for *FTF1*, see Supplementary Fig. S11C). This provides a possible explanation for the lack of full activation of the *SIX* genes in the ∆*ash1* and ∆*kmt6a* mutants, compared to the OE::*FTF1* strain. Interestingly, in the OE::*FTF1* strain the repressive histone marks H3K36me3 and H3K27me3 were strongly reduced at the *SIX1* and *SIX2* promoters (Fig. 7D), suggesting that full epigenetic derepression of *SIX* effector genes depends on TF-mediated gene activation. Noteworthy, in the OE::*FTF1* strain, H3K27me3 was still present at the bidirectional *SIX3* and *SIX5* promoter (Fig. 7D), despite the high transcript levels of these genes (Fig. 7C), suggesting that TF-mediated gene activation can occur even in the presence of H3K27me3.

A bromo-adjacent homology plant homeodomain-type H3K27me3-binding protein was previously implicated in Kmt6-mediated gene repression of *Fusarium* [74, 77], but little is known on how Ash1 promotes gene silencing. Using ChIP-qPCR with antibodies against H3- or H4-pan-acetylation (ac), we found an increase of H4 acetylation in the OE::*FTF1* strain, but particularly in the ∆*ash1* mutant, which showed slightly increased global and up to 20-fold increased local H4 acetylation levels (Supplementary Fig. S10B and C), likely as a result of reduced H3K36me3. In line with the latter hypothesis, both H3K36me3 and H4ac were unaffected in the ∆*kmt6a* mutant (Fig. 7B and Supplementary Fig. S10C). Specifically, H4K5/K8/K12ac, but not H4K16ac, was increased in ∆*ash1* (Supplementary Fig. S10D). These results suggest that H3K36me3-mediated H4 deacetylation contributes to gene silencing in facultative heterochromatin regions of *Fol* 4287.

ChIP-qPCR of the ∆*kmt6b/c* mutants did not detect significant changes in H3K27me3 levels at the promoters of the *SIX* genes or two subtelomeric genes located on chromosome 14 (Supplementary Fig. S9B). The latter two genes are in a region likely targeted by Kmt6b/c in the absence of Kmt6a. In line with this, expression of these two subtelomeric genes was not derepressed in the ∆*kmt6b/c* mutant (Supplementary Fig. S9A). Importantly, *SIX1* and *SIX3* expression levels were further upregulated in the ∆*kmt6b/c/TET^OFF^::KMT6A* triple mutant or the *TET^OFF^::SUZ12* H3K27me3-depletion strain, compared to the Δ*kmt6a* single mutant (Supplementary Fig. S9C). This additionally verifies that the accessory Kmt6b has the capacity to take over methylation and gene repression functions of the core Kmt6a methyltransferase.

Collectively, our findings demonstrate that removal of the repressive histone marks H3K36me3 and H3K27me3 contributes to derepression of *in planta*-induced effector genes, and combined with overexpression of the TF *FTF1*, results in full *SIX* gene activation even under non-inducing axenic conditions (Fig. 7E). Furthermore, our data indicate that H3K27me3 by itself is insufficient to enforce gene silencing, which likely requires additional downstream binding proteins, while H3K36me3 mediates gene silencing *via* histone deacetylation (Fig. 7E).

### The ∆*kmt6a* mutant is blocked at an early stage of root infection, resulting in complete loss of pathogenicity on tomato plants

**Figure 8.**
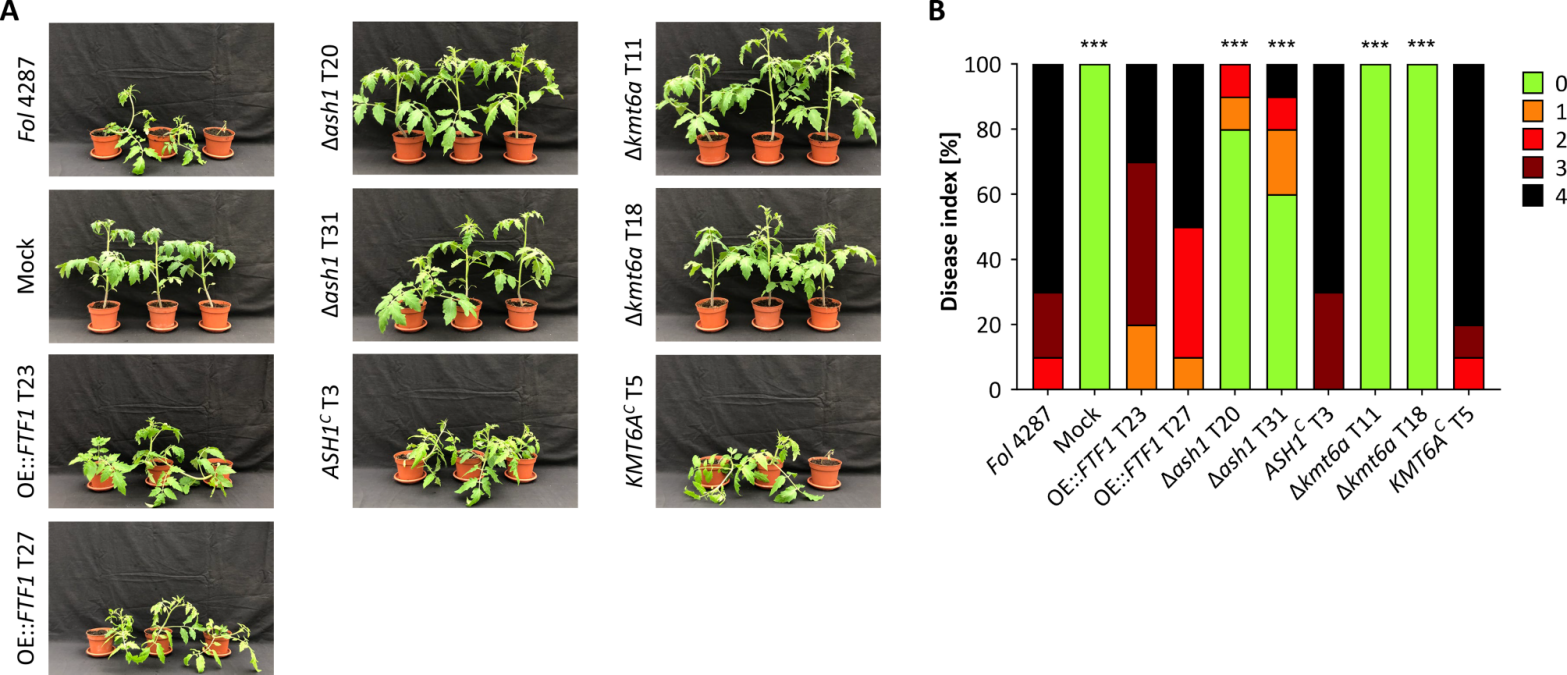
Virulence of the Δ*ash1* and Δ*kmt6a* mutants on tomato plants is strongly reduced or abolished, respectively. Roots of 10-day-old tomato seedlings (cultivar C32, *n* = 10) were inoculated with water (mock) or microconidia of indicated fungal strains, and severity of infection was assessed three weeks post inoculation. (**A**) Representative images of plants subjected to the indicated treatments. (**B**) Disease index was scored (0, no symptoms; 1, one brown vessel below the cotyledons; 2, one or two brown vascular bundles at cotyledons; 3, three brown vascular bundles and growth distortion; 4, all vascular bundles are brown, and the plant is either dead or very small and wilted). Kruskal-Wallis test was performed for statistical analysis relative to the WT *Fol* 4287; *P* < .001 (***).

**Figure 9.**
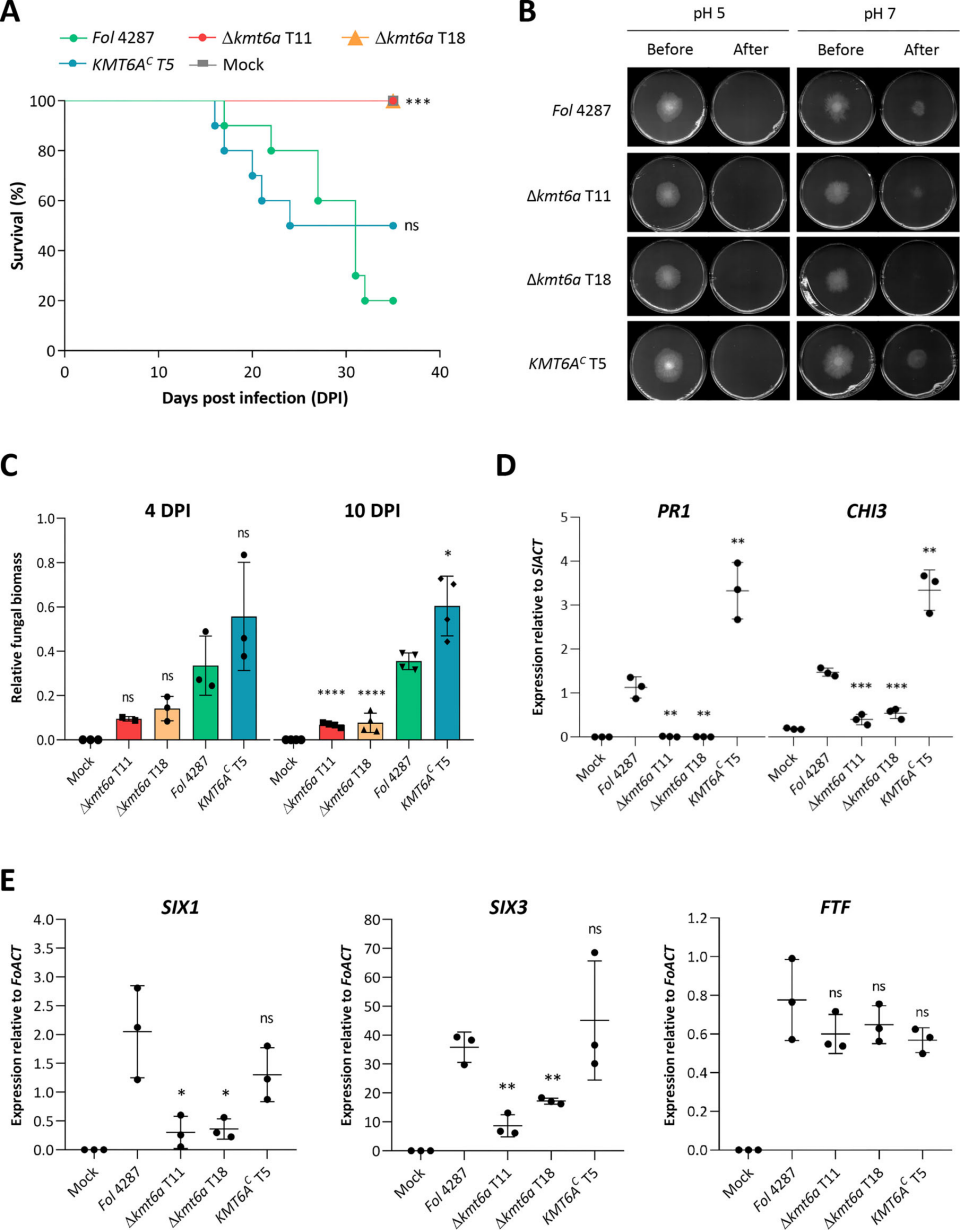
Kmt6a is required for invasive growth and pathogenicity on tomato plants. (**A**) Kaplan–Meier plot showing survival of tomato plants inoculated by dipping roots into a suspension of 5 × 10^6^ freshly obtained microconidia/ml of the indicated fungal strains. Percentage survival was recorded for 35 days. Three independent experiments with 10 plants per treatment were performed. Data shown are from one representative experiment. *P* < .001 (***); non-significant (ns); versus *Fol* 4287 according to log-rank test. (**B**) Invasive growth of the indicated strains was determined by spot-inoculating 5 × 10^4^ microconidia on a cellophane membrane placed on minimal medium plates buffered at pH 5 or pH 7. Plates were scanned after 4 days of incubation at 28°C (before). The cellophane with the fungal colony was removed and plates were incubated for an additional day to visualize mycelial growth indicative of cellophane penetration (after). Images shown are representative of three biological replicates. (**C**) Relative amount of fungal DNA in roots of tomato plants at 4 and 10 DPI with the indicated fungal strains was measured by qPCR of the *Fol* 4287-specific *SIX1* gene and normalized to the tomato *GAPDH* gene. Data shown represent the mean and standard deviations of three (4 DPI) or four (10 DPI) independent biological replicates. *P* < .05 (*); *P* < .0001 (****); non-significant (ns) versus *Fol* 4287 according to unpaired *t*-test. Transcript levels of tomato defense genes *PR1* and *CHI3* (**D**) or of the fungal virulence-associated genes *SIX1, SIX3*, or *FTF* (**E**) were measured by RT-qPCR in cDNA samples obtained from infected tomato roots at 6 DPI. Relative transcript levels are normalized to the tomato (D) or the fungal (E) actin gene. Data shown represent the mean and standard deviation of three independent biological replicates. *P* < .05 (*); *P *< .01 (**); *P* < .001 (***); non-significant (ns) versus *Fol* 4287 according to unpaired *t*-test.

To determine the role of Ash1 and Kmt6a in pathogenicity of *Fol* 4287 on tomato plants, root infection assays were conducted with the ∆*ash1* and ∆*kmt6a* mutants and the respective complemented strains. Loss of *ASH1* or *KMT6A* resulted in a significant reduction or complete loss of virulence, respectively, considering different parameters such as reduction in plant fresh weight, disease index (Fig. 8), and plant mortality (Fig. 9A). Loss of pathogenicity was detected in three independent ∆*kmt6a* mutants and was independent of the presence/absence of the subtelomeric region of chromosome 12, or of the presence of 2 or 4 copies of the *KMT6B/C* genes (Supplementary Fig. S18). By contrast, the double deletion mutant lacking the *KMT6B/C* genes was fully virulent (Supplementary Fig. S19A). Thus, Kmt6a, but not Kmt6b/c, is essential for pathogenicity of *Fol* 4287 on tomato plants, in spite of the fact that *KMT6B/C* were upregulated in the ∆*kmt6a* mutant, both under axenic (Supplementary Fig. S9A) and *in planta* conditions (Supplementary Fig. S19C). Similar to the ∆*kmt6a* mutant, the *FTF1* overexpression strain exhibited reduced colony growth (Supplementary Fig. S20A) and high *in vitro* transcript levels of the *SIX* effector genes (Fig. 7C), yet this strain caused disease on tomato plants as efficiently as the WT strain (Fig. 8 and Supplementary Fig. S19A). This suggests that loss of pathogenicity in ∆*kmt6a* is due to effects unrelated to these phenotypes.

To further dissect the role of *KMT6A* during plant infection, we conducted a comprehensive phenotypic analysis of the Δ*kmt6a* mutant. Compared to the WT, the ∆*kmt6a* mutants showed increased sensitivity to the cell wall-targeting compounds calcofluor white and Congo red and to the membrane-damaging agent sodium dodecyl sulfate, while sensitivity to hyperosmotic stress was unaffected (Supplementary Fig. S21). Furthermore, invasive growth across cellophane membranes was reduced in the ∆*kmt6a* mutants (Fig. 9B). Because cell wall integrity and invasive growth are important virulence functions in *F. oxysporum*, the observed phenotypes might at least partially account for the virulence defect of the ∆*kmt6a* mutant.

We next asked at which stage infection of the ∆*kmt6a* mutant is blocked. Quantification of the fungal biomass in infected tomato roots revealed a reduced fungal burden of ∆*kmt6a* compared to the WT or the complemented strain, with a significant difference at 10 DPI (Fig. 9C). Moreover, expression analysis using RNA from infected tomato roots at 6 DPI revealed that activation of the tomato defense genes *PR1* (pathogenesis-related protein 1) and *CHI3* (chitinase 3) was markedly reduced in plants inoculated with ∆*kmt6a* compared to those inoculated with the WT or complemented strains (Fig. 9D). This is likely a consequence of the lower fungal burden of the mutant and indicates that the infection block in ∆*kmt6a* is not caused by hyperactivation of the plant immune response. In line with this, the expression of the virulence effector genes *SIX1* and *SIX3* at 6 DPI was significantly reduced in ∆*kmt6a* (Fig. 9E), further suggesting that this mutant is blocked at an early stage of root infection before reaching the xylem, where *SIX* genes are fully activated [68]. Corroborating this hypothesis, the early root colonization (ERC) effector gene *ERC1*, which is located on a core chromosome and encodes a secreted putative cellulose-binding protein [68], was expressed both at early and later time points of infection in ∆*kmt6a*, whereas expression in the WT strain was high at 2 DPI but plummeted at 6 DPI (Supplementary Fig. S22A), as described previously [68]. We also noted that *FOXG_00543* and *FOXG_11153*, two *in planta*-upregulated core genes [78] located in euchromatic regions with H3K4 methylation [12], were not differentially expressed in WT and ∆*kmt6a*, either *in planta* or under axenic conditions (Supplementary Fig. S22B).

To further corroborate these findings, *in planta* RNA-seq of Δ*kmt6a* versus WT at 6 DPI was performed using three biological replicates per strain. GO-enriched molecular functions of deregulated tomato genes essentially revealed the absence of a plant response to Δ*kmt6a*. For example, chitinase activity (GO:0004568), peroxidase activity (GO:0004601), and defense response to fungus (GO:0050832) were downregulated in plants inoculated with Δ*kmt6a* compared to the WT (Supplementary Table S3). Among the fungal genes, carbohydrate metabolic process (GO:0005975) and several GO terms connected to the proteasome complex (GO:0005839, GO:0019773) and chaperone activity (GO:0140662) were specifically downregulated in Δ*kmt6a* versus WT during infection, but not during axenic conditions (Supplementary Table S3). When filtering for euchromatic versus facultative heterochromatic genes specifically upregulated *in planta* (*in planta* WT versus axenic WT), we found that *KMT6A* deletion primarily affects *in planta* expression of heterochromatic genes (Supplementary Fig. S22C). Ca. 50% of *in planta*-upregulated heterochromatic genes were deregulated in Δ*kmt6a*, compared to only 15% of *in planta*-expressed euchromatic genes (Supplementary Fig. S22C). Interestingly, 85% of the heterochromatic genes upregulated in Δ*kmt6a in planta* were also upregulated in Δ*kmt6a* under axenic conditions (Supplementary Fig. S22C). These results suggest the presence of a Kmt6-independent mechanism for *in planta*-activated core genes.

*In planta*-induced expression of *SIX* effector genes was previously shown to be mediated by the *FTF* family of TFs [6]. Here we found that, in contrast to the *SIX* genes, *in planta* transcript levels of the *FTF* genes at 6 DPI were similar in ∆*kmt6a* and WT (Fig. 9E). This indicates that *FTF* expression is necessary but not sufficient for *in planta* activation of *SIX* effector genes. To further test this idea, we constitutively overexpressed *FTF1* in the ∆*kmt6a* deletion background. The ∆*kmt6a*/OE::*FTF1* strains showed marked upregulation of *SIX1* and *SIX3*, both under axenic (Supplementary Fig. S20C) and *in planta* conditions (Supplementary Fig. S19B). Moreover, while the H3K27me3 mark at the *SIX3* promoter was still present in the WT/OE::*FTF1* strain (Fig. 7D and Supplementary Fig. S20D), it was absent in ∆*kmt6a*/OE::*FTF1*, resulting in full *SIX3* derepression (Supplementary Fig. S20C and D). However, overexpression of *FTF1* failed to restore the virulence defect of the ∆*kmt6a* mutant (Supplementary Fig. S19A). This finding reveals that loss of pathogenicity in the ∆*kmt6a* mutant is due to additional Kmt6a-dependent virulence factors, which—unlike the *SIX* effector genes—are not directly controlled by Ftf1.

Besides being devastating plant pathogens, members of the FOSC have been reported to cause opportunistic human infections [2]. Previous work established that the tomato-infecting isolate *Fol* 4287 can act as a cross-kingdom pathogen, causing disseminated infections in immunosuppressed mice [79] and killing larvae of the invertebrate insect model host *G. mellonella* [69]. Interestingly, we found that Kmt6a was dispensable for pathogenicity of *Fol* 4287 on *G. mellonella* (Supplementary Fig. S23). This suggests that H3K27me3 is not associated with infection of *F. oxysporum* on animal hosts, a process that appears to be governed mainly by core genes.

Taken together, our results demonstrate that *KMT6A*, but not *KMT6B/C*, is essential for pathogenicity of *F. oxysporum* on plant hosts, and that the ∆*kmt6a* mutant is blocked at an early stage of root infection. Furthermore, our data establish that activation of *SIX* effector genes requires the removal of repressive H3K27me3 and H3K36me3 histone marks, which can be accomplished under axenic conditions by *KMT6A* and *ASH1* deletion or by overexpression of the TF *FTF1*. Our work unravels a complex regulatory network involving a combination of chromatin-level and TF-mediated regulation of accessory virulence effector genes.

## Discussion

In this study, we characterized two histone methyltransferases targeting facultative heterochromatin in the economically important phytopathogen *F. oxysporum*. This chromatin state is of particular interest, since it controls the expression of virulence-associated genes [7, 8]. These include the well-characterized *SIX* effectors, which are necessary and sufficient for virulence on tomato plants as demonstrated by horizontal transfer of the pathogenicity chromosome 14 to the non-pathogenic *F. oxysporum* isolate *Fo* 47, conferring virulence on tomato [5]. We used a combination of RNA- and ChIP-seq to show that the H3K36-specific Ash1 and the H3K27-specific Kmt6a methyltransferases both contribute to silencing of genes located in facultative heterochromatic regions under non-inducing conditions. While deletion of *ASH1* had almost exclusively an activating effect on gene expression, partially within euchromatic regions, *KMT6A* deletion also resulted in reduced expression of subtelomeric genes possibly caused by a local increase of H3K27me3 (ca. 20% of the DEGs). Noteworthy, in some instances the presence of H3K27me3 appeared to be insufficient for gene silencing. This was the case in the *F. graminearum* cross-complementation experiment, further discussed below, as well as the *FTF1* overexpression strain, which showed high expression of *SIX3* in spite of the presence of H3K27me3 at its promoter. Similar results were recently reported for *N. crassa* and *F. graminearum*, where H3K27me3 was insufficient for gene silencing without its reader protein EPR-1/BP1 [74, 77] or deacetylation at H3K14 by *N. crassa* RPD3L [80]. While we only found a negligible increase of H3ac in Δ*kmt6a* and no increase at the *SIX3* promoter in OE::*FTF1* (Supplementary Fig. S10C), we did not explore further the epigenetic profiles at the genes that lost H3K27me3 in Δ*kmt6a* but remained silent (Supplementary Fig. S13). The possible role of H3 deacetylation in facultative heterochromatin of *F. oxysporum* remains to be elucidated.

How Ash1 facilitates gene silencing is still under investigation. Our data indicate that Ash1-mediated H3K36me3 results in histone deacetylation, since H4ac was specifically increased in the ∆*ash1* and OE::*FTF1* strains, while H3K36me3 was reduced. Our data suggest that deacetylation of H4 acts downstream of Ash1-mediated H3K36 methylation. H3K36 methylation and histone deacetylation could be linked by the chromodomain-containing protein Eaf3, which was recently suggested as complex partner of Ash1 in *Magnaporthe oryzae* [81] and *Zymoseptoria tritici* [54]. In a very elegant study in *M. oryzae*, Eaf3 was shown to bind both H3K36me2 and H3K27me3, and to subsequently promote gene silencing by deacetylation of H3 and H4 as well as increased nucleosome occupancy [81]. Our results do not provide evidence for mutual impact between the two facultative heterochromatic marks H3K27me3 and H3K36me3 in *Fol* 4287, since H3K27me3 was not affected in ∆*ash1* or *vice versa*. The same result was reported in a genome-wide study of the ∆*ash1* mutant of *Z. tritici* [54], whereas in *N. crassa* and *F. graminearum*, loss of *ASH1* resulted either in loss or gain of H3K27me3 [18, 82]. Furthermore, alterations in the H3K36me3 profile were recently reported for the ∆*kmt6* mutant of *F. proliferatum* [16].

Here, we demonstrate that histone modifiers encoded on an accessory chromosome are functional and can contribute to the genome-wide epigenetic profile. Upon deletion of the core homolog *KMT6A*, we detected residual H3K27me3, which was absent in the triple *KMT6A-C* loss-of-function strain. In line with this, no H3K27me3 was detected in a loss-of-function mutant of *SUZ12*, encoding a unique PRC2 complex partner. We verified that *KMT6B* can complement the Δ*kmt6a* mutant when expressed at sufficient strength from the *KMT6A* promoter. In contrast, *KMT6B* did not functionally complement loss of *KMT6* in *F. graminearum*. In addition, H3K27me3 deposited by *KMT6C* in both fungal species did not restore the growth defect but resulted in an even more severe phenotype. Based on these results, we conclude that the accessory methyltransferases Kmt6b and Kmt6c can interact with Suz12 and methylate both in *Fol* 4287 and in *F. graminearum*, whereas phenotypic complementation appears to be species-specific. It is tempting to speculate that interaction with upstream/downstream factors determines both the deposition of H3K27me3 and subsequent facilitation of gene silencing.

ChIP-seq of the ∆*kmt6a* mutant showed that Kmt6b maintains H3K27me3 in the subtelomeric regions of the chromosomes but fails to fully methylate the central chromosome parts at the given expression level. Nonetheless, Δ*kmt6a* mutants showed far more robust growth than *SUZ12* loss-of-function strains, suggesting the possibility of an adaptive advantage of carrying multiple *KMT6* homologs. Further supporting this notion, a spontaneous duplication of *KMT6B/C* giving rise to 4 copies quickly became fixed in independent Δ*kmt6a* mutants. While we detected slightly increased H3K27me3 levels in these strains, the effect on growth and effector gene expression was very minor, and no impact on virulence was detected. Therefore, the presence of 2 versus 4 accessory copies of *KMT6B/C* does not affect the overall conclusions. The fact that Kmt6b/c are encoded on an accessory chromosome opens the possibility that these methyltransferases could be horizontally transferred and may play a role in shaping the facultative heterochromatin of the accessory genome. An intriguing hypothesis is that accessory chromosomes might act as selfish elements that encode their own regulatory elements, including TFs or histone modifiers. Phylogenetic analysis revealed the presence of additional Kmt6 homologs in other members of the FOSC and FSSC, both of which are known to harbor accessory chromosomes linked to pathogenicity [83]. Recently, horizontally transferred accessory chromosomes in the insect pathogen *Metarhizium* were reported to encode homologs of histone H3, as well as a histone methyltransferase and a histone acetyltransferase. Habig and colleagues suggested that accessory chromosome-encoded histone modifiers might play a role in the chromosome transfer process itself [84]. Indeed, H3K27me3 has been linked to the stability of accessory chromosomes in *Z. tritici* [11], while its role in chromosome transfer remains to be elucidated. In *Z. tritici*, increased stability of the genome and of accessory chromosomes was observed upon loss of H3K27me3 in the ∆*kmt6* mutant [11]. By contrast, in *Fol* 4287 we detected independent subtelomeric deletions and large-scale duplications in the ∆*kmt6a* and ∆*ash1* mutants. Particularly chromosome 12 appears to act as a hotspot for subtelomere loss, since independent ∆*kmt6a* mutants lost subtelomeric fragments of different lengths, while the regions closer to the centromere were stable (Supplementary Fig. S20E). Further studies are required to define the environmental conditions promoting these structural alterations. In our hands, serial passaging of the ∆*kmt6a* mutant on complete medium did not result in subtelomeric deletions. In contrast, a similar passaging experiment with an *F. fujikuroi* ∆*ash1* mutant resulted in progressive loss of subtelomeric regions as well as of its only accessory chromosome [17].

Importantly, we observed a strong impact of facultative heterochromatin on virulence of *Fol* 4287. While the ∆*ash1* mutant displayed reduced virulence on tomato, the ∆*kmt6a* mutants were completely non-pathogenic. Loss of pathogenicity in ∆*kmt6a* was caused by a block in the early stages of root infection, as shown by a drastic reduction of fungal burden and of expression of the xylem-induced effectors *SIX1* and *SIX3*, as well as by inappropriately prolonged expression of the early stage root colonization effector *ERC1* [68]. Interestingly, *KMT6* deletion in two phytopathogens, *Ustilaginoidea virens* and *M. oryzae*, also resulted in reduced virulence on rice plants [85, 86], and moreover, knockout of each of the PRC2 components in *F. graminearum* resulted in loss of virulence on wheat [74]. Although the exact reason for the loss of pathogenicity is currently unknown, we found that the ∆*kmt6a* mutants displayed defects in cell wall integrity and invasive growth, two important virulence functions of *Fol* 4287 on the plant host [58], [87]. Additionally, *in planta* RNA-seq revealed specific downregulation of proteasome function and chaperone activity in Δ*kmt6a* compared to the WT [88]. While this provides a possible explanation for the impaired infection, further studies are required to identify the genes controlled by Kmt6a that are responsible for the loss of pathogenicity in the deletion mutant.

A key finding of our study was that the constitutive overexpression of the TF *FTF1* was sufficient to obtain massive upregulation of *SIX* effector genes under non-inducing axenic conditions. This was in line with increased histone acetylation and demethylation of the repressive histone marks at H3K27 and H3K36 observed in this strain (Fig. 7E). How this epigenetic shift takes place and which downstream factors are recruited to induce acetylation and demethylation will be of great interest, particularly since the identity of the fungal H3K27-specific demethylase remains unclear. Ftf1 seems to function as a sort of “pioneer” TF, which can bind a DNA sequence in previously repressed chromatin [89]. Interestingly, although *FTF1* overexpression restored *in planta SIX* gene expression in the ∆*kmt6a* background, it failed to rescue pathogenicity. This provides strong evidence for the presence of additional Kmt6a-dependent virulence factors other than the SIX effectors or the Ftf TFs that are essential for plant infection by *F. oxysporum*. Recently, a different type of cross-talk between the Zn(II)_2_Cys_6_-type TF LmPf2 and the repressive histone modification H3K9me produced by Kmt1 in chromatin-level effector gene regulation was reported in the oilseed rape pathogen *Leptosphaeria maculans* [90]. Interestingly, their findings differed from the cross-talk between Ftf1 and Kmt6-mediated H3K27me3 observed in the present study, as overexpression of *LmPf2* was unable to overrule the inhibitory effect of Kmt1 and result in derepression under axenic conditions [90].

In summary, to the best of our knowledge, our work demonstrates for the first time the cooperative action of multiple H3K27-specific methyltransferases in a filamentous fungus in controlling gene expression within regions of facultative heterochromatin together with the H3K36-specific histone methyltransferase Ash1. The combinatorial control of TF- and chromatin-mediated effector gene regulation observed in *F. oxysporum* could provide a general model to unravel the elusive mechanisms governing *in planta* activation of virulence mechanisms in phytopathogenic fungi.

## Supplementary Material

gkaf1441_Supplemental_File

## Data Availability

All RNA- and ChIP-seq data are available at GEO under the accession number GSE253117.

## Notes

Present address: State agency for social services (LAsD), Department of Environmental health protection, Neumünster 24534, Germany
