# Supplementary material for: Facultative heterochromatin mediated by core and accessory chromosome-encoded H3K27-specific methyltransferases controls virulence in a fungal phytopathogen
Source: Nucleic Acids Res. 2026 Jan 6;54(1):gkaf1441. doi: 10.1093/nar/gkaf1441 (PMC12774646; doi:10.1093/nar/gkaf1441)
Supplement: gkaf1441_Supplemental_File [file gkaf1441_supplemental_file.pdf]

Supplementary Data

Facultative heterochromatin mediated by core and accessory chromosome-encoded H3K27-specific methyltransferases controls virulence in a fungal phytopathogen

Slavica Janevska[1,2,†,*], Lucía Gómez-Gil[3,†], Tongta Sae-Ong[4], Umer Farooq[1], Lena Studt-Reinhold[5,#], Manuel Sánchez López-Berges[3], Gianni Panagiotou[4,6], Martijn Rep[2], Antonio Di Pietro[3]

[1](Epi-)Genetic Regulation of Fungal Virulence, Leibniz Institute for Natural Product Research and Infection Biology – Hans Knöll Institute (Leibniz-HKI), 07745 Jena, Germany

[2]Molecular Plant Pathology, University of Amsterdam, 1098 XH Amsterdam, Netherlands

[3]Department of Genetics, University of Córdoba, 14014 Córdoba, Spain

[4]Microbiome Dynamics, Leibniz Institute for Natural Product Research and Infection Biology – Hans Knöll Institute (Leibniz-HKI), 07745 Jena, Germany

[5]Applied Genetics and Cell Biology, University of Natural Resources and Life Sciences, Vienna (BOKU), Campus Tulln, 3430 Tulln an der Donau, Austria

[6]Friedrich Schiller University Jena, Faculty of Biological Sciences, 07743 Jena, Germany

[#]Current address: State agency for social services (LAsD), Department of Environmental health protection, 24534 Neumünster, Germany

[†]These authors contributed equally.

*Correspondence: slavica.janevska@leibniz-hki.de

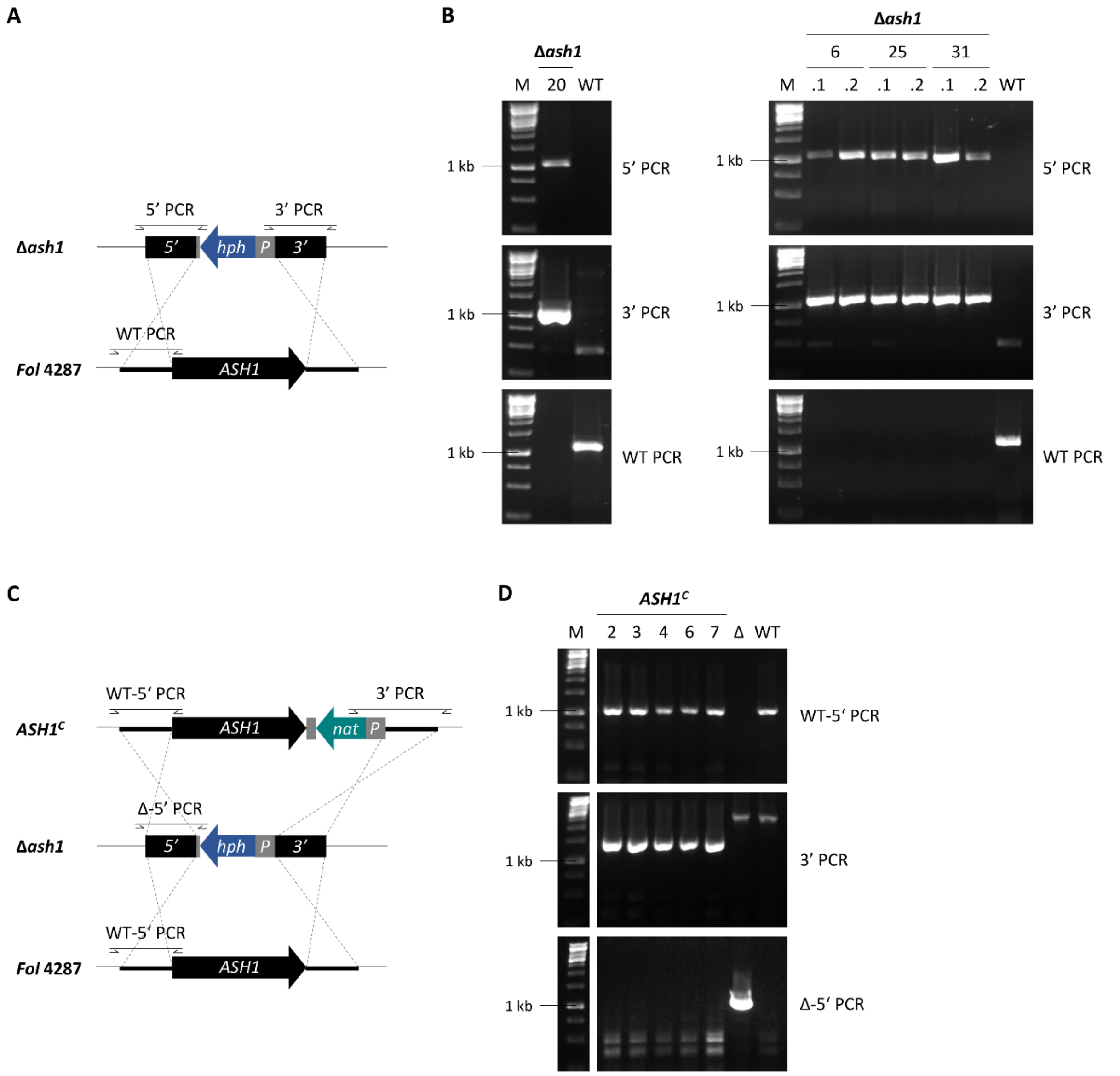

Figure S1: Verification of Δ*ash1* deletion and *ASH1*^{C} complemented mutants. A,B) Deletion of the *ASH1* gene *via* homologous recombination and replacement with the hygromycin B resistance cassette (*hphR*) (A) was verified by amplification of 5′ (Ash1_5diag/trpC_T) and 3′ (Ash1_3diag/trpC_P2) flanks in 4 independent transformants, while no amplification was detected for the wild-type PCR (WT; Ash1_5diag/Ash1_R) (B). **C,D)** *In locus* integration of the complementation construct using the nourseothricin resistance cassette (*natR*) (C) was verified for 5 independent *ASH1*^{C} transformants using primers Ash1_5diag/Ash1_R (WT-5′), Ash1_3diag/nat1_seq_R (3′) and Ash1_5diag/trpC_T (Δ-5′, untransformed nuclei) (D).

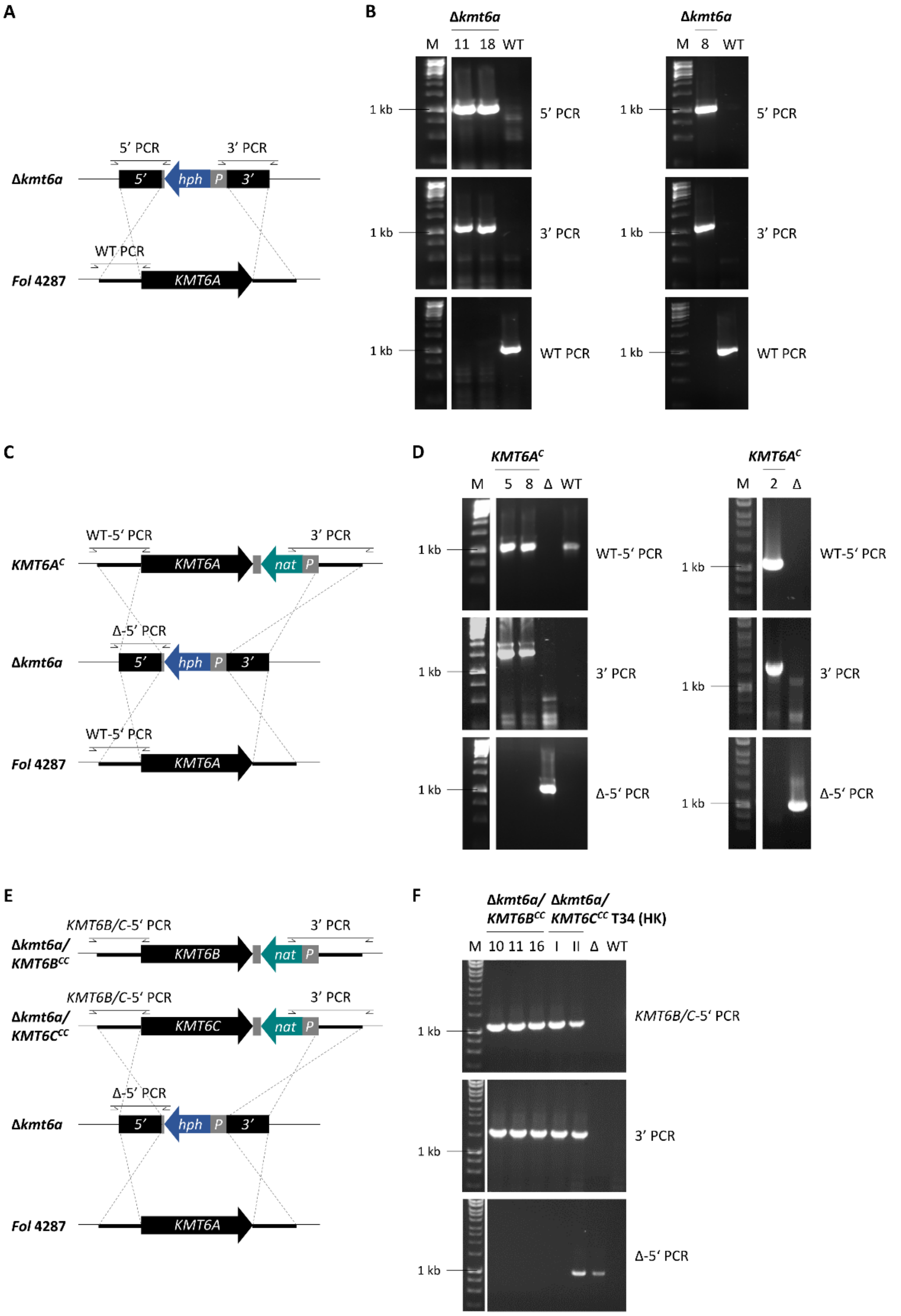

Figure S2: Verification of Δ*kmt6a* deletion, *KMT6A*^{C} complemented and *KMT6B/C*^{CC} cross-complemented mutants of *Fol* 4287. A,B) Deletion of the *KMT6A* gene *via* homologous recombination and replacement with the hygromycin B resistance cassette (*hphR*) (A) was verified by amplification of 5′ (Kmt6a_5diag/trpC_T) and 3′ (Kmt6a_3diag/trpC_P2) flanks in 3 independent transformants, while no amplification was detected for the wild-type PCR (WT; Kmt6a_5diag/Kmt6a_R) (B). **C,D)** *In locus* integration of the complementation construct using the nourseothricin resistance cassette (*natR*) (C) was verified for 3 independent *KMT6A*^{C} transformants using primers Kmt6a_5diag/Kmt6a_R (WT-5′), Kmt6a_3diag/nat1_seq_R (3′) and Kmt6a_5diag/trpC_T (Δ-5′, untransformed nuclei) (D). **E,F)** *In locus* integration of the cross-complementation constructs using *natR* (E) was verified for 3 independent *KMT6B*^{CC} transformants and one heterokaryotic (HK) transformant of *KMT6C*^{CC} using primers Kmt6a_5diag/Kmt6b-c_R (*KMT6B/C*-5′), Kmt6a_3diag/nat1_seq_R (3′) and Kmt6a_5diag/trpC_T (Δ-5′, untransformed nuclei) (F). I and II refer to two gDNA extractions where *KMT6C*^{CC} first seemed pure, but turned out to be a heterokaryon.

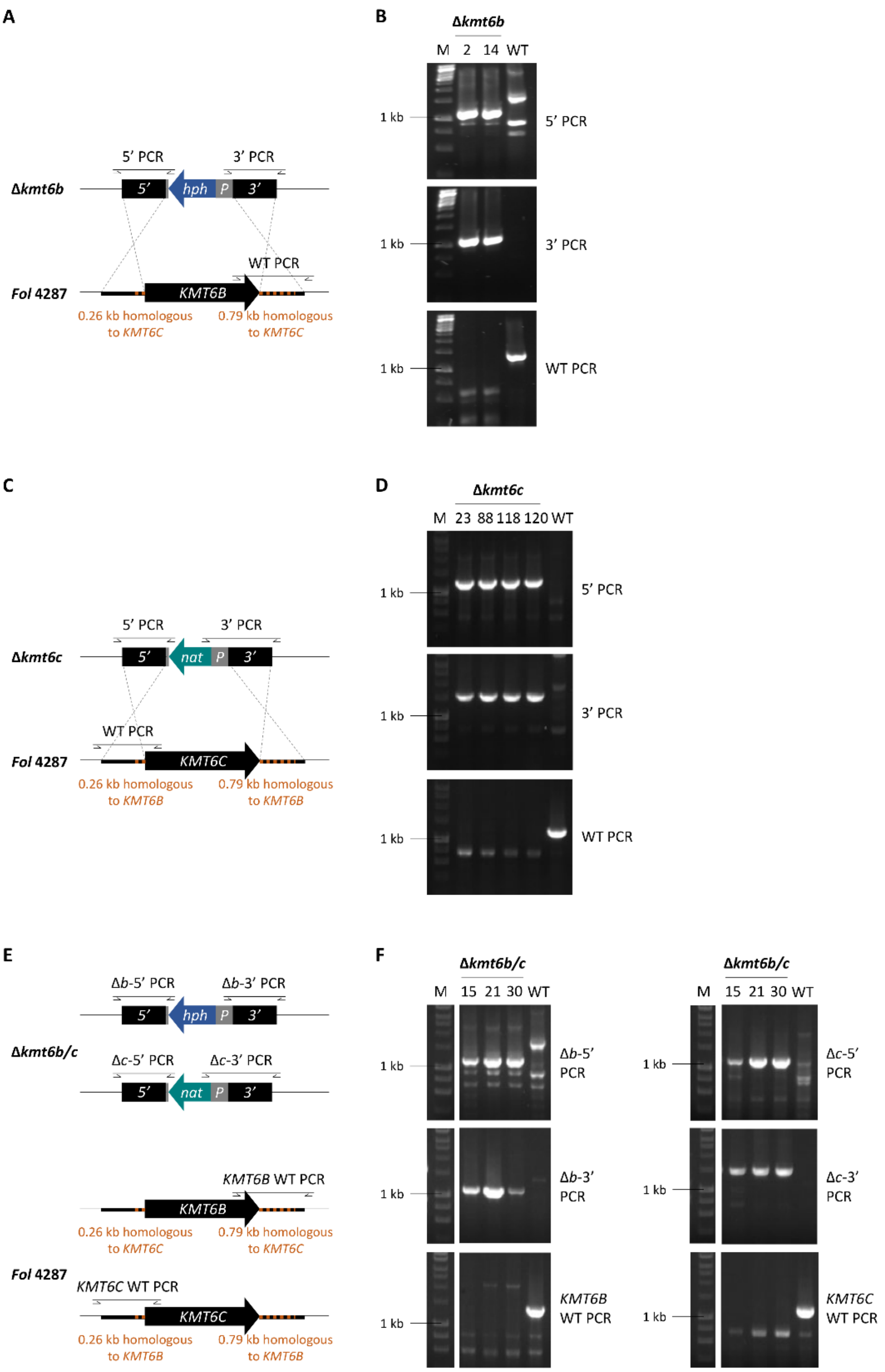

Figure S3: Verification of Δ*kmt6b/c* single and double deletion mutants. A,B) Deletion of *KMT6B via* homologous recombination and replacement with the hygromycin B resistance cassette (*hphR*) (A) was verified by amplification of 5′ (Kmt6b_5diag/trpC_T) and 3′ (Kmt6b_3diag/trpC_P2) flanks in 2 independent transformants, while no amplification was detected for the wild-type PCR (WT; Kmt6b_3diag/Kmt6b-c_F) (B). **C,D)** Deletion of *KMT6C via* homologous recombination and replacement with the nourseothricin resistance cassette (*natR*) (C) was verified by amplification of 5′ (Kmt6c_5diag/trpC_T) and 3′ (Kmt6c_3diag/nat1_seq_R) flanks in 4 independent transformants, while no amplification was detected for the WT PCR (Kmt6c_5diag/Kmt6b-c_R) (D). **E,F)** Double deletion of the *KMT6B/C* genes *via* simultaneous homologous recombination and replacement with *hphR* and *natR* (E) was verified by amplification of Δ*b*-5′ (Kmt6b_5diag/trpC_T), Δ*c*-5′ (Kmt6c_5diag/trpC_T) as well as Δ*b*-3′ (Kmt6b_3diag/trpC_P2), Δ*c*-3′ (Kmt6c_3diag/nat1_seq_R) flanks in 3 independent transformants, while no amplification was detected for the WT PCRs (Kmt6b_3diag/Kmt6b-c_F, Kmt6c_5diag/Kmt6b-c_R) (F).

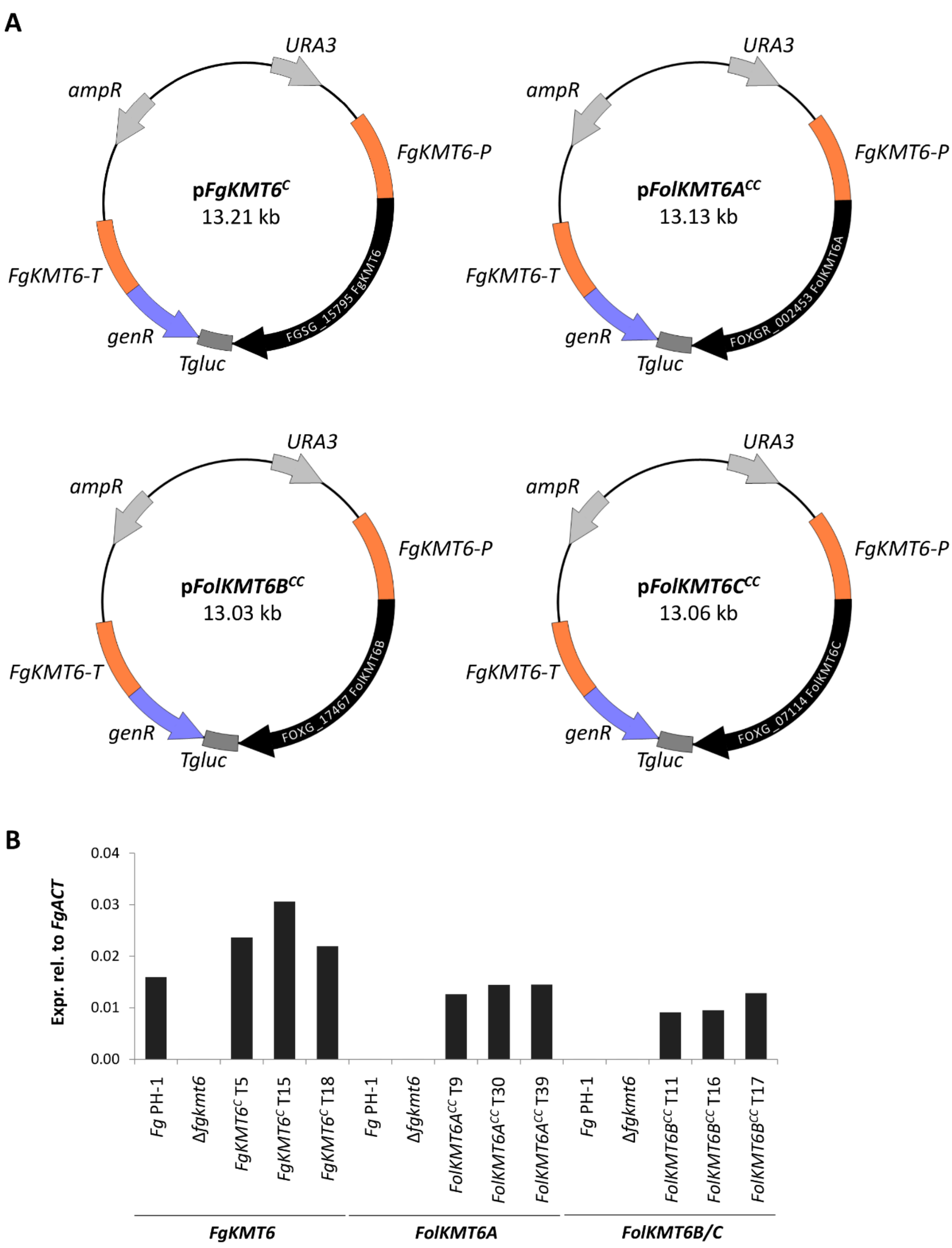

Figure S4: Verification of *F. graminearum* Δ*fgkmt6* (cross-)complemented strains. A) Plasmid maps for the expression of *FgKMT6* and *FolKMT6A-C* under the native *FgKMT6* promoter. **B)** Expression profiles of (cross-)complemented strains (n = 2).

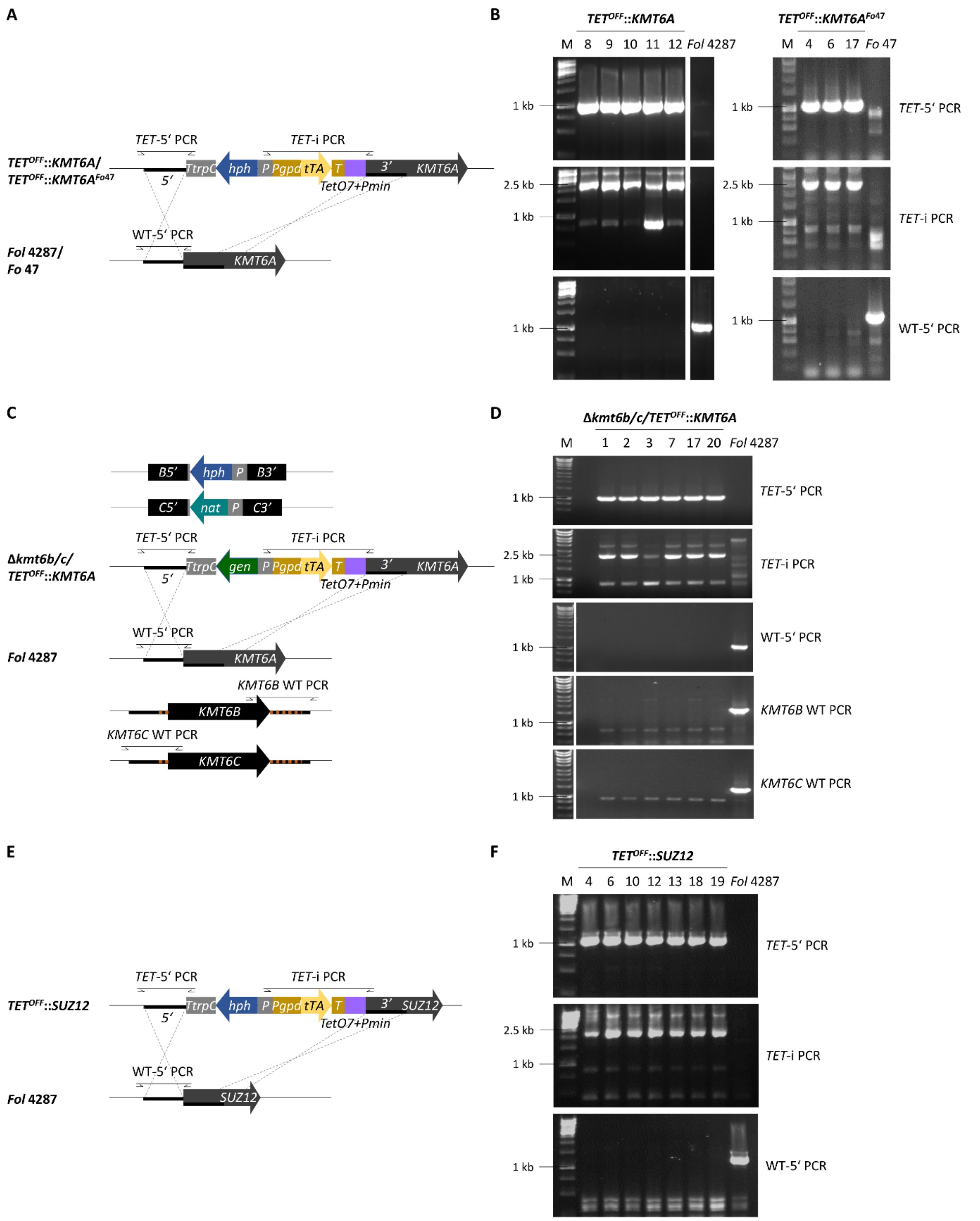

Figure S5: Verification of *TET*^{OFF}::*KMT6A* mutants in *Fol* 4287 and *Fo* 47, Δ*kmt6b/c/TET*^{OFF}::*KMT6A* and *TET*^{OFF}::*SUZ12* mutants in *Fol* 4287. A,B) Promoter exchange of *KMT6A via* homologous recombination and replacement with the *TET*^{OFF} construct using the hygromycin B resistance cassette (*hphR*) (A) was verified with the amplification of *TET*-5' (Kmt6a_5diag/TtrpC_full_diag) and *TET*-i (Kmt6a_R/trpC_P2) PCRs in 5 independent transformants of *Fol* 4287 and 3 independent transformants of *Fo* 47, while no amplification was detected for the wild-type PCR (WT 5'; Kmt6a_5diag/Kmt6a_R) (B). **C,D)** Moreover, *in locus* integration of *TET*^{OFF}::*KMT6A* using the geneticin cassette (*genR*) in the Δ*kmt6b/c* background (C) was verified with the amplification of *TET*-5' (Kmt6a_5diag/TtrpC_full_diag) and *TET*-i (Kmt6a_R/trpC_P2) PCRs in 6 independent transformants, while no amplification was detected for the wild-type PCR (WT 5'; Kmt6a_5diag/Kmt6a_R) (D). **E,F)** Promoter exchange of *SUZ12 via* homologous recombination and replacement with the *TET*^{OFF} construct using *hphR* (E) was verified by amplification of *TET*-5' (Suz12_5diag/TtrpC_full_diag) and *TET*-i (Suz12_R/trpC_P2) PCRs in 7 independent transformants of *Fol* 4287, while no amplification was detected for the WT PCR (Suz12_5diag/Suz12_R) (F).

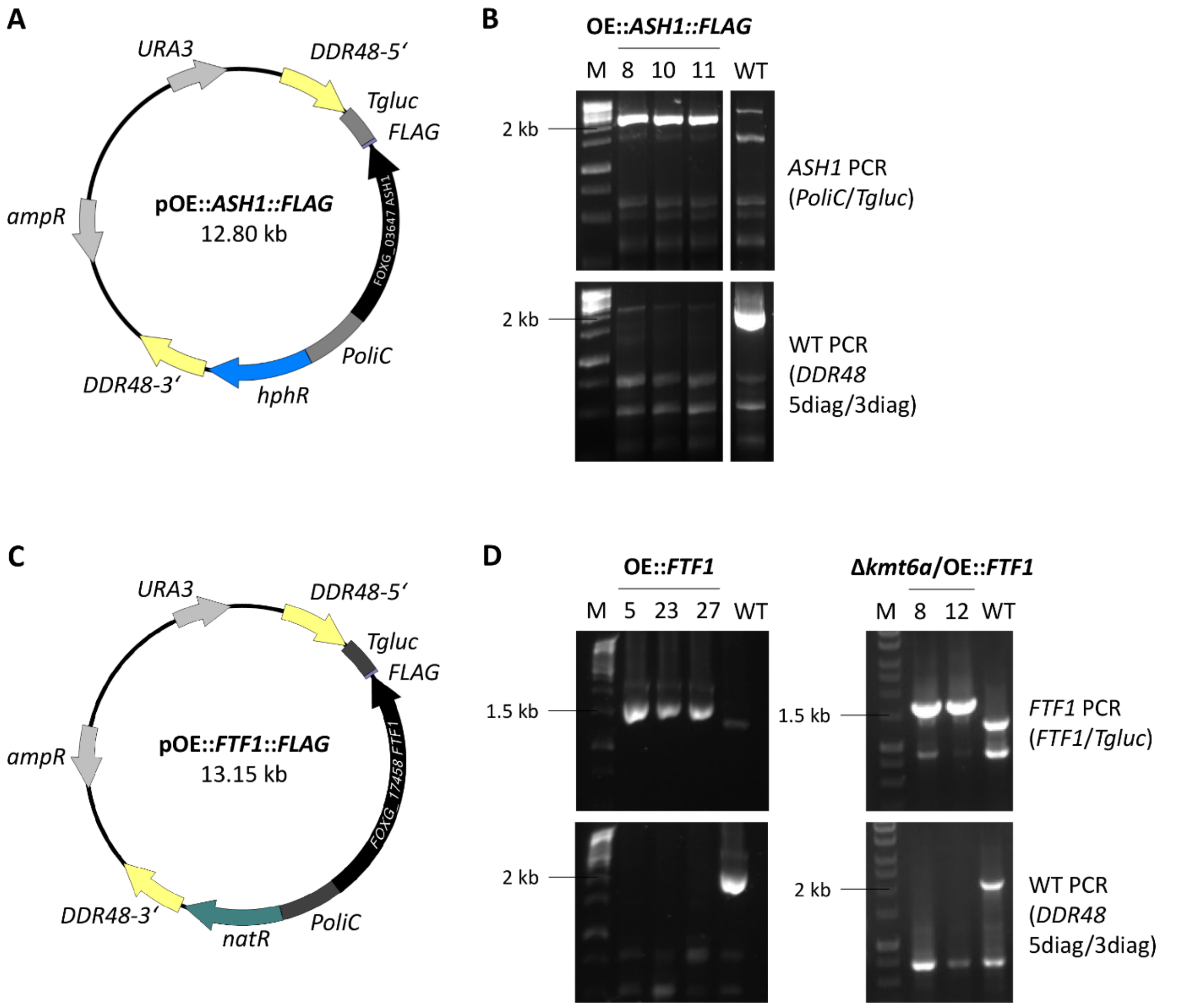

Figure S6: Verification of *ASH1* and *FTF1* overexpression strains. A,B) *ASH1* fused to *FLAG* was overexpressed by fusion to the strong and constitutive *PoliC* promoter from *A. nidulans*. *In locus* integration of the construct at the constitutive *DDR48* locus was selected with hygromycin B (*hphR*) (A) and verified by amplification of the *ASH1* PCR (PoliC_seq_F2/Tgluc_seq_R2) in 3 independent transformants, while no amplification was detected for the wild-type PCR (WT; Ddr_5diag/Ddr_3diag) (B). **C,D)** *FTF1* fused to *FLAG* was overexpressed by fusion to the *PoliC* promoter. *In locus* integration of the construct at the constitutive *DDR48* locus was selected with nourseothricin (*natR*) (C) and verified by amplification of the *FTF1* PCR (Ftf1_seq_F/Tgluc_seq_R2) in 3 independent transformants in the *Fol* 4287 WT background as well as 2 independent transformants in the Δ*kmt6a* ($T11^{original}$) background, while no amplification was detected for the WT PCR (Drd_5diag/Drd_3diag) (D).

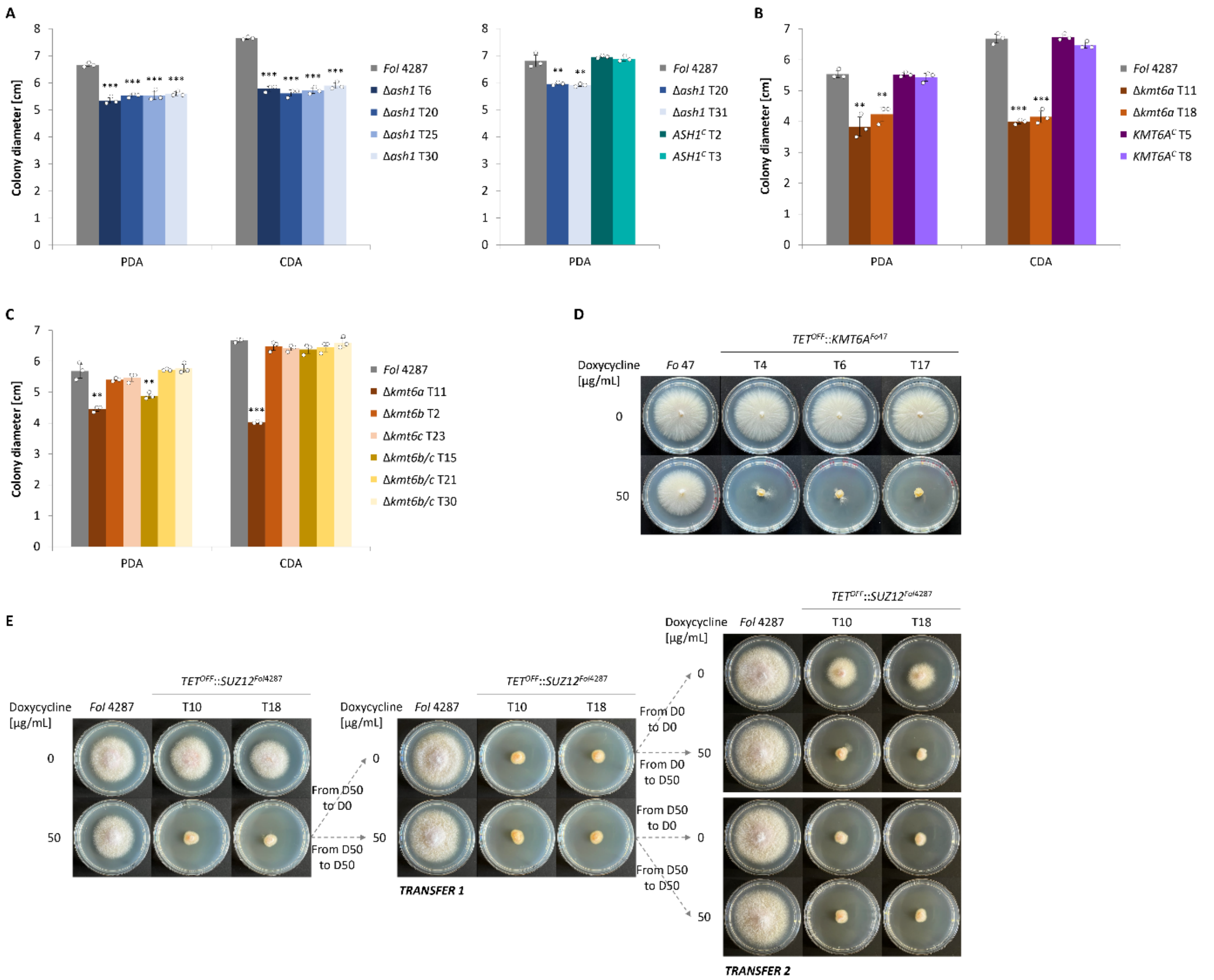

Figure S7: *TET*^{OFF}::*KMT6A*^{Fo47} in *Fo* 47 carrying a single *KMT6* homolog resembles *TET*^{OFF}::*SUZ12* mutants in *Fol* 4287. A-C) Colony growth of the wild type (WT) *Fol* 4287 and indicated mutants was assessed on complex (PDA) or minimal (CDA) media (means ± SD, n = 3). Growth of the mutants was compared to the WT for each condition using t-test; $P < 0.01$ (**), $P < 0.001$ (***). **D,E)** *Fo* 47 and its derived *TET*^{OFF}::*KMT6A*^{Fo47} strains (D), or *Fol* 4287 and its derived *TET*^{OFF}::*SUZ12* strains (E) were grown on PDA in the absence or presence of 50 µg/mL doxycycline (D0 or D50) to downregulate expression (n = 3). Two additional transfers from previous plates were inoculated as indicated.

A **Carotenoid biosynthesis (log_2FC)**

Gene		Δ*kmt6a* *vs.* WT
carX	*FOXG_12145*	6.03
carRA	*FOXG_12144*	3.33
carB	*FOXG_12143*	3.42
carO	*FOXG_12142*	6.81
carT	*FOXG_10608*	-1.11
carD	*FOXG_05463*	-0.36
ggs1	*FOXG_05311*	-0.69
carS	*FOXG_09307*	-0.81

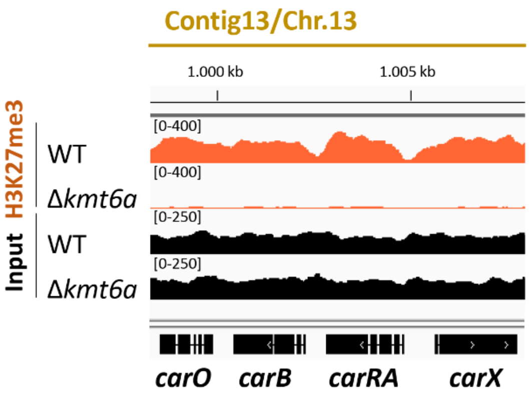

B **All predicted effectors |log_2FC| ≥ 4 (161/532)**

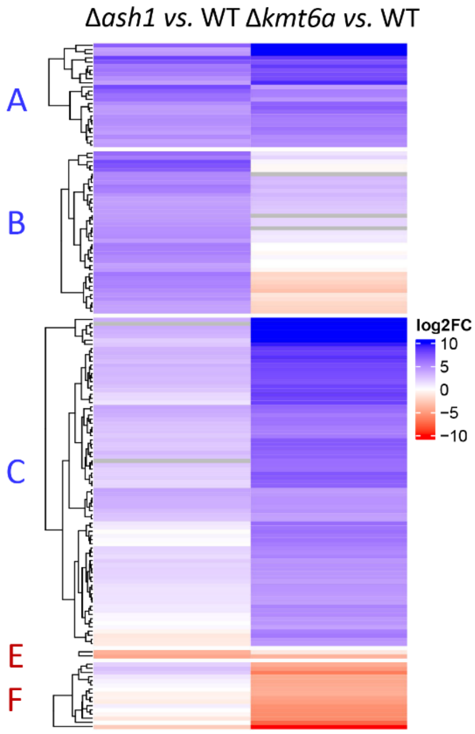

Figure S8: Carotenoid biosynthesis and effector gene expression is altered in the Δ*ash1* and Δ*kmt6a* mutants. The wild type (WT) *Fol* 4287, Δ*ash1* and Δ*kmt6a* strains were grown in biological duplicates (ChIP-seq) and quadruplicates (RNA-seq) for 2 d in liquid NO_3 medium. **A)** Extracted log_2-fold change (log_2FC) differential gene expression of the *car* gene cluster composed of *carX*, *carRA*, *carB*, *carO*, as well as the associated biosynthetic genes *carT*, *carD*, *ggs1* and the regulatory gene *carS*. Note that the 4 genes in the *car* cluster are upregulated in Δ*kmt6a*, and that H3K27me3 is markedly reduced at the respective loci. **B)** Predicted effector genes significantly up- ($log_2FC \geq 4$, FDR-corrected $P \leq 0.01$) or downregulated ($log_2FC \leq -4$, FDR-corrected $P \leq 0.01$) in at least one of the deletion mutants compared to the WT are indicated in blue or red, respectively, while not differentially expressed genes (between -4 and 4) are white. Initially, the categories were extracted (see Figure 4), and subsequently, the genes were clustered for each category. Missing genes as determined by ChIP-seq were removed from the analysis of the respective strain, and these missing values are indicated in gray in the heatmap.

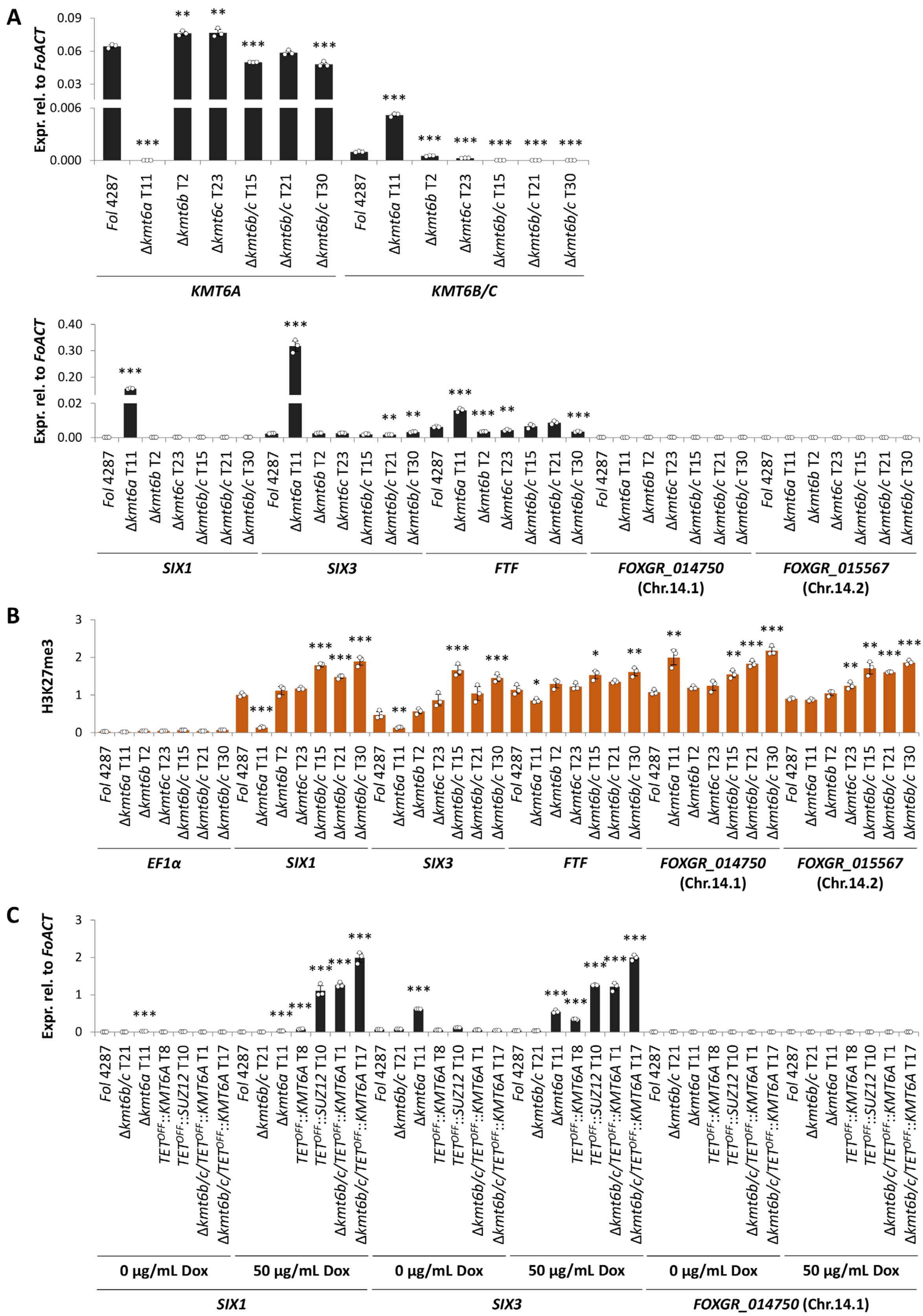

Figure S9: Expression levels in *KMT6* mutants. A,B) RT-qPCR expression analysis of 2-day-old NO_3 liquid cultures of the indicated strains (means ± SD, *n* = 3) (A), and 5'ChIP analysis using the antibody against H3K27me3 (B) under the same culture conditions (means ± SD, *n* = 3). The euchromatic housekeeping gene *EF1α* was used as a control. **C)** RT-qPCR expression analysis of 2-day-old NO_3 liquid cultures of the indicated strains (means ± SD, *n* = 3). The transcript or enrichment levels were compared between mutants and wild type for each gene and condition using *t*-test; $P < 0.01$ (**), $P < 0.001$ (***).

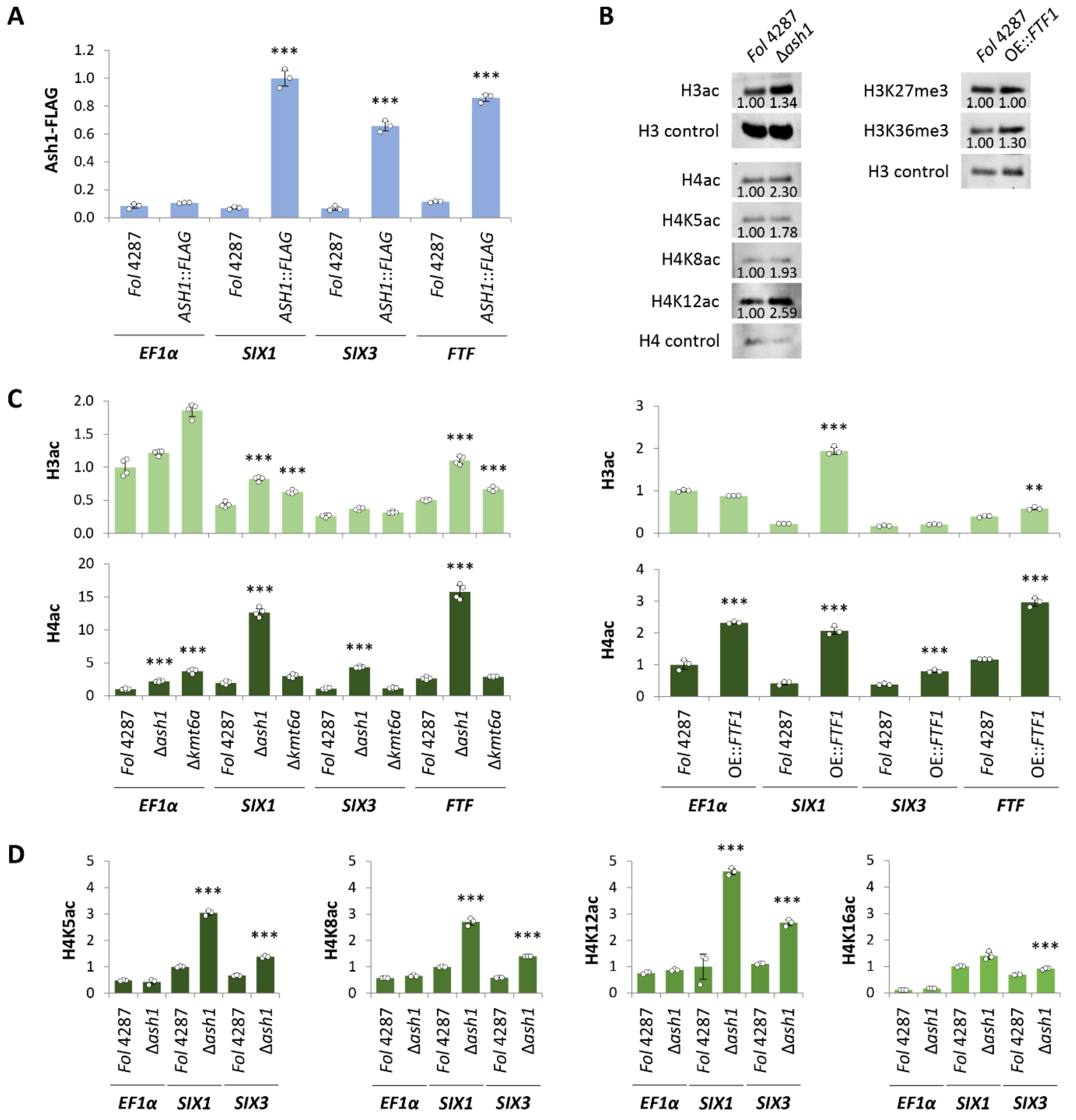

Figure S10: H4 acetylation is increased in Δ*ash1*. A) 5'ChIP analysis of 2-day-old NO_3 liquid cultures using the antibody against FLAG for the strain *ASH1*::*FLAG* (means ± SD, *n* = 3). **B)** Genome-wide histone modification levels were analyzed in Δ*ash1* and OE::*FTF1* relative to the wild type (NO_3, 2 d) by western blot using indicated specific antibodies. Bands were quantified by densitometry and normalized to the H3/H4 controls. **C)** 5'ChIP analysis using specific antibodies against H3ac (light green) or H4ac (dark green) for the Δ*ash1* and Δ*kmt6a* mutants (means ± SD, *n* = 4) (left) or the OE::*FTF1* strain (means ± SD, *n* = 3) (right). **D)** 5'ChIP analysis using specific antibodies against H4K5/K8/K12/K16ac for the Δ*ash1* mutant (means ± SD, *n* = 3). Enrichment levels were compared between mutants and wild type for each gene using *t*-test; $P < 0.01$ (**), $P < 0.001$ (***).

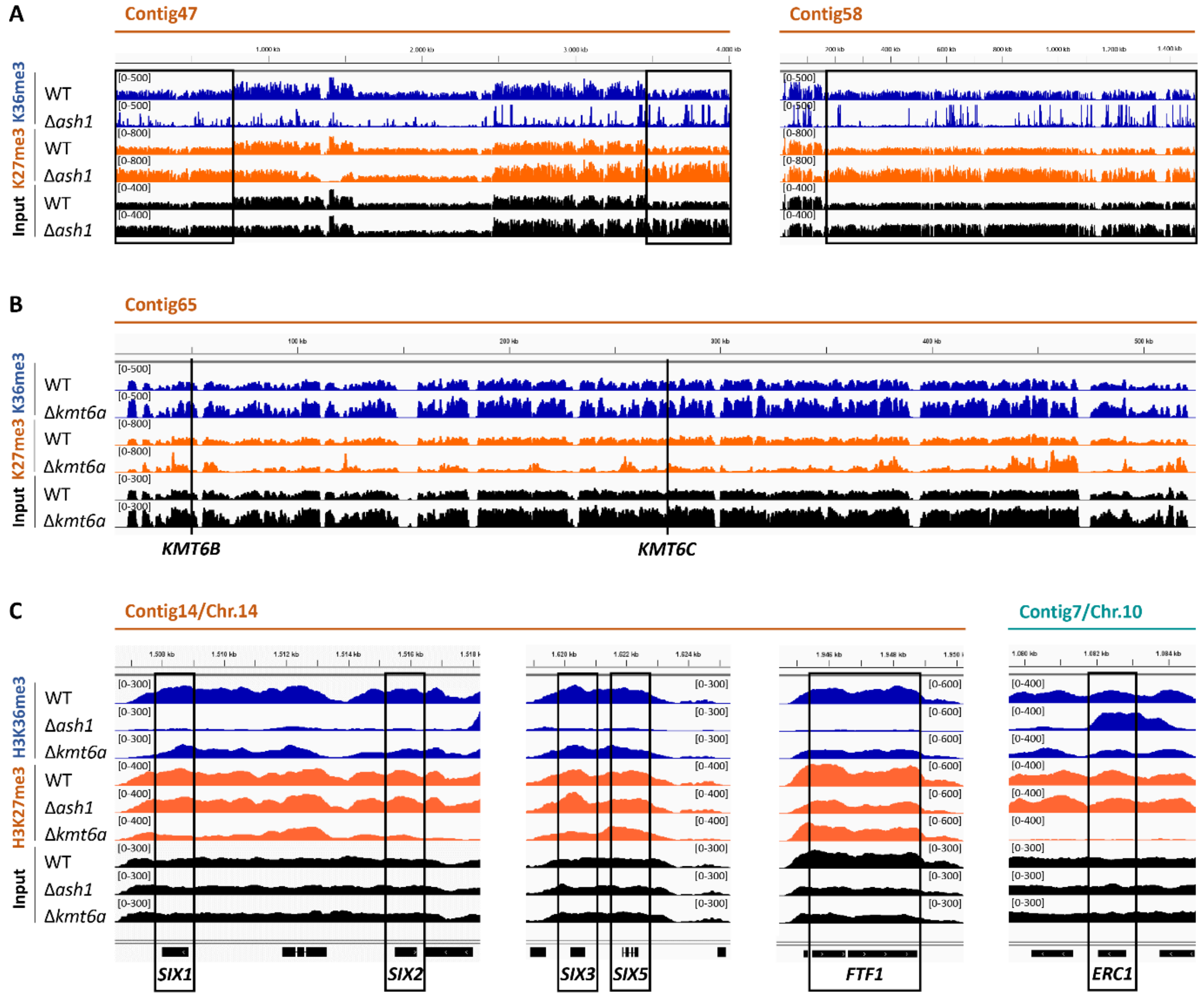

Figure S11: ChIP-seq methylation levels visualized by the Integrative Genome Viewer. *Fol* 4287 and the Δ*ash1* and Δ*kmt6a* deletion mutants were grown in biological duplicates for 2 d in liquid NO_3 medium, prior to ChIP-seq analysis using the indicated antibodies. Input samples were not treated with antibody. **A)** Duplicated regions of contigs 47 and 58 in Δ*ash1*; **B)** duplicated *KMT6B/C* genes on contig 65 in Δ*kmt6a*; **C)** *SIX* effector genes (*SIX1*, *SIX2*, *SIX3*, *SIX5*), *FTF1* and *ERC1* on the indicated contigs in all strains.

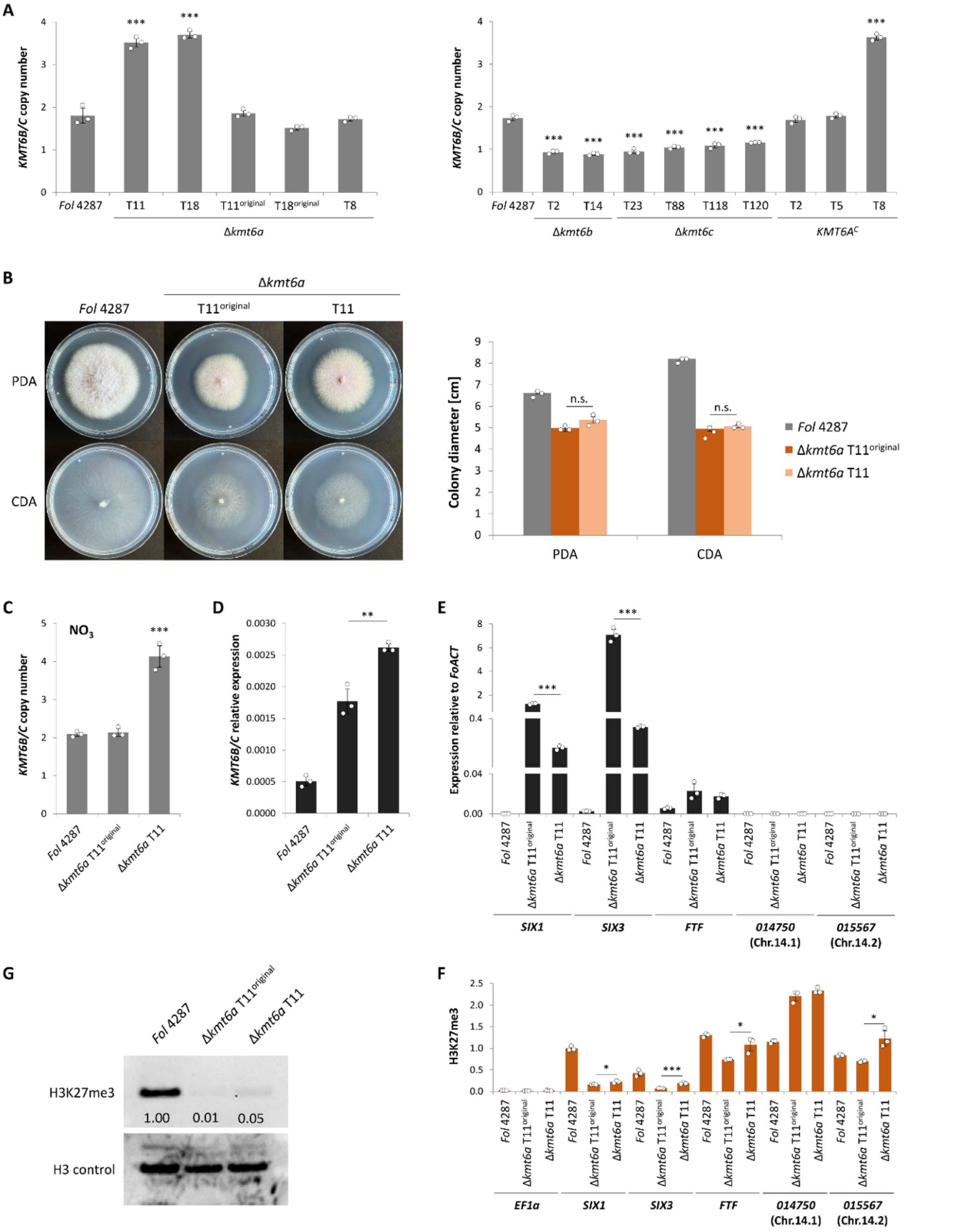

Figure S12: Comparison of Δ*kmt6a* T11 carrying either 2 (original) or 4 copies of *KMT6B/C*. A) *KMT6B/C* copy number in relation to the single copy gene *EF1α* was determined by 3'qPCR on gDNA (means ± SD, *n* = 3). Both $KMT6A^C$ T5 (2 copies) and $KMT6A^C$ T8 (4 copies) originate from a heterokaryotic colony of Δ*kmt6a* T11, the latter being distinguished between "original" (2 copies) and "evolved" (4 copies) deletion mutant. **B)** Colony growth of the indicated strains was assessed on complex (PDA) and minimal (CDA) media (means ± SD, *n* = 3). **C)** *KMT6B/C* copy number was measured as described above using gDNA extracted from 2-day-old NO_3 liquid cultures of the indicated strains. **D-F)** RT-qPCR expression analysis (D,E), or 5'ChIP analysis with the H3K27me3 antibody (F) of the indicated genes in cultures grown for 2 d in liquid NO_3 medium (means ± SD, *n* = 3). The euchromatic gene *EF1α* was used as a control. **G)** Genome-wide H3K27me3 levels were determined by western blot analysis. Bands were quantified by densitometry and normalized to the H3 control. Copy number, transcript or enrichment levels for each gene were compared between mutants and wild type or as indicated, using *t*-test; $P < 0.05$ (*), $P < 0.01$ (**), $P < 0.001$ (***), ns, not significant.

A

**ChIP-seq** **FC over background ≥ 4**	Affected genes		in fHC	%
Δash1, H3K36me3 down	1522	↑	1368	89.88
Δash1, H3K27me3 down	10	67	9	90.00
Δkmt6a, H3K36me3 down	14		13	92.86
Δkmt6a, H3K27me3 down	347	↓	335	96.54
Δash1, H3K36me3 up	18		11	61.11
Δash1, H3K27me3 up	3		0	0.00
Δkmt6a, H3K36me3 up	4		1	25.00
Δkmt6a, H3K27me3 up	9		1	11.11
**Sum**	**1927**		**1738**	**90.19**

B

**RNA-seq**	A (+,+)	B (+,0)	C (0,+)	D (-,-)	E (-,0)	F (0,-)	G (+,-)	H (-,+)	Total	%
**$|log_2FC| \geq 2$**	1415	2226	1752	52	73	1530	256	6	7310/26826	27.25
Δash1, H3K36me3 down	203	309	103	4	1	109	32	0	761/1522	50.00
Δash1, H3K27me3 down	0	6	0	0	0	0	0	0	6/10	60.00
Δkmt6a, H3K36me3 down	3	0	3	0	0	1	0	0	7/14	50.00
Δkmt6a, H3K27me3 down	77	14	93	0	1	3	0	0	188/347	53.60
Δash1, H3K36me3 up	0	1	2	0	0	0	0	0	3/18	16.67
Δash1, H3K27me3 up	0	0	0	0	0	2	0	0	2/3	66.67
Δkmt6a, H3K36me3 up	0	1	0	0	0	1	0	0	2/4	50.00
Δkmt6a, H3K27me3 up	0	0	0	0	0	0	0	0	0/9	0.00
Sum	283/1415	331/2226	201/1752	4/52	2/73	114/1530	32/256	0/6	**967/1927**	**Total**
%	20.00	14.87	11.47	7.69	2.74	7.45	12.50	0	**50.18**	**% of ChIP-seq covered by RNA-seq**

C

**ChIP-seq sample significantly correlated**	**RNA-seq category**	**GeneRatio**	**BgRatio**	**pvalue**	**p.adjust**	**qvalue**	**Count**
Δash1, H3K36me3 down	B (+,0)	309/761	2226/7310	2.06E-10	1.45E-09	1.09E-09	309
	A (+,+)	203/761	1415/7310	1.19E-07	4.16E-07	3.13E-07	203
Δkmt6a, H3K27me3 down	C (0,+)	93/188	1752/7310	1.36E-14	6.79E-14	4.29E-14	93
	A (+,+)	77/188	1415/7310	3.94E-12	9.84E-12	6.22E-12	77

Figure S13: Quantification of the ChIP-seq and correlation with RNA-seq. The wild type (WT) *Fol* 4287, Δ*ash1* and Δ*kmt6a* strains were grown in biological duplicates (ChIP-seq) and quadruplicates (RNA-seq) for 2 d in liquid NO_3 medium. Genes lost from the genomes of Δ*ash1* or Δ*kmt6a* were omitted in the analysis. **A)** Methylation marks lost (down) or gained (up) in Δ*ash1* and Δ*kmt6a* compared to the WT, which are associated with genes, *i.e.*, gene bodies plus 1 kb upstream. The percentage of genes associated with facultative heterochromatin (fHC), defined as marked with H3K27me3 in the WT, is indicated. The criteria ≥ 4-fold enrichment over background and Poisson enrichment *P* value ≤ 0.0001 were applied to select significantly different peaks. **B)** Comparison between ChIP- and RNA-seq ($|log_2FC| \geq 2$, FDR-corrected $P \leq 0.001$), using the RNA-seq categories from Figure 4. Genes identified by both genome-wide methods are indicated. **C)** Significant correlations between ChIP- and RNA-seq were identified for the RNA-seq categories A, B and C.

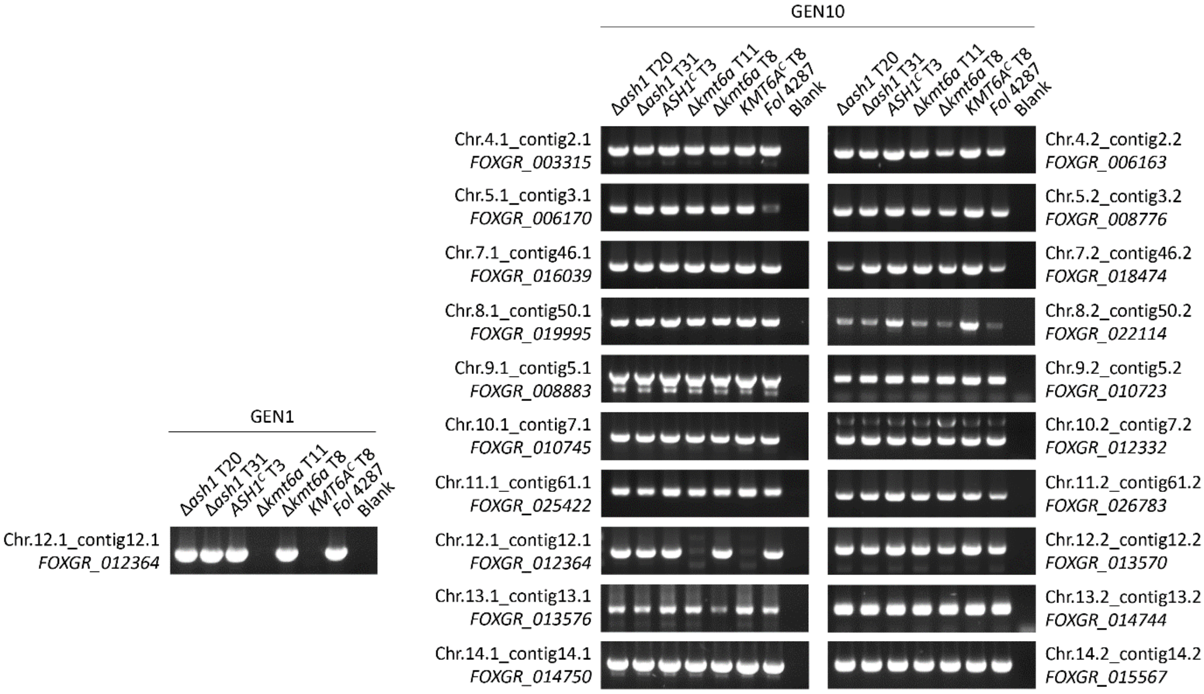

Figure S14: PCR analysis of subtelomere stability using specific primers. The wild-type (WT) strain *Fol* 4287, two independent Δ*ash1* and Δ*kmt6a* deletion mutants, as well as one *ASH1*^{C} and *KMT6A*^{C} complemented strain, respectively, were analyzed before and after 10 serial passages on PDA solid medium (3 days of growth per passage), designated GEN1 and GEN10 (generation). *KMT6A*^{C} originates from Δ*kmt6a* T11. Primers are listed in Table S1.

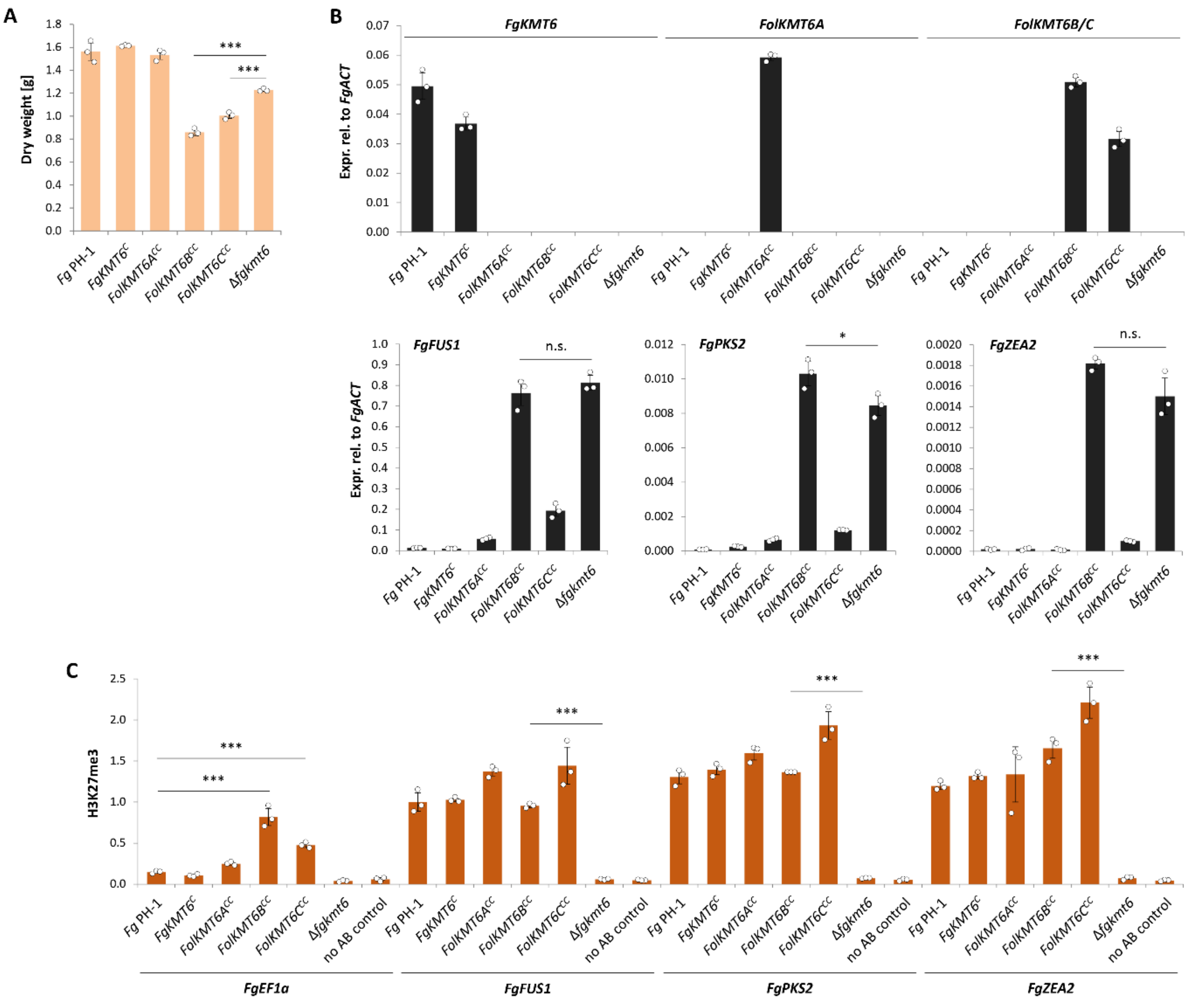

Figure S15: Expression levels in *FolKMT6A-C* cross-complemented strains of *F. graminearum*. A) Dry weight of cultures grown in triplicate in ICI with 60 mM glutamine for 3 days. **B,C)** RT-qPCR expression analysis (B), or 5'ChIP analysis with the H3K27me3 antibody (C) of the indicated genes in cultures grown under the same conditions (means ± SD, $n = 3$). The euchromatic gene *FgEF1α* was used as a control. Dry weight, transcript and enrichment levels for each gene were compared as indicated, using *t*-test; $P < 0.05$ (*), $P < 0.01$ (**), $P < 0.001$ (***), ns, not significant.

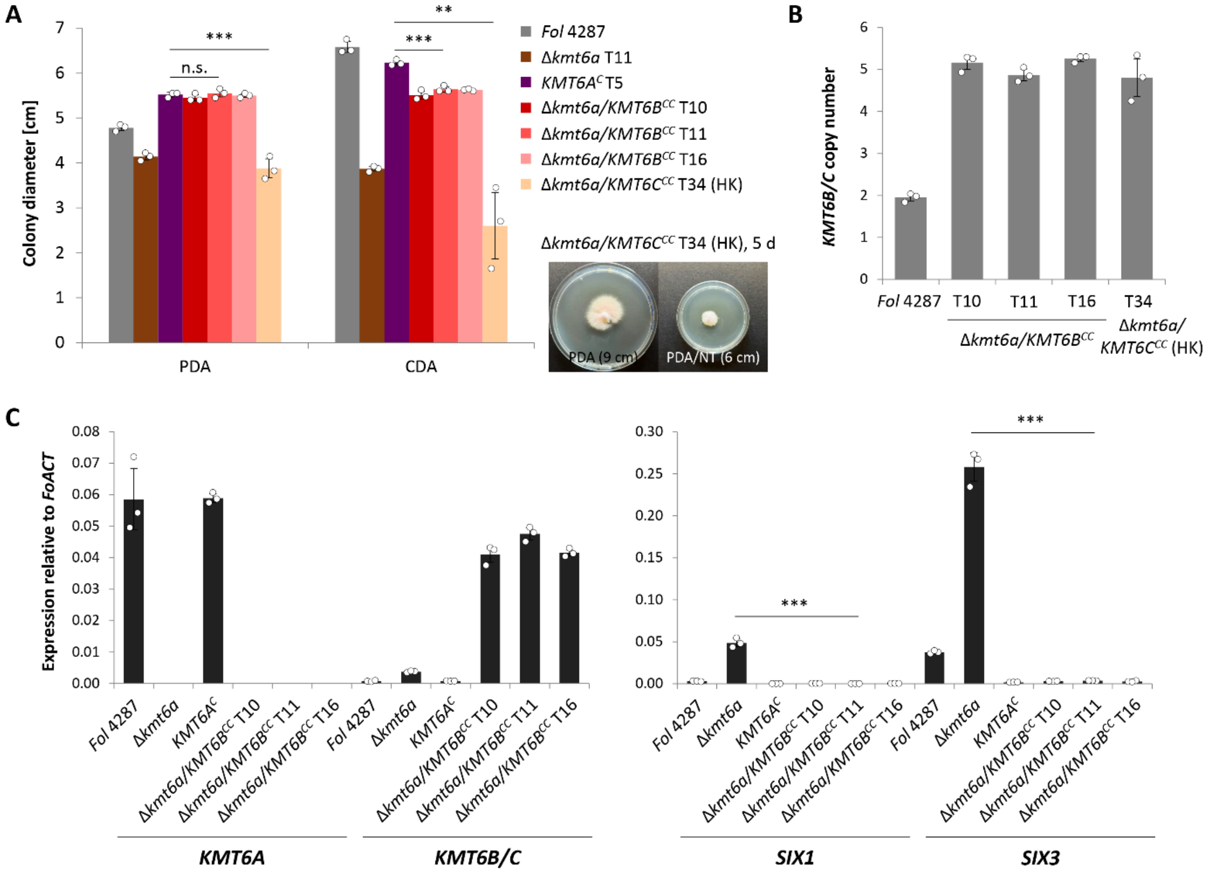

Figure S16: *KMT6B/C* cross-complementation of *Fol* Δ*kmt6a*. A) Colony growth of the wild type *Fol* 4287 and indicated mutants was assessed on complex (PDA) or minimal (CDA) media (means ± SD, *n* = 3). The heterokaryotic (HK) transformant of *KMT6C*^{CC} was compared after 5 days of growth on PDA without and with the resistance nourseothricin (NT). Big (9 cm) and small (6 cm) plates were used, respectively, and are shown to scale. **B)** *KMT6B/C* copy number in relation to the single copy gene *EF1α* was determined by 3'qPCR on gDNA (means ± SD, *n* = 3). Since the evolved strain Δ*kmt6a* T11 was used, cross-complemented strains harbor 5 copies of *KMT6B/C*. **C)** RT-qPCR expression analysis of 2-day-old NO_3 liquid cultures of the indicated strains (means ± SD, *n* = 3). Growth and transcript levels for each gene were compared as indicated, using *t*-test; $P < 0.01$ (**), $P < 0.001$ (***), ns, not significant.

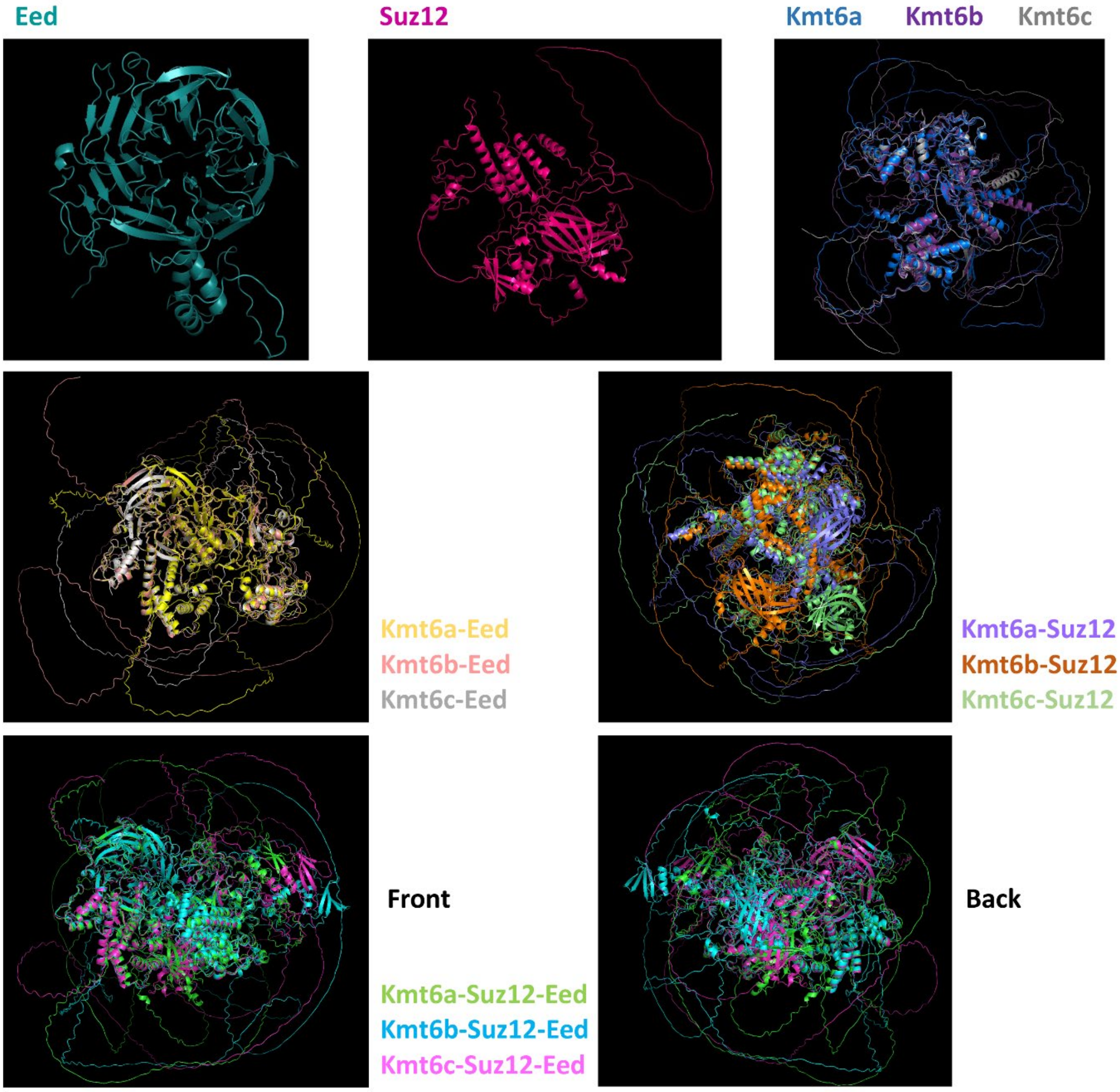

Figure S17: Modeling of protein structures or of the PRC2 (sub-)complex using AlphaFold 3. Aligments of predicted structures were done in Pymol.

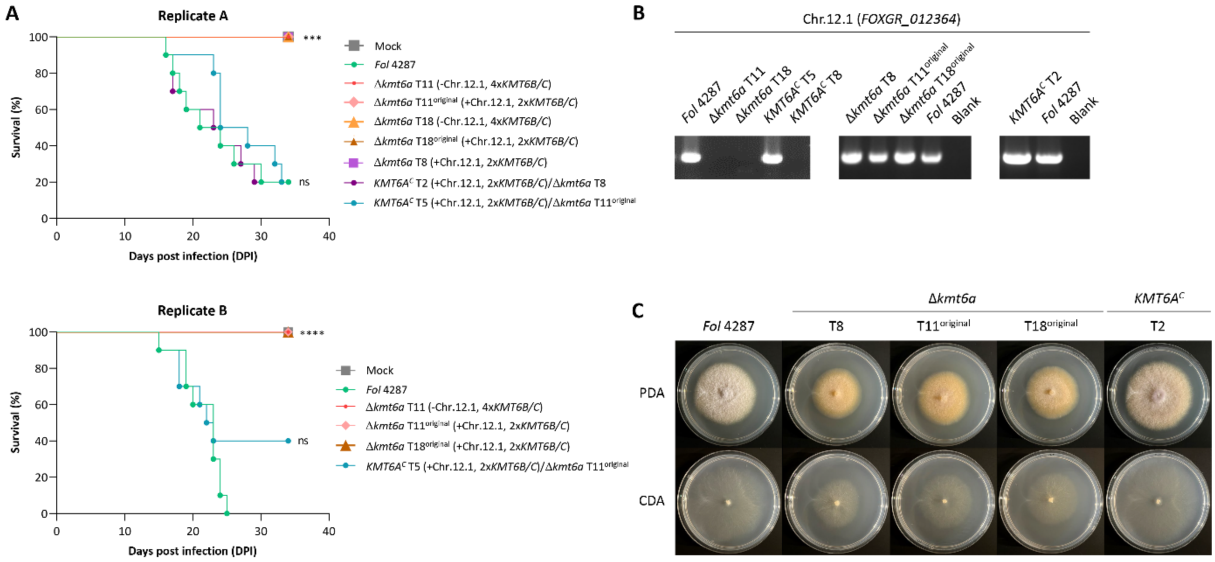

Figure S18: The role of Kmt6a in pathogenicity of *Fol* 4287 on tomato plants is independent of the presence/loss of the subtelomeric region of chromosome 12 or of the presence of 2 or 4 total copies of *KMT6B/C*. A) Kaplan-Meier plots showing survival of tomato plants inoculated by dipping roots into a suspension of $5x10^6$ freshly obtained microconidia/mL of the indicated fungal strains. Percentage survival was recorded for 35 days. Three independent experiments with 10 plants per treatment were performed. Data shown are from two representative experiments. *P* < 0.001 (***); non-significant (ns) *vs. Fol* 4287 according to log-rank test. **B)** PCR analysis to detect presence or loss of the Chr.12.1 subtelomeric region using gene-specific primers (see Table S1). Δ*kmt6a* $T11^{original}$ and $T18^{original}$ refer to original glycerol stocks with the intact chromosome 12 and 2 copies of *KMT6B/C*, while the evolved transformants are lacking the subtelomeric region of chromosome 12 and have a total of 4 copies of *KMT6B/C* (see also Figure S12A). **C)** Colony growth on PDA and CDA media of the indicated *KMT6A* deletion mutants with full-length chromosome 12 and 2 copies of *KMT6B/C* (*n* = 3).

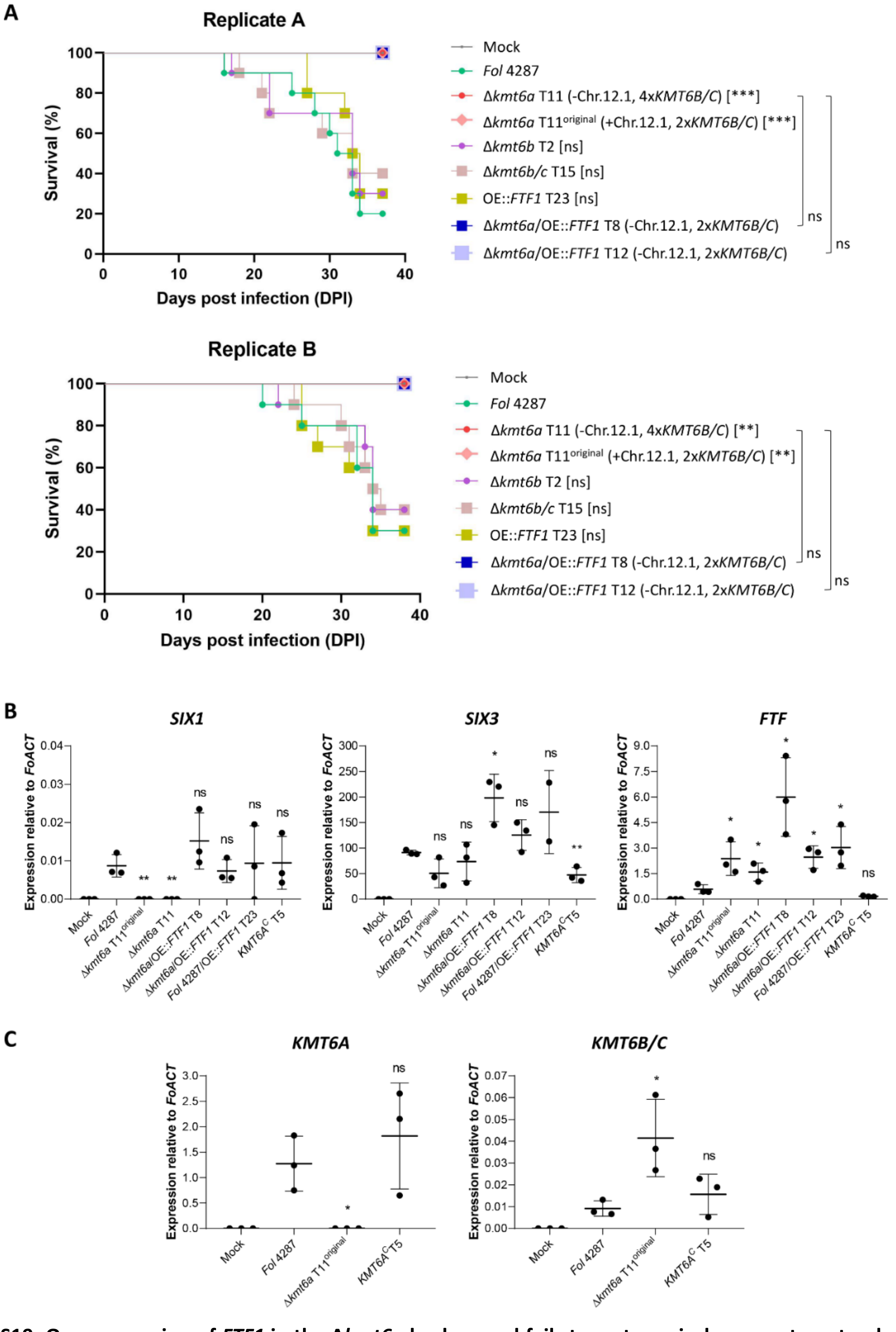

Figure S19: Overexpression of *FTF1* in the Δ*kmt6a* background fails to restore virulence on tomato plants. A) Kaplan-Meier plots showing survival of tomato plants inoculated by dipping roots into a suspension of $5x10^6$ freshly obtained microconidia/mL of the indicated fungal strains. Percentage survival was recorded for 38 days. Two independent experiments with 10 plants per treatment were performed. $P < 0.01$ (**); $P < 0.001$ (***); non-significant (ns) *vs. Fol* 4287 or Δ*kmt6a* T11 (for Δ*kmt6a*/OE::FTF1 strains) according to log-rank test. We chose the Δ*kmt6b/c* transformant T15 which showed the slight growth defect (Figure 1F). **B,C)** Transcript levels of the fungal virulence-associated *SIX1*, *SIX3* and *FTF*-type genes (B) and of *KMT6A-C* (C) were measured by RT-qPCR in cDNA samples obtained from infected tomato roots at 6 DPI. Relative transcript levels are normalized to the fungal actin gene. Data shown represent the mean and standard deviations of three independent biological replicates. $P < 0.05$ (*); $P < 0.01$ (**); non-significant (ns) *vs. Fol* 4287 according to unpaired *t*-test.

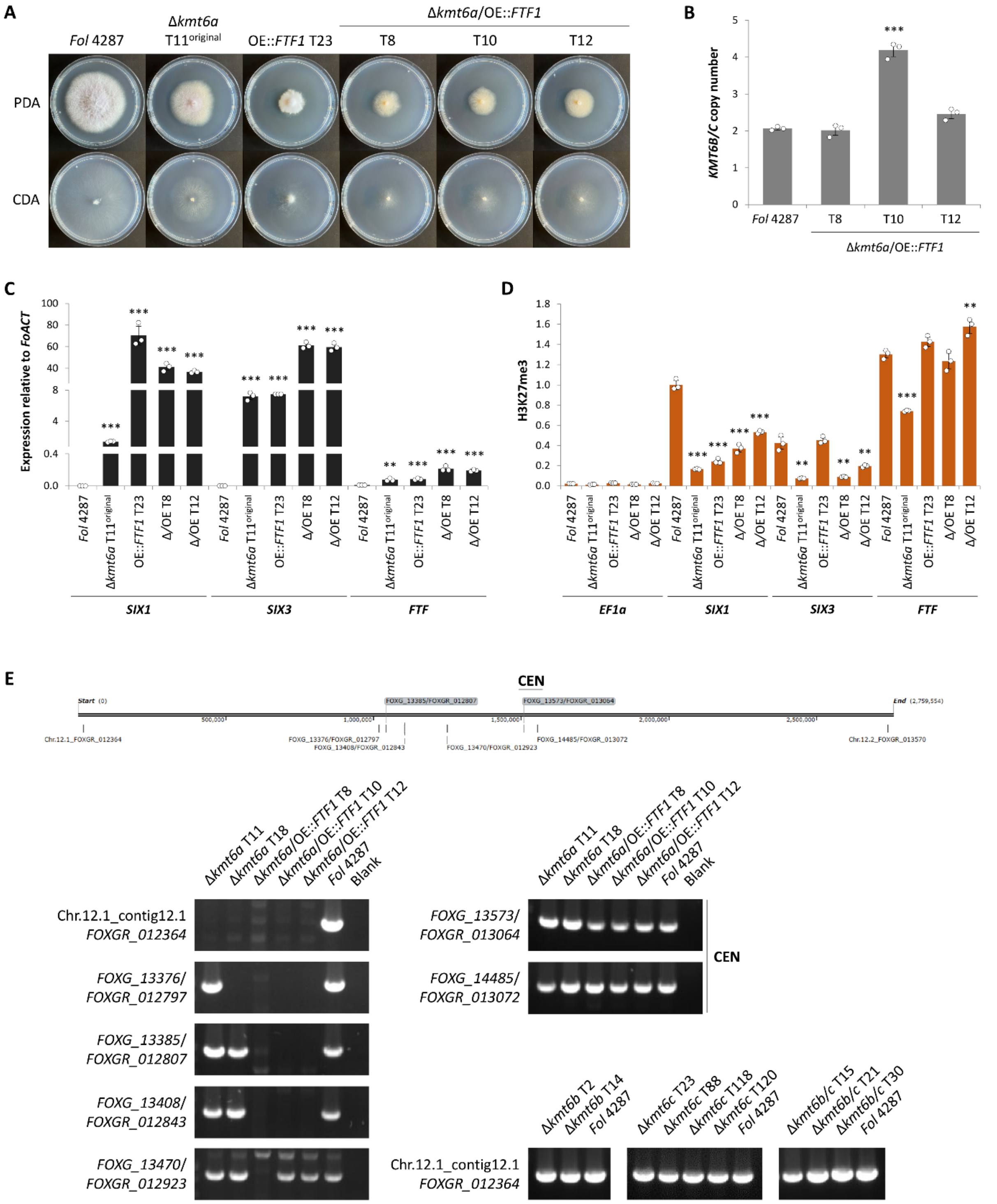

Figure S20: Overexpression of *FTF1* in the Δ*kmt6a* background results in strongly upregulated effector gene expression under axenic conditions. A) Colony growth on complex (PDA) and minimal (CDA) media of the wild-type (WT) strain *Fol* 4287 and the indicated mutants (n = 3). **B)** *KMT6B/C* copy number was determined in relation to the single copy gene *EF1α* by 3'qPCR on gDNA (means ± SD, n = 3). **C,D)** RT-qPCR expression analysis (C) or 5'ChIP analysis with the H3K27me3 antibody (D) of the indicated genes from 2-day-old NO_3 liquid cultures (means ± SD, n = 3). The euchromatic gene *EF1α* was used as a control. Copy number, transcript or enrichment levels for each gene were compared between mutants and WT using t-test; $P < 0.01$ (**), $P < 0.001$ (***). **E)** PCR analysis to detect presence or loss of the Chr.12.1 subtelomeric region using primers listed in Table S1. Primer locations across the chromosome are indicated. All Δ*kmt6a* transformants contain sequences on both sides of the predicted centromere (CEN).

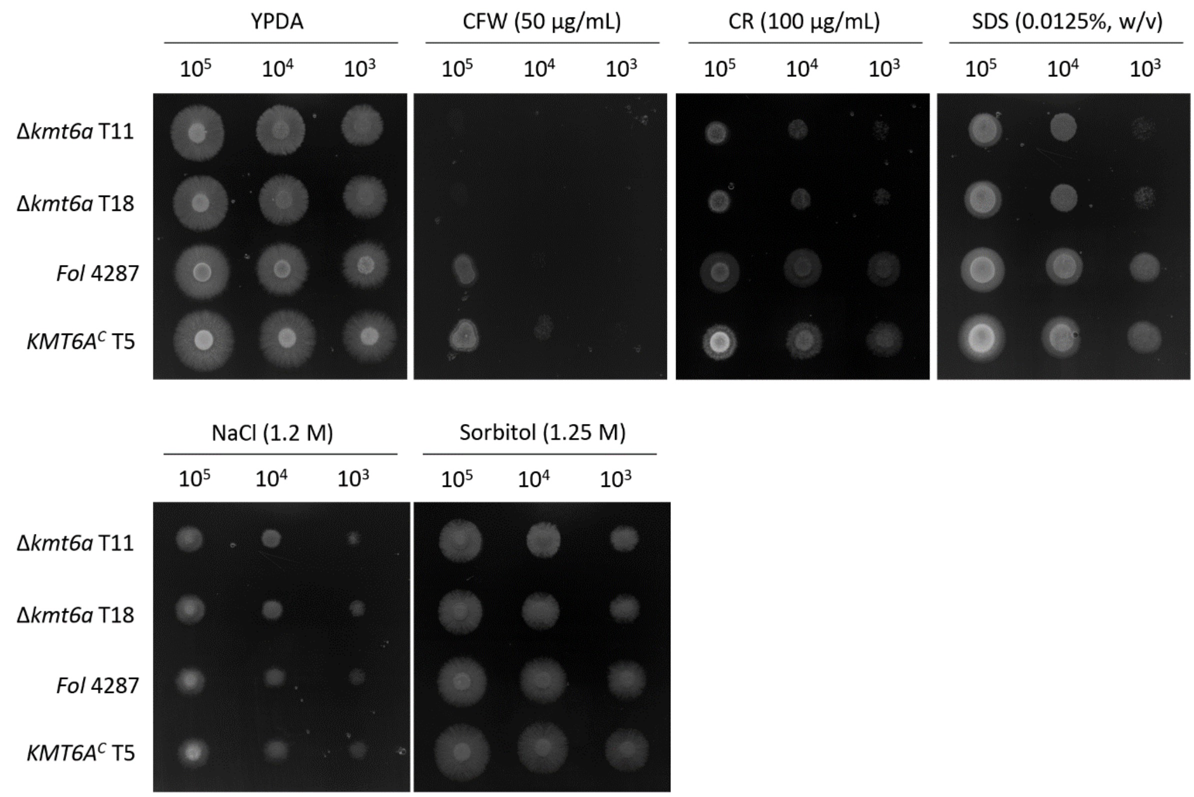

Figure S21: Loss of *KMT6A* increases sensitivity to cell wall- and membrane-damaging agents. Droplets from serial dilutions containing 10^5, 10^4 or 10^3 microconidia of the indicated fungal strains were spot-inoculated on YPDA plates supplemented with calcofluor white (CFW; 50 µg/mL), congo red (CR; 100 µg/mL), sodium dodecyl sulfate (SDS; 0.0125%, w/v), sodium chloride (NaCl; 1.2 M) or sorbitol (1.25 M). Plates were incubated for 3 days at 28 °C and scanned. Images shown are representative of three biological replicates.

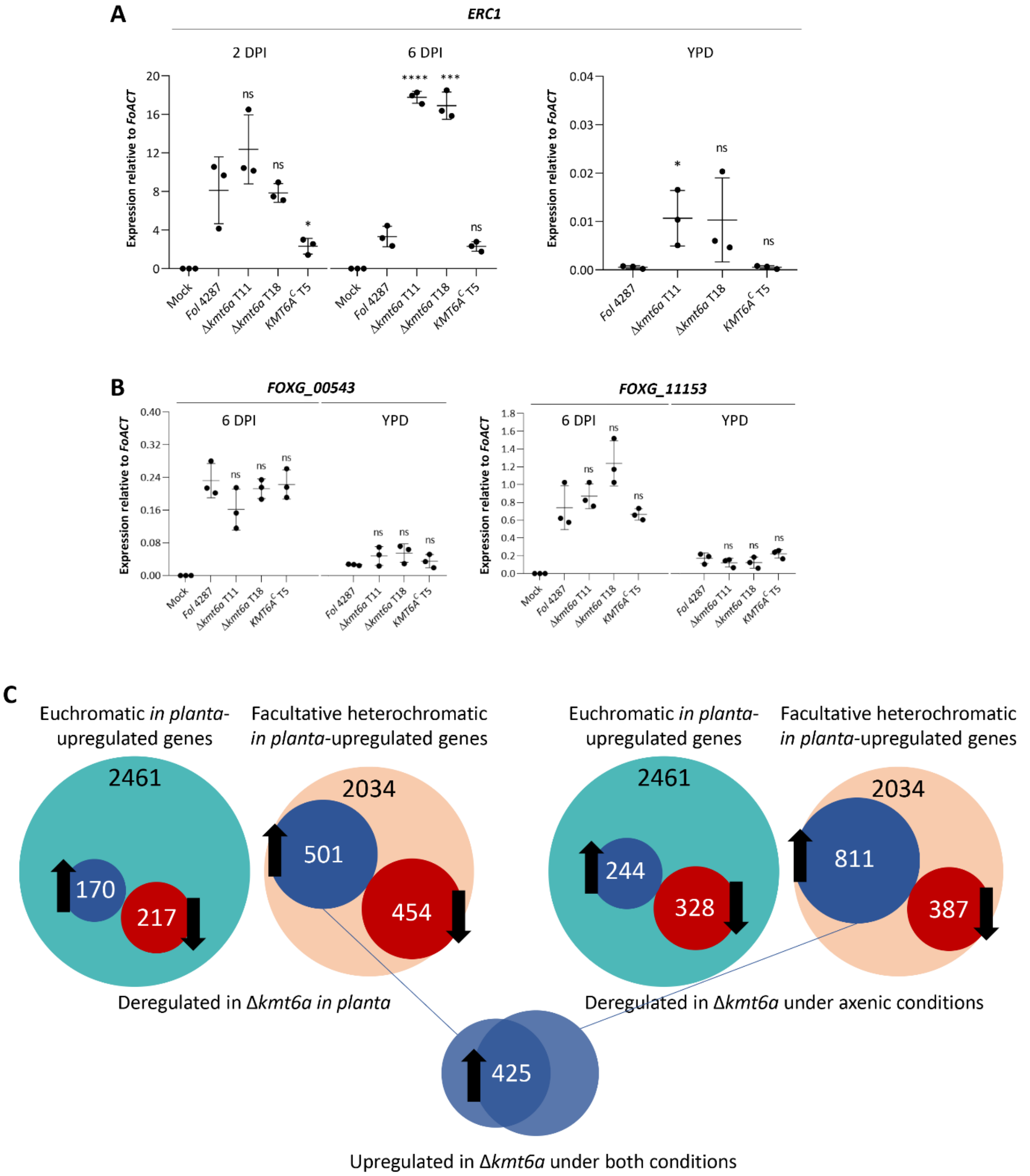

Figure S22: Infection of the Δ*kmt6a* mutants is blocked in the early root colonization stages. A) Transcript levels of the *ERC1* (Early Root Colonization) effector gene were measured by RT-qPCR in cDNA samples obtained from tomato roots at 2 or 6 days after inoculation (DPI) with the indicated fungal strains (left graph); or after 14 hours germination in liquid YPD medium (right graph). Relative transcript levels are normalized to the fungal actin gene. Data shown represent the mean and standard deviations of three independent biological replicates. $P < 0.05$ (*); $P < 0.001$ (***); $P < 0.0001$ (****); non-significant (ns) *vs. Fol* 4287 according to unpaired *t*-test. **B)** Expression pattern of two *in planta* induced genes marked with H3K4me2 (euchromatin) under axenic conditions is not altered. Transcript levels of the *FOXG_00543* and *FOXG_11153* genes were measured by RT-qPCR in cDNA samples obtained from tomato roots at 6 DPI with the indicated fungal strains (left side); or after 14 hours germination in liquid YPD medium (right side). Relative transcript levels are normalized to the fungal actin gene. Data shown represent the mean and standard deviations of three independent biological replicates. Non-significant (ns) *vs. Fol* 4287 according to unpaired *t*-test. **C)** Genes upregulated *in planta vs.* axenic conditions in the wild type (shown twice to delineate further filtering), were filtered for their association with H3K27me3 (classified as facultative heterochromatic) or not (classified as euchromatic), and further filtered according to their deregulation in Δ*kmt6a in planta* (6 DPI) or under axenic conditions (NO_3, 2 d), using $|\log_2 FC| \geq 1$, FDR-corrected $P \leq 0.01$.

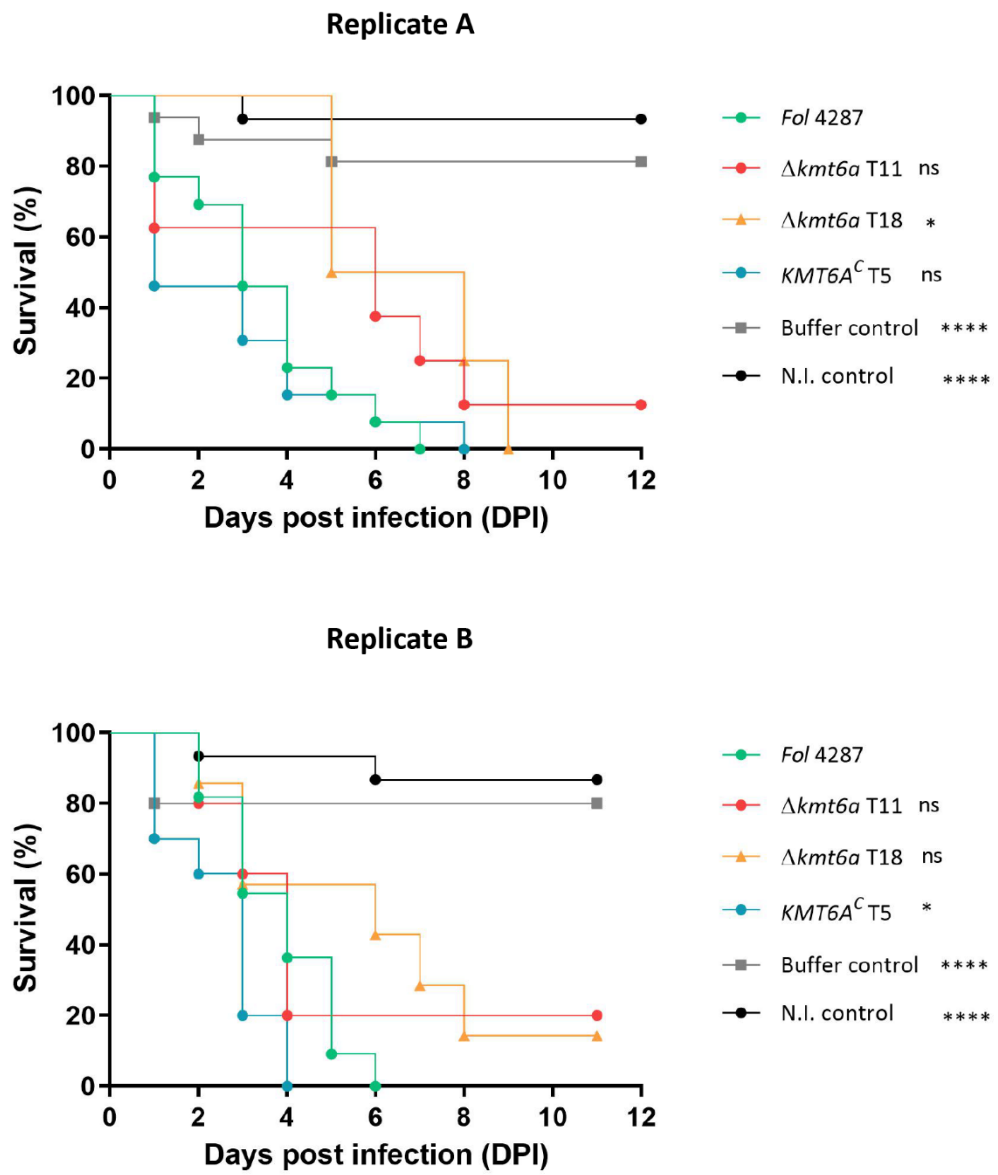

Figure S23: Kmt6a is not required for virulence on the model animal host *Galleria mellonella*. Kaplan-Meier plots showing survival of *G. mellonella* larvae at 30 °C after injection into the hemocoel of $1.6x10^4$ freshly obtained microconidia of the indicated fungal strains. Non-injected (N.I. control) and PBS-injected (buffer control) larvae as control treatments. Percentage survival was recorded for 11-12 days. Two independent experiments with 15 larvae per treatment were performed. $P < 0.05$ (*); $P < 0.0001$ (****); non-significant (ns); *vs. Fol* 4287 according to log-rank test.

Table S1: Primers used in this study.

Locus	Primer name	Sequence 5' → 3'
ASH1 *(FOXG_03647)*	Ash1_5F	GTAATACGACTCACTATAGGGAATATTCAACTAACTGCAACACATCG
	Ash1_5R	CGGGATCCACTTAACGTTACTGAAATCTTCGGCAGGGGCAAAGCTCG
	Ash1_3F	AATGCTCCTTCAATATCATCTTCTGTCGAATATCAGAGAGTTGAAGAC
	Ash1_3R	GACATAACTAATTACATGATGCGGCCCCTTGACCACCAAATAACAGC
	Ash1_5diag	CCTTGCTCTCCCAGCCTCTTCC
	Ash1_3diag	GCCGGCTCACCTGCACAGC
	Ash1_R	CATCGGCGACGGTAGTAGGAGG
	Ash1_compl_R	GAATGCACAGGTACACTTGTTTAGAGGCTAATCGTCTGCGACCACCC
	Ash1_OE_F	AACTCCATCACATCACAATCGATCCAAATGGCACTCTTATTGTCCTC
	Ash1_FLAG_R	GTAGTCGGAATTCTCGAGAAGCTTGACATCGTCTGCGACCACCCGAA
	Ash1_seq_R1	GGAGCTTGAAGTCGCGGCC
	Ash1_seq_R2	GCTGCTTGACTTCGCGAGGC
KMT6A *(FOXGR_ 002453)*	Kmt6a_5F	GTAATACGACTCACTATAGGGAATATTGATCTTCATGTGAGGCAGCC
	Kmt6a_5R	CGGGATCCACTTAACGTTACTGAAATCGGTTGCGGCTGATGACTTTG
	Kmt6a_3F	AATGCTCCTTCAATATCATCTTCTGTCTATAGAGGGTCTTCCAAGGTG
	Kmt6a_3R	GACATAACTAATTACATGATGCGGCCCCGCGACAACAGGGTTCTCGG
	Kmt6a_5diag	CGATCGCAGTTTAGCATCCGG
	Kmt6a_3diag	GCTGGACGCGGTAGACACGG
	Kmt6a_R	CCTGTCAAGTCGATGGTGTCAGG
	Kmt6a_compl_R	GAATGCACAGGTACACTTGTTTAGAGGTTATTGACTCCCGCTTATCC
	Kmt6a_TET_5R	GAATGCACAGGTACACTTGTTTAGAGGGGTTGCGGCTGATGACTTTG
	Kmt6a_TET_3F	TTGAGCAGACATCACCGTTTAAACACCATGGCTTCTCCTCATCTCGTC
	Kmt6a_TET_3R	GACATAACTAATTACATGATGCGGCCCGCTTAAAGCCAGATTTGCGG
	Kmt6a_seq_F1	GCCAGACCAGGAAGTGGTCGG
	Kmt6a_seq_R1	CACGATCTGGCGTTCTTCTGGG
	Kmt6a_seq_R2	CCTGGCTTCTCGTCGCACTCC
	Kmt6a_seq_R3	GCAAACTCGGAGCGCACTGC
	Kmt6a_5R_short	GGTTGCGGCTGATGACTTTG
KMT6B *(FOXG_17467)* ***KMT6C*** *(FOXG_07114)*	Kmt6b_5F	GTAATACGACTCACTATAGGGAATATTGTCCATCATGACTGCCTGGC
	Kmt6b-c_5R	GATCCACTTAACGTTACTGAAATCTGTCAAGGTTTTCTAGATTG
	Kmt6b_3F	AATGCTCCTTCAATATCATCTTCTGTCTAGGGGTGCTATACCATGTTG
	Kmt6b_3R	GACATAACTAATTACATGATGCGGCCCCATCGTCTGTGAGGATGTGG
	Kmt6c_5F	GTAATACGACTCACTATAGGGAATATTCGCGACGTGATTCGCGAACTG
	Kmt6c_3F	AGTGCTCCTTCAATATCATCTTCTGTCCAGGGGTGCTACACCATGTTG
	Kmt6c_3R	GACATAACTAATTACATGATGCGGCCCGATTGTTATCTATTGTGCGG
	Kmt6b_5diag	GATGGATCAGGCAAGCCATCAGG
	Kmt6b_3diag	GCTCTGAGTCGAGTTCAACGCG
	Kmt6c_5diag	CGTGATGTCGGTCAGTTTGCG
	Kmt6c_3diag	GCAAGATGTCGCACTTAGCGTGC
	Kmt6b-c_F	CAGATGCGACTGGGTCTTGCG
	Kmt6b-c_R	CCCAAGATCGCTTGAATCGTCC
	Kmt6b-c_seq_F1	GGAGCTGGAGGACATCGACCG
	Kmt6b-c_seq_F2	GATGCACGGTGGAGAATTGCG
	Pkmt6a_Kmt6b_F	GTTTGAGGCAAAGTCATCAGCCGCAACCATGATGTCAACCGCAGCCATG
	Pkmt6a_Kmt6b_R	CAGAATGCACAGGTACACTTGTTTAGAGGTCAATTCCTATCTTCTAAAC
	Pkmt6a_Kmt6c_F	GTTTGAGGCAAAGTCATCAGCCGCAACCATGTCAATCGCAGCCATGGC
	Pkmt6a_Kmt6c_R	CAGAATGCACAGGTACACTTGTTTAGAGGTCAATTCCCATCTTCTAAAC
FgKMT6 *(FGSC_15795)*	FgKMT6_Cil_XbaI_F1	AACGCCAGGGTTTTCCCAGTCTAGACACCCTTCTTGGATGACTCGCCACC
	FgKMT6_Cil_1R	TCTCCAGGTAGAGAAGGAC
	FgKMT6_Cil_2F	TCCTTCTCTACCTGGAGAG
	FgKMT6_Cil_2R	ATACATCTTATCTACATACGTTACTGACTGGCGCTCATCCAATCTCC
	FgKMT6_Cil_3F	AATATCATCTTCTGTCTCCGACGTTTCCGCTAGCTCGAACCAGG
	FgKMT6_Cil_XbaI_3R	AACAATTTCACACAGGAAACAGCTCTAGAACTTGCGGGCCTGGACTTCG
	FolKMT6A-C_CCil_1R	CTGCGGCTAATTGATGGCTTTGC
	FolKMT6A_CCil_2F	GCCATCAATTAGCCGCAGCCATGGCTTCTCCTCATCTCGTCG
	FolKMT6A_CCil_2R	CATACATCTTATCTACATACGTTATTGACTCCCGCTTATCCAGTCT
	FolKMT6B_CCil_2F	GCCATCAATTAGCCGCAGCCATGATGTCAACCGCAGCCATG
	FolKMT6B_CCil_2R	CATACATCTTATCTACATACGTCAATTCCTATCTTCTAAACCGC
	FolKMT6C_CCil_2F	CCATCAATTAGCCGCAGCCATGTCAATCGCAGCCATGGCTCC

	FolKMT6C_CCil_2R	CATACATCTTATCTACATACGTCAATTCCCATCTTCTAAACCGC
	FgKmt6_seq_F1	CTGCAGTCCTGCACCTACC
	FgKmt6_seq_F2	GATCATCACAGCGTTGTGCG
	FgKmt6_seq_F3	GTTGCTCAACTCCTTGACCG
	FgKmt6_seq_F4	ACTACGACCACAGTCTGAGG
SUZ12 *(FOXG_13918)*	Suz12_5F	GTAATACGACTCACTATAGGGAATATTCGTTCCACTGTCAGCAGCC
	Suz12_TET_5R	GAATGCACAGGTACACTTGTTTAGAGGGGCGGAGAAAAGCAATACC
	Suz12_TET_3F	TTGAGCAGACATCACCGTTTAAACACCATGACCCAGAAAAAGTCTTT
	Suz12_TET_3R	GACATAACTAATTACATGATGCGGCCCCCATTGACGCCGTTTGTGCC
	Suz12_5diag	GCTCACACTCACGCTCACACAG
	Suz12_R	GGCTCTTGGGCGTCGAGAGG
FTF1 *(FOXG_17458)*	Ftf1_OE_F	AACTCCATCACATCACAATCGATCCAAATGTCCGGCCGTGCCGTTTTG
	Ftf1_FLAG_R	GTAGTCGGAATTCTCGAGAAGCTTGACCCGGGTAGAGTGGGTAGAGC
	Ftf1_seq_F	CTTCGGAGCCAAAGATTGAG
	Ftf1_seq_R	TTCCTCCACTGATCGCAGTT
hphR/ natR	hph_F	GACAGAAGATGATATTGAAGGAGC
	hph_R	GATTTCAGTAACGTTAAGTGGAT
	TtrpC_R	CCTCTAAACAAGTGTACCTG
	trpC_P2	GTGATCCGCCTGGACGACTAAACC
	trpC_T	GGGCAAAGGAATAGAGTAG
	TtrpC_full_diag	CAGAATGCACAGGTACACTTG
	nat_seq_F	CGGACGGCGAGCGGCAGGCGC
	nat_seq_R	CTGGTGCGGTACCGGTAAGCC
genR	Tgluc_F2	CGTATGTAGATAAGATGTATG
	Gen_gpd_F	GTCGGAGACAGAAGATGATATTGAAGGAGCCAACAAAACACAGTTCCGACCAC
	Gen_seq_R3	CAGCCGATTGTCTGTTGTGC
	gen_PtrpC_F	CTATTCTACCGAAGCATCGATATCGATATGATTGAACAAGATGGATTG
	gen_TtrpC_R	GTTAAGTGGATCCCGGTCGGCATCTACTATTCAGAAGAACTCGTCAAG
	PtrpC_R	ATCGATATCGATGCTTCGGTAG
	TtrpC_F	AGTAGATGCCGACCGGGATCC
**pYES2**	pYES2_R	CTTCAGGTTGTCTAACTCCTTCC
**p*TET*^{OFF}**	hphR_PgpdA_F	AATGCTCCTTCAATATCATCTTCTGTCCGGAGAATATGGAGCTTCATC
	TET_off_R	GGTGTTTAAACGGTGATGTC
**pDDH-OGG/ pDDN-OGG**	Ddr_5F	GCCAGGGTTTTCCCAGTCACGACGCCGCGGACATAGGTATTAAACAGAAG
	Ddr_5R	GATTTGACAACCCCTTCCCCCCAACAAGATTTAGTAGTCGTTGTCACCAC
	Ddr_3F	CCGGGAACCAGTTAACGTTAACGTTTAAACGTATGATTGTTTGAGAGAGG
	Ddr_3R	CTTCGTTAAGCATCATGCCCGGGCCCGCTGTTTCCTGTGTGAAATTGTTA
	Ddr_5diag	GCCGATCCACATGATGAAGATGG
	Ddr_3diag	CAAGGGTATTCAGATCGTTGCGC
	hphR_PmeI_R	GTTTAAACGTTAACGTTAAC
	Tgluc_R	ATCTTGTTGGGGGGAAGGGG
	FLAG_Tgluc_F	GTCAAGCTTCTCGAGAATTCCGACTACAAGGACGACGATGACAAATAGCGTATG TAGATAAGATGTATGA
	FLAG_Tgluc_R	TCATACATCTTATCTACATACGCTATTTGTCATCGTCGTCCTTGTAGTCGGAATTCT CGAGAAGCTTGAC
	PoliC_seq_F2	GGGAGACGTATTTAGGTGCTAGGG
	Tgluc_seq_R2	CCGCCCTCTTTTGTCTTCCGC
**qRT-PCR**	Act_3qRT_F	ATGTCACCACCTTCAACTCCA
	Act_3qRT_R	CTCTCGTCGTACTCCTGCTT
	EF1a_3qRT_F	CGTCAAGATGGTTCCCTCCAAGC
	EF1a_3qRT_R	CCGCTGGACTTCTCGACAGCC
	EF1a_5qRT_F	CCTTAACGTCGTCGTCATCGGC
	EF1a_5qRT_R	CAGGTCTTGGTCGGGATTGACG
	SIX1_3qRT_F	ATAGCATGGTACTCCTTGGCG
	SIX1_3qRT_R	CCTGATGGTGACGGTTACGAA
	SIX1_5qRT_F	GCCAAGTCTACCGTCGACACATCC
	SIX1_5qRT_R	CGCAGCCTCTTGAGCATAAGCC
	SIX2_3qRT_F	TTCTACAGGGCGTCAATGGT
	SIX2_3qRT_R	GCAACGCCGTTTGAATAAGC
	SIX2_5qRT_F	CTGCACTTGAAACCCCTTAACTGCC
	SIX2_5qRT_R	CAGGTCGTAAATAGTGAGACCCACGC
	SIX3_3qRT_F	CGAGCTTCAGCACCGAACCT

	SIX3_3qRT_R	CGATCTCGTAGTTGGCGATG
	SIX3_5qRT_F	CCAGCCAGAAGGCCAGTTT
	SIX3_5qRT_R	CATGGACATGGCTATAAGCA
	SIX5_3qRT_F	CCTCTCCAGCATATTGGTT
	SIX5_3qRT_R	GTCAAAGCATGTTGAGTCTG
	SIX5_5qRT_F	CACGCTCTACTACTCTTCAGCGCAG
	SIX5_5qRT_R	CACACGAACTACACAGGCGTGTAGG
	FTF_3qRT_F	GATACTGCGCCTTTTCCTCG
	FTF_3qRT_R	ACGTTCTCCACCATCCAGTG
	FTF_5qRT_F	CGCCAGATTCTCGGCCTCG
	FTF_5qRT_R	CGTTCAATTCAGGCACGTCGC
	Kmt6a_3qRT_F	TCCTTCAGCTTCGGAATCGC
	Kmt6a_3qRT_R	GCCTGGATTCTTCAGTGCGT
	Kmt6b-c_3qRT_F	GCGTTAGGGAAGAGTTCGGG
	Kmt6b-c_3qRT_R	TGCTTTCTAGGCCTTCCACTG
	014750_5qRT_F	TGGCAAGGTTCCGTAGCAGTCG
	014750_5qRT_R	GCGGAACCCATGTTCCCGAG
	014750_3qRT_F	CAAGGCGTGGGATGATAACCTGG
	014750_3qRT_R	GGCAATCCCGCCATTCTCTCC
	015567_5qRT_F	CTGGATCCCGAAATCACGCATAC
	015567_5qRT_R	GGTGACTCGTCACGGTCGGC
	015567_3qRT_F	GGAAACCCGGAATGGATTACCG
	015567_3qRT_R	GCCACTCGAGACCCATCTCGC
	FgAct_qRT_F	TGGTGGTACCACCATGTACCCCG
	FgAct_qRT_R	GGGAAGCGAGAATGGAACCACCG
	FgKmt6_qRT_F	GCCACTACGACCACAGTCTGA
	FgKmt6_qRT_R	ATCGTCCTCGTTGTCTTCCTCC
	FgACT_3qRT_F2	ACGTCACCACTTTCAACTCCA
	FgEF1a_5qRT_R3	GGTTCGCTTGTCGATACCACCG
	FgFUS1_5qRT_F	CGAGTCAATCTTTCCCAAAAGAGC
	FgFUS1_5qRT_R	GGAATGGACGATTGAACGTCTCTC
	FgFUS1_3qRT_F	GTTCCTGTTGAAGATGTGGCGCAGGG
	FgFUS1_3qRT_R	CGAGTCAACCACTCCTCCACGCC
	FgZEA2_5qRT_F	CCTTCGAAAATGCTGGTAAGCCC
	FgZEA2_5qRT_R	GCGATCATCTCCTCACAAGTGCC
	FgZEA2_3qRT_F	CCACAGTCAGAGGTTGACCCAGG
	FgZEA2_3qRT_R	GCTCTCCATAGGCACTGCAGCC
	FgPKS2_5qRT_F	GCCATGATAGGGTACCGATGGAG
	FgPKS2_5qRT_R	CCAACGATTGCAATGTCACTCATC
	FgPKS2_3qRT_F	CCAAGCAAGCCACTGTCGTCG
	FgPKS2_3qRT_R	CTGCCAATGATATCGAACACGGC
	Gapdh-1	TGATGTTGAACTCGTCGCAG
	Gapdh-2	CCAAAAACAGTAACAGCCTTC
	Sl-PR1-Fw	TCTTGTGAGGCCCAAAATTC
	Sl-PR1-Rv	TAGTCTGGCCTCTCGGACA
	Sl-Chi3-Fw	TGCAGGAACATTCACTGGAG
	Sl-Chi3-Rv	TAACGTTGTGGCATGATGGT
	Sl-Actin-Fw	GGAATAGCATAAGATGGCAGACG
	Sl-Actin-Rv	ATACCCACCATCACACCAGTAT
	Fo-Erc1-qF	AGCTGGAGACTGATTTCGTG
	Fo-Erc1-qR	TCTTGACGGGGAGGTTACTG
	FOXG_00543 qPCR-F	CCCTCTCTTCTCACCAAAAC
	FOXG_00543 qPCR-R	AGCCATTGTTCTGAGTGTGAT
	FOXG_11153 qPCR-F	CTTCCTTCACCACCCGTTC
	FOXG_11153 qPCR-R	CCTGCTGTTGTTGCTGAATG
**Chromosome ends**	4.1_003315_F	GGTCGACATTGCCCTAGCAGAGG
	4.1_003315_R	GTGCCATAGCCTCAACGAGGTCC
	4.2_006163_F	GGCAGGATCAACATTCTTGTGGC
	4.2_006163_R	GCAGGTCAACCTCATCACACTCG
	5.1_006170_F	GTCTGCACATATCGCACTATCAACC
	5.1_006170_R	GTTATCTCCCACCTCGTCCTCTCC
	5.2_008776_F	CATCCCATTCCAGTTGGCACC

	5.2_008776_R	CGATTGTCCAGACAAGAACGTCG
	7.1_016039_F	GAGGGAGATGGCCATAGTATCGC
	7.1_016039_R	GCACCGCTGGGTATGTGTTAGC
	7.2_018474_F	GATGGCAGGACCACAGAAGTTGC
	7.2_018474_R	CTGAAACCTGGAAGTTCACCTGCG
	8.1_019995_F	GAGTCGGCACAGAGACAATTCGG
	8.1_019995_R	CCTCCGCAATTAGTCCACCAGG
	8.2_022114_F	GGAGCCGACATCACTGACGATACC
	8.2_022114_R	CTGGAGGAATGGTCTGAGAGACGG
	9.1_008883_F	GTCAGGCGGCATCATCGATCC
	9.1_008883_R	GGAGCATCTCAACGCCGTATGC
	9.2_010723_F	GGCCAGTCAGTTCGCTACAAGCC
	9.2_010723_R	CTCGGATAGACCGAGAATGTTGGC
	10.1_010745_F	CTCTACCGACCAGGCACCTGC
	10.1_010745_R	CTGAATTCCCTGCTTCTTACAGTGC
	10.2_012332_F	CTTCCGAGGAGGTTGCTGAACC
	10.2_012332_R	GCCCAGTCTTTCCGCATACACC
	11.1_025422_F	GCCTGAACAACAACGTTCACCG
	11.1_025422_R	CAGGATATTGCCCATCTTCAGCG
	11.2_026783_F	CAGGCAGAGAAGCAGTCTTCACC
	11.2_026783_R	CCTGCTTCATCGTATTGCTGTCG
	12.1_012364_F	CGAGAGACAGAACAATGGCTTGG
	12.1_012364_R	GCCGGATCTTGGAGTCTCGG
	12.1_FOXG_13376_F	GGTCGTTTGCTGATGAGAGGCC
	12.1_FOXG_13376_R	GCGAAGCACCGGTCTCATAGG
	12.1_FOXG_13385_F	CTCTCTGCGCCCACTCTGCC
	12.1_FOXG_13385_R	GCGAGTAGCGTTACCGAACGG
	12.1_FOXG_13408_F	GATCATGTCCTGGGCATCAATGC
	12.1_FOXG_13408_R	CTGGTGGATCAGCAGGTGGGC
	12.1_FOXG_13470_F	GGAAGAAATGGCGAGCGACC
	12.1_FOXG_13470_R	GGCCTTGCTTGCTGAGAAGAGG
	12.1_FOXG_13573_F	GTTGCCGGTGCAGGAGACACC
	12.1_FOXG_13573_R	GGTAGCCATGGAAGTGCGTTGG
	12.2_FOXG_14485_F	CAATGCCATGTCACCGATGGC
	12.2_FOXG_14485_R	GTCCAGCCAGCCCAGAAGAGTG
	12.2_013570_F	GGATCATCACGGCTACGACGG
	12.2_013570_R	GACGTCGATGGCGTTCCAGC
	13.1_013576_F	CATGCCACTGGTTCTGGCTGC
	13.1_013576_R	GTGGCCACCATCGTTGGAGC
	13.2_014744_F	GGATGAGCACCGTTATGTCCGC
	13.2_014744_R	CTCACGGCATAGACACCAATGAGG
	14.1_014750_F	CTCGGGAACATGGGTTCCGC
	14.1_014750_R	GGCAATCCCGCCATTCTCTCC
	14.2_015567_F	GCCGACCGTGACGAGTCACC
	14.2_015567_R	GCCACTCGAGACCCATCTCGC

Table S2: Gene Ontology (GO) enriched molecular functions of gene products differentially expressed in the RNA-seq analysis (Δ*ash1* and/or Δ*kmt6a* *vs.* WT under axenic conditions). Categories correspond to Figure 4. Genes lost from the genomes of Δ*ash1* or Δ*kmt6a* as found in the ChIP-seq were omitted in the analysis.

Category	ID	Description	GeneRatio	BgRatio	pvalue	p.adjust	qvalue	Count
**Genes deregulated in Δ*ash1* and/or Δ*kmt6a* (Figure 4): $\|log_2FC\| \geq 4$, FDR-corrected $P \leq 0.01$**								
**B (+,0)**	GO:0071949	FAD binding	21/466	137/9077	5.94E-06	1.21E-03	1.06E-03	21
	GO:0050660	flavin adenine dinucleotide binding	25/466	191/9077	1.41E-05	1.44E-03	1.26E-03	25
	GO:0005975	carbohydrate metabolic process	35/466	329/9077	3.08E-05	2.10E-03	1.83E-03	35
	GO:0020037	heme binding	26/466	227/9077	9.78E-05	4.99E-03	4.35E-03	26
	GO:0004553	hydrolase activity, hydrolyzing *O*-glycosyl compounds	22/466	181/9077	1.41E-04	5.75E-03	5.02E-03	22
	GO:0022857	transmembrane transporter activity	54/466	659/9077	3.62E-04	1.04E-02	9.05E-03	54
	GO:0008171	*O*-methyltransferase activity	6/466	20/9077	3.71E-04	1.04E-02	9.05E-03	6
	GO:0008199	ferric iron binding	4/466	8/9077	4.07E-04	1.04E-02	9.05E-03	4
	GO:0004560	alpha-L-fucosidase activity	3/466	4/9077	5.17E-04	1.17E-02	1.02E-02	3
	GO:0005506	iron ion binding	23/466	214/9077	6.12E-04	1.25E-02	1.09E-02	23
	GO:0004497	monooxygenase activity	20/466	180/9077	9.02E-04	1.67E-02	1.46E-02	20
	GO:0016705	oxidoreductase activity, acting on paired donors, with incorporation or reduction of molecular oxygen	20/466	186/9077	1.36E-03	2.30E-02	2.01E-02	20
	GO:0055085	transmembrane transport	60/466	817/9077	2.65E-03	4.15E-02	3.62E-02	60
**C (0,+)**	GO:0042910	xenobiotic transmembrane transporter activity	6/401	8/9077	1.86E-07	2.32E-05	2.14E-05	6
	GO:0016491	oxidoreductase activity	59/401	676/9077	2.49E-07	2.32E-05	2.14E-05	59
	GO:0015297	antiporter activity	6/401	10/9077	1.29E-06	8.02E-05	7.40E-05	6
	GO:0055085	transmembrane transport	61/401	817/9077	2.62E-05	1.22E-03	1.12E-03	61
	GO:1990961	xenobiotic detoxification by transmembrane export across the plasma membrane	4/401	6/9077	5.24E-05	1.84E-03	1.70E-03	4
	GO:0020037	heme binding	24/401	227/9077	6.21E-05	1.84E-03	1.70E-03	24
	GO:0016705	oxidoreductase activity, acting on paired donors, with incorporation or reduction of molecular oxygen	21/401	186/9077	6.94E-05	1.84E-03	1.70E-03	21
	GO:0004497	monooxygenase activity	20/401	180/9077	1.29E-04	2.99E-03	2.76E-03	20
	GO:0005506	iron ion binding	21/401	214/9077	4.94E-04	1.02E-02	9.42E-03	21
	GO:0015267	channel activity	3/401	7/9077	2.62E-03	4.88E-02	4.50E-02	3
**F (0,-)**	GO:0000981	DNA-binding transcription factor activity, RNA polymerase II-specific	80/326	703/9077	1.52E-21	2.26E-19	2.16E-19	80
	GO:0006355	regulation of DNA-templated transcription	89/326	882/9077	1.84E-20	1.37E-18	1.31E-18	89
	GO:0008270	zinc ion binding	94/326	1001/9077	1.76E-19	8.75E-18	8.34E-18	94
	GO:0006351	DNA-templated transcription	36/326	346/9077	6.97E-09	2.60E-07	2.48E-07	36
	GO:0003677	DNA binding	41/326	553/9077	6.67E-06	1.99E-04	1.90E-04	41
	GO:0022857	transmembrane transporter activity	40/326	659/9077	6.89E-04	1.71E-02	1.63E-02	40
	GO:0005254	chloride channel activity	2/326	2/9077	1.29E-03	2.74E-02	2.61E-02	2

Table S3: Gene Ontology (GO) enriched molecular functions of gene products differentially expressed in the RNA-seq analysis (Δ*kmt6a vs.* WT under axenic conditions and *in planta*). Genes lost from the genome of Δ*kmt6a* as found in the ChIP-seq were omitted in the analysis.

ID	Description	GeneRatio	BgRatio	pvalue	p.adjust	qvalue	Count
**Axenic cultivation - Genes upregulated in Δ*kmt6a*: |log_2FC| ≥ 1, FDR-corrected *P* ≤ 0.01**							
GO:0005975	carbohydrate metabolic process	89/1552	329/8969	4.56E-06	1.34E-03	1.24E-03	89
GO:0016407	acetyltransferase activity	28/1552	74/8969	1.99E-05	2.93E-03	2.70E-03	28
GO:0071949	FAD binding	43/1552	137/8969	3.56E-05	3.00E-03	2.76E-03	43
GO:0004553	hydrolase activity, hydrolyzing *O*-glycosyl compounds	53/1552	181/8969	4.07E-05	3.00E-03	2.76E-03	53
GO:0020037	heme binding	62/1552	227/8969	9.34E-05	5.51E-03	5.07E-03	62
GO:0015297	antiporter activity	7/1552	10/8969	3.38E-04	1.56E-02	1.44E-02	7
GO:0046983	protein dimerization activity	50/1552	182/8969	3.71E-04	1.56E-02	1.44E-02	50
GO:0042910	xenobiotic transmembrane transporter activity	6/1552	8/8969	5.42E-04	2.00E-02	1.84E-02	6
GO:0016705	oxidoreductase activity, acting on paired donors, with incorporation or reduction of molecular oxygen	50/1552	186/8969	6.41E-04	2.10E-02	1.93E-02	50
**Axenic cultivation - Genes downregulated in Δ*kmt6a*: |log_2FC| ≥ 1, FDR-corrected *P* ≤ 0.01**							
GO:0006351	DNA-templated transcription	108/1243	346/8969	1.80E-17	5.04E-15	5.03E-15	108
GO:0009116	nucleoside metabolic process	15/1243	41/8969	2.26E-04	3.15E-02	3.15E-02	15
***In planta* - Genes upregulated in Δ*kmt6a*: |log_2FC| ≥ 1, FDR-corrected *P* ≤ 0.01**							
GO:0015297	antiporter activity	7/895	10/8969	8.82E-06	2.04E-03	1.84E-03	7
GO:0042910	xenobiotic transmembrane transporter activity	6/895	8/8969	2.28E-05	2.63E-03	2.38E-03	6
GO:0016020	membrane	69/895	455/8969	2.26E-04	1.74E-02	1.57E-02	69
GO:0006694	steroid biosynthetic process	4/895	5/8969	4.54E-04	2.62E-02	2.36E-02	4
GO:0030151	molybdenum ion binding	6/895	13/8969	8.99E-04	3.46E-02	3.12E-02	6
GO:0071805	potassium ion transmembrane transport	6/895	13/8969	8.99E-04	3.46E-02	3.12E-02	6
GO:1990961	xenobiotic detoxification by transmembrane export across the plasma membrane	4/895	6/8969	1.25E-03	4.13E-02	3.73E-02	4
GO:0015079	potassium ion transmembrane transporter activity	5/895	10/8969	1.61E-03	4.64E-02	4.19E-02	5
***In planta* - Genes downregulated in Δ*kmt6a*: |log_2FC| ≥ 1, FDR-corrected *P* ≤ 0.01**							
GO:0005839	proteasome core complex	7/661	13/8969	1.33E-05	1.63E-03	1.47E-03	7
GO:0051603	proteolysis involved in protein catabolic process	7/661	13/8969	1.33E-05	1.63E-03	1.47E-03	7
GO:0006457	protein folding	11/661	35/8969	2.60E-05	2.13E-03	1.93E-03	11
GO:0005198	structural molecule activity	5/661	9/8969	2.10E-04	1.29E-02	1.17E-02	5
GO:0140662	ATP-dependent protein folding chaperone	8/661	25/8969	2.92E-04	1.44E-02	1.30E-02	8
GO:0051082	unfolded protein binding	7/661	20/8969	3.79E-04	1.55E-02	1.40E-02	7

GO:0005975	carbohydrate metabolic process	41/661	329/8969	5.82E-04	2.05E-02	1.85E-02	41
GO:0019773	proteasome core complex, alpha-subunit complex	4/661	7/8969	8.55E-04	2.63E-02	2.37E-02	4
GO:0016020	membrane	51/661	455/8969	1.56E-03	4.26E-02	3.84E-02	51
In planta **- Tomato genes upregulated during infection with Δ*kmt6a*: $\|log_2FC\| \geq 1$, FDR-corrected *P* ≤ 0.01**							
GO:0008295	spermidine biosynthetic process	2/76	5/16213	2.15E-04	2.88E-02	1.76E-02	2
GO:0000976	transcription cis-regulatory region binding	4/76	85/16213	6.77E-04	4.54E-02	2.78E-02	4
In planta **- Tomato genes downregulated during infection with Δ*kmt6a*: $\|log_2FC\| \geq 1$, FDR-corrected *P* ≤ 0.01**							
GO:0004867	serine-type endopeptidase inhibitor activity	7/189	31/16213	5.43E-08	1.09E-05	9.04E-06	7
GO:0005576	extracellular region	8/189	77/16213	3.11E-06	2.63E-04	2.19E-04	8
GO:0020037	heme binding	18/189	439/16213	3.95E-06	2.63E-04	2.19E-04	18
GO:0004568	chitinase activity	4/189	21/16213	9.17E-05	3.86E-03	3.21E-03	4
GO:0004601	peroxidase activity	8/189	123/16213	9.66E-05	3.86E-03	3.21E-03	8
GO:0005506	iron ion binding	13/189	334/16213	1.53E-04	4.52E-03	3.76E-03	13
GO:0030145	manganese ion binding	4/189	24/16213	1.58E-04	4.52E-03	3.76E-03	4
GO:0016829	lyase activity	5/189	50/16213	2.83E-04	7.07E-03	5.88E-03	5
GO:0050832	defense response to fungus	3/189	13/16213	4.09E-04	9.09E-03	7.56E-03	3
GO:0004497	monooxygenase activity	11/189	294/16213	6.83E-04	1.28E-02	1.06E-02	11
GO:0016705	oxidoreductase activity, acting on paired donors, with incorporation or reduction of molecular oxygen	11/189	295/16213	7.02E-04	1.28E-02	1.06E-02	11
GO:0006979	response to oxidative stress	7/189	131/16213	8.66E-04	1.44E-02	1.20E-02	7
GO:0006032	chitin catabolic process	3/189	18/16213	1.12E-03	1.68E-02	1.40E-02	3
GO:0016114	terpenoid biosynthetic process	4/189	40/16213	1.18E-03	1.68E-02	1.40E-02	4
GO:0009723	response to ethylene	3/189	19/16213	1.32E-03	1.76E-02	1.46E-02	3
GO:0010333	terpene synthase activity	4/189	44/16213	1.69E-03	1.97E-02	1.64E-02	4
GO:0008061	chitin binding	3/189	21/16213	1.78E-03	1.97E-02	1.64E-02	3
GO:0005199	structural constituent of cell wall	2/189	6/16213	1.97E-03	1.97E-02	1.64E-02	2
GO:0016717	oxidoreductase activity, acting on paired donors, with oxidation of a pair of donors resulting in the reduction of molecular oxygen to two molecules of water	2/189	6/16213	1.97E-03	1.97E-02	1.64E-02	2
GO:0042742	defense response to bacterium	2/189	6/16213	1.97E-03	1.97E-02	1.64E-02	2
GO:0016102	diterpenoid biosynthetic process	3/189	25/16213	2.97E-03	2.72E-02	2.26E-02	3
GO:0042744	hydrogen peroxide catabolic process	5/189	84/16213	3.00E-03	2.72E-02	2.26E-02	5
GO:0008283	cell population proliferation	2/189	8/16213	3.61E-03	3.14E-02	2.61E-02	2
GO:0009611	response to wounding	3/189	28/16213	4.12E-03	3.43E-02	2.86E-02	3
GO:0004620	phospholipase activity	2/189	9/16213	4.61E-03	3.55E-02	2.95E-02	2

GO:0080142	regulation of salicylic acid biosynthetic process	2/189	9/16213	4.61E-03	3.55E-02	2.95E-02	2
GO:0008083	growth factor activity	2/189	10/16213	5.72E-03	3.94E-02	3.28E-02	2
GO:0038199	ethylene receptor activity	2/189	10/16213	5.72E-03	3.94E-02	3.28E-02	2
GO:0051740	ethylene binding	2/189	10/16213	5.72E-03	3.94E-02	3.28E-02	2
GO:0048544	recognition of pollen	3/189	33/16213	6.58E-03	4.39E-02	3.65E-02	3
